# Supplementary material for: A Flipped Classroom Case to Introduce OB/GYN Clerkship Students to Contraception, Postpartum Care, and Intimate Partner Violence Screening
Source: MedEdPORTAL. 2025 Apr 9;21:11505. doi: 10.15766/mep_2374-8265.11505 (PMC11978902; doi:10.15766/mep_2374-8265.11505)
Supplement: Supplementary file 1 — Student Prework.docxContraception Cards.pptxPostpartum Slides.pptxFacilitator Guide.docxFacilitator Survey.docxStudent Survey.docx [file mep_2374-8265.11505-s001.zip › A. Student Prework.docx]

**Contraception, Postpartum Care, and Intimate Partner Violence Screening: A Flipped Classroom Case for OBGYN Clerkship Students**

*Student Pre-Work: To be completed by students prior to the start of the session,*

*laying foundational knowledge about each objective. It will take most students 3-4 hours to complete the prework.*

Session Objectives:

By the end of this activity, learners will be able to:

1. Outline the components of normal postpartum care.
2. Describe the mechanism of action and effectiveness of contraceptive methods.
3. Utilize national guidelines to determine the safety of contraceptive methods in key postpartum conditions.
4. Define basic evaluation and treatment of common postpartum abnormalities of the breast.
5. Identify wound complications and signs and symptoms of peripartum cardiomyopathy.
6. Compare the differences between postpartum blues, depression, and psychosis.
7. Summarize the approach to screening for intimate partner violence.

Pre-Work:

*Readings:*

- Chapter 26: Contraception. In: Casanova R, Chuang A, Goepfert AR, Hueppchen NA, Weiss PM, eds. Beckmann and Ling’s Obstetrics and Gynecology. 8^th^ ed. Wolters Kluwer; 2019: 225-238.
- ACOG Committee Opinion No. 518: Intimate Partner Violence
- Chapter 70: Postpartum Care. In: *ObGyn Secrets.* 4^th^ ed. Elsevier Inc; 2017: 322-325.
- ACOG Clinical Practice Guideline No. 4: Screening and diagnosis of mental health conditions during pregnancy and postpartum

*Download App:*

2016 US MEC (Medical Eligibility Criteria for Contraceptive Use) & US SPR (Selected Practice Recommendations for Contraceptive use)

Note:

Readings were provided to students as required pre-work. Student feedback has directed us to utilize brief, multimedia resources as an alternative option. These videos were created and are supported as official content through the Association of Professors of Gynecology & Obstetrics, the leading OBGYN undergraduate medical education organization. They are publicly available through their websites: <https://apgo.org/page/msostudent>

- Topic 13: Postpartum Care
- Topic 14: Lactation
- Topic 28: Postpartum Infection
- Topic 29: Anxiety and Depression
- Basic Science Topic 6: Contraception
- Topic 33: Family Planning
