# Supplementary material for: A Flipped Classroom Case to Introduce OB/GYN Clerkship Students to Contraception, Postpartum Care, and Intimate Partner Violence Screening
Source: MedEdPORTAL. 2025 Apr 9;21:11505. doi: 10.15766/mep_2374-8265.11505 (PMC11978902; doi:10.15766/mep_2374-8265.11505)
Supplement: Supplementary file 1 — Student Prework.docxContraception Cards.pptxPostpartum Slides.pptxFacilitator Guide.docxFacilitator Survey.docxStudent Survey.docx [file mep_2374-8265.11505-s001.zip › E. Facilitator Survey.docx]

**E. Facilitator/Clinical Instructor Survey**

Please complete the survey below. Thank you!

1. Please select the date of your session. ____________________
2. The faculty guide for this session helped me facilitate active learning.
   1. Strongly disagree
   2. Disagree
   3. Neither Agree nor Disagree
   4. Agree
   5. Strongly Agree
3. I spent less time preparing for this session than preparing for a traditional didactic lecture.
   1. Strongly disagree
   2. Disagree
   3. Neither Agree nor Disagree
   4. Agree
   5. Strongly Agree
4. The information included in the faculty guide helped increase my confidence in teaching subjects outside of my area of expertise.
   1. Strongly disagree
   2. Disagree
   3. Neither Agree nor Disagree
   4. Agree
   5. Strongly Agree
5. If you have any other comments about this session, please share your feedback. __________________
