# Supplementary material for: A Flipped Classroom Case to Introduce OB/GYN Clerkship Students to Contraception, Postpartum Care, and Intimate Partner Violence Screening
Source: MedEdPORTAL. 2025 Apr 9;21:11505. doi: 10.15766/mep_2374-8265.11505 (PMC11978902; doi:10.15766/mep_2374-8265.11505)
Supplement: Supplementary file 1 — Student Prework.docxContraception Cards.pptxPostpartum Slides.pptxFacilitator Guide.docxFacilitator Survey.docxStudent Survey.docx [file mep_2374-8265.11505-s001.zip › F. Student Survey.docx]

**F. Student Survey**

Please complete the survey below. Thank you!

1. Please select the date of your SLT session. _________________________
2. The assigned pre-work gave me the background knowledge needed to participate in activities during this session.
   1. Strongly Disagree
   2. Disagree
   3. Neutral
   4. Agree
   5. Strongly Agree
3. The session was interactive.
   1. Strongly Disagree
   2. Disagree
   3. Neutral
   4. Agree
   5. Strongly Agree
4. The format of this session will help me apply what I learned to patient care.
   1. Strongly Disagree
   2. Disagree
   3. Neutral
   4. Agree
   5. Strongly Agree
5. This session helped me understand the mechanism of action, effectiveness, and safety of contraceptive methods.
   1. Strongly Disagree
   2. Disagree
   3. Neutral
   4. Agree
   5. Strongly Agree
6. This session helped me understand the components of normal postpartum care.
   1. Strongly Disagree
   2. Disagree
   3. Neutral
   4. Agree
   5. Strongly Agree
7. After this session, I feel more comfortable diagnosing and treating common postpartum abnormalities of the breast.
   1. Strongly Disagree
   2. Disagree
   3. Neutral
   4. Agree
   5. Strongly Agree
8. After this session, I feel more comfortable evaluating a suspected postpartum complication.
   1. Strongly Disagree
   2. Disagree
   3. Neutral
   4. Agree
   5. Strongly Agree
9. This session helped me understand the difference between postpartum blues, depression, and psychosis.
   1. Strongly Disagree
   2. Disagree
   3. Neutral
   4. Agree
   5. Strongly Agree
10. After this session, I feel more comfortable screening for domestic violence.
    1. Strongly Disagree
    2. Disagree
    3. Neutral
    4. Agree
    5. Strongly Agree
