# Supplementary material for: A Flipped Classroom Case to Introduce OB/GYN Clerkship Students to Contraception, Postpartum Care, and Intimate Partner Violence Screening
Source: MedEdPORTAL. 2025 Apr 9;21:11505. doi: 10.15766/mep_2374-8265.11505 (PMC11978902; doi:10.15766/mep_2374-8265.11505)
Supplement: Supplementary file 1 — Student Prework.docxContraception Cards.pptxPostpartum Slides.pptxFacilitator Guide.docxFacilitator Survey.docxStudent Survey.docx [file mep_2374-8265.11505-s001.zip › D. Facilitator Guide.docx]

| Slide 1 |  | | **Facilitator Info**:  This session invites you to follow our patient, Gynnifer (she/her), through her postpartum state beginning 6 weeks after a vaginal delivery of twins. She lives with her husband and twin girls. You will need to consider appropriate contraception options and screen for intimate partner violence to provide simulated care.  This facilitator guide will provide you with background information about the topics presented, supply answers to common questions students may ask during the session, and also includes student prompts to encourage active learning during the session. The first time the session is guided, it may take 1-2 hours to review content depending on baseline facilitator knowledge and clinical experience. Subsequent sessions are typically guided without additional time to prepare. | |
| --- | --- | --- | --- | --- |
| Slide 2 |  |  | | |
| Slide 3 |  | | | **Student Engagement:**  Ask each student to list something important to address at the postpartum visit. The next slide uses a mnemonic of 7 “B’s” to help students remember important things to ask postpartum patients. |
| Slide 4 |  | | | **Facilitator Info:**   - The 7 “B”s is a mnemonic to help students remember issues that are helpful to address with postpartum patients. - Breast/formula feeding – Ask patients about any pain or symptoms they are having and assess whether they might be candidates for lactation consultation or additional feeding assistance. Note that “chestfeeding” is a gender-neutral term; transgender patients should be asked what they prefer. - Baby’s health – Ensure that they are connected with a pediatrician who can provide preventive and other care for the baby, including any health challenges they may have and mental health or social support resources. - Birth control – Later slides in this session focus in depth on this. - Bleeding – It is helpful to remind students that bleeding is expected to be resolved by six week postpartum and continued bleeding should prompt evaluation of retained products of conception, gestational trophoblastic neoplasia, or other etiology. - Blues – Postpartum mental health is addressed later in this session - “Boinking” – Important to educate about the impact of breast/chestfeeding, which can cause atrophy and menopausal type symptoms, and anatomic relaxation or lacerations which often alter the way patients feel, experience sex, or urinary leakage after birth. - Boosters – Ensure patients have had indicated vaccinations including Tdap, Flu, and Covid vaccines.   **** Additional Facilitator Info:** For more information about postpartum care, please see:  Optimizing postpartum care. ACOG Committee Opinion No. 736. American College of Obstetricians and Gynecologists. Obstet Gynecol 2018;131:e140-50.^1^ |
| Slide 5 |  | | | **Student Engagement:**  Use the contraception cards, and have students work as a single group to categorize the cards in order of efficacy.  **Facilitator Info:**   - Efficacy rates are covered on the next slide. Students can gauge how accurate they were in the exercise. - When they have completed the exercise, consider the following reflective question to the group: Did you use perfect use or typical use failure rates to complete the exercise?   **Perfect use** refers to failure rates documented in clinical trials under highly controlled circumstances.  **Typical use** refers to failure rates documented in post-marketing research and is more reflective of average failure rates that most users can expect.  Usually, you would use the typical use failure rate when counseling a patient about their options. For example, abstinence is the most effective method based on perfect use but much less effective based on typical use.     - Please help students understand that while we expect them to know the efficacy of various methods and be able to discuss this with patients, it is very important to use a patient-centered counseling model when working with a patient to select a method. Patients may not find efficacy of methods to be the most important characteristic in their contraceptive decision-making and counseling should be tailored based on what they indicate is most important to them (ex. bleeding side effects, ability to control initiation and discontinuation, etc.) |
| Slide 6 |  | | | **Facilitator Info:** Orient students to this slide:   1. The bottom row includes the least effective methods, resulting in 18 or more pregnancies per year for every 100 women using the method. **Note**: Condoms are in this group. Condoms are very common and many patients may overestimate their effectiveness. 2. The second row includes moderately effective methods. Point out that patients are often unaware that nearly 10% of pill users experience pregnancy over a year of use. 3. The top row reflects methods with highest efficacy. IUDs and implants are much more effective than pills and equally effective as permanent sterilization.   Mechanism of Action: While reviewing this slide, be sure students know the basic mechanism of action of each method.  **Student Engagement:**  Ask the group to list the methods that work by inhibition of ovulation.  **Answer:**  -Inhibit ovulation: Implant, Injection, Pill, Ring, Patch  -Some people who use progesterone IUDs will not ovulate, but its primary mechanism of action is thickening of cervical mucus which is impenetrable to sperm -Copper IUD works because copper is a potent spermicide.  ****Additional Facilitator Info:**  For more information about gender-affirming contraception efficacy, perfect and typical use, please see:  Document by Reproductive Health Access Project, retrieved from [https://www.reproductiveaccess.org/contraception/](https://urldefense.com/v3/__https:/www.reproductiveaccess.org/contraception/__;!!Ab1_Rw!GO26JbWtEEvbAnKtJyUs_B-cRD4zwDw50isoNsq_oKNng12-O0iQyJ4pSfTHysyXacKyEvpygzNwZFmE$) on October 2, 2024. Creative commons license associated: https://creativecommons.org/licenses/by-nc-sa/4.0/^2^ and  Family planning: a global handbook for providers (2011 update). Baltimore, MD; Geneva, Switzerland: CCP and WHO; 2011; and Trussell J. Contraceptive failure in the United States. Contraception 2011; 83: 397–404.^3^ |
| Slide 7 |  | | | **Facilitator Info:**  Students may download and use the free US Medical Eligibility Criteria app in advance, can look up the chart on their device, or you can provide them with a printout of the chart included in the appendix. |
| Slide 8 |  | | | **Facilitator info:**   - Remind students we have talked about mechanism of action and efficacy but it is important to understand the safety of contraceptive methods as well. - The US Medical Eligibility Criteria (MEC) is a comprehensive resource that summarizes available evidence on the safety of various methods in the setting of various health conditions. We teach medical students about this because they can utilize this resource readily in whatever specialty they pursue to help with contraceptive care for their patients, especially when new medical conditions arise for a patient. - The guidance uses a rating system of 1-4 outlined on the slide.  - Generally, categories 1 and 2 can be thought of as a “green light” in which use is safe. - Category 4 is a “red light” in which use is not safe. - Note that a category “3” is acceptable for use if there is *not* an acceptable alternative and benefits>risks. This is a category that requires clinical judgement and careful counseling.   The next slide outlines an interactive learning exercise to apply these criteria in the postpartum patient.  **** Additional Facilitator Info:** For more information on the US MEC, please see: Curtis KM, Tepper NK, Jatlaoui TC, et al. U.S. Medical Eligibility Criteria for Contraceptive Use, 2016. MMWR Recomm Rep 2016;65(No. RR-3):1-104.^4^ |
| Slide 9 |  | | | **Student Engagement:**  Divide your small group into 3 subgroups and assign each group one of the clinical questions on this slide. They should use the MEC to identify the safety of each method for the patient. After clarifying groups and assignments, go to next slide for a table to help students organize their thinking.  **Facilitator Note:** Be sure to tell Group 1: When” breastfeeding” is searched, there will not be a category listed for IUDs, however when “postpartum” is searched, there is a category specifically for IUDs with this information included. |
| Slide 10 |  | | | **Facilitator Info:**  The purpose of this slide is to help students organize their thinking while working through their assignments. The next slide has answers. |
| Slide 11 |  | | | **Facilitator info:**   - Let students benchmark their own researched answers against this chart. - Point out that almost all methods for these postpartum scenarios are category 1 or 2, meaning safe to use. - Methods containing estrogen are contraindicated (cat 4) in the setting of acute DVT because estrogen is associated with an increased risk of venous thromboembolism. - Estrogen-containing methods are a category 3 because estrogen increases risk of arterial thromboembolism like stroke and MI and hypertension also increases this risk. When hypertension is well controlled, the risk of stroke and MI should be lower. However, non-estrogen containing methods would be safer in this clinical situation. |
| Slide 12 |  | | | **Facilitator Info:**   - Encourage all students to use patient-centered and shared decision-making models for contraception counseling. These models treat the patient as an expert in their own life and elicit their individualized priorities for contraception choices. As the medical expert, the provider can review available methods through the lens of the patient’s priorities and answer questions to help them make a good choice for themselves. - Characteristics of contraception that are potentially important to discuss with patients include: - Efficacy - Compliance - Contraindications - Side effects - Previous experience with contraceptives |
| Slide 13 |  | | | **Facilitator Info:**   - In the postpartum patient, it is also important to review recommended pregnancy spacing of 18 months to optimize maternal and child health in subsequent pregnancies. - Potential adverse outcomes include: maternal anemia, preterm birth, low birth weight, PPROM, placental abruption, pre-eclampsia, failed TOLAC   **** Additional Facilitator Info:**  For more information on interpregnancy care, please see:  Interpregnancy care. Obstetric Care Consensus No. 8. American College of Obstetricians and Gynecologists. Obstet Gynecol 2019;133:e51-72.^5^ |
| Slide 14 |  | | | **Student Engagement:**  Split students into the same groups as before. Give them 1 minute to write down as many postpartum complications as they can. Extensive list is on next slide. |
| Slide 15 |  | | | **Facilitator Info:**   - This list contains numerous complications that patients may experience in the postpartum period. - We can’t cover all of these complications in small group but it is important for students to educate themselves and for medical students, these complications these topics may appear on end of clerkship shelf exam. - In summary, postpartum patients may experiences complications associate with breast health, wounds (both cesarean and vaginal laceration wounds), uterine infection like endometritis, thromboembolism, cardiomyopathy, hypertensive disorders like pre-eclampsia, mental health disorders, and changes associated with anatomic changes resulting from pregnancy and delivery such as incontinence.   **** Additional Facilitator Info:**  For more information on Postpartum Complications, please see:  Valderramos, S. Chapter 70: Postpartum Care. In: *ObGyn Secrets.* 4^th^ ed. Elsevier Inc; 2017: 322-325.^6^ |
| Slide 16 |  | | | **Facilitator info:**   - Orient students to this graph. The Y axis represents various stats from non-pregnant, non-contraceptive users to postpartum patients. The x axis is absolute number of VTE episodes per 10,000 women in the population. - Several helpful learning points to highlight on this slide include  1. VTE is much more common in pregnancy and postpartum than in combined contraceptive users. This is important in helping patients consider their decision making related to contraception options and providers must sometimes weigh the risks of using contraception against the risks of pregnancy/postpartum state. 2. When discussing risk with patients, use absolute risk instead of relative risk to avoid sensationalizing the risks to patients. Ex. The relative risk of VTE risk with COC use is 2-3 times higher than in non-users. However, the absolute risk is 3-9 cases per 10,000 users. An individual may be frightened to hear 3 times higher risk but reassured by 9 cases per 10,000 people. |
| Slide 17 |  | | | **Facilitator info:**   - Teaching points on this slide include: -- Have a high suspicion for VTE in pregnant and postpartum patients as the risk is 4-5x greater compared to non-pregnant state. -- The first 6 weeks postpartum carries the greatest risk. -- Failure to consider VTE among this group is devastating as it is one of the leading causes of maternal mortality in our country. |
| Slide 18 |  | | | **Student Engagement:**  1) Ask students to scan this list of VTE risk factors and say which our patient has. (Answer: recent multiple gestation and pre-eclampsia)  2) Post the question on the bottom of the slide to the group and request they answer before advancing slides. (Coagulation system changes summarized on next slide.)  **** Additional Facilitator Info:**  For more information on Thromboembolism in Pregnancy, please see:  Thromboembolism in pregnancy. ACOG Practice Bulletin No. 196. American College of Obstetricians and Gynecologists. Obstet Gynecol 2018;132:e1-17.^7^ |
| Slide 19 |  | | | **Facilitator info:** Summarize that numerous procoagulants increase during pregnancy while protein S, an anticoagulant, decreases during pregnancy. The sum is a significant increase in coagulability during pregnancy and the postpartum period. |
| Slide 20 |  | | | **Facilitator Info:**  Switching gears, our patient also mentions a breast complaint.  **Student Engagement:**  Have students list additional history they need to gather in order to formulate a differential diagnosis.  Additional questions:   - OLD CARTS (our students are taught this mnemonic to use when obtaining an history of present illness. Onset, Location, Duration, Characteristic, Alleviating and Aggravating factors, Radiation, Timing, Severity) - Fever? - Possible engorgement? - Flu-like symptoms? - Nipple changes or concerns? - Frequency and duration of breast-feeding?   Ask students to generate a differential diagnosis before you advance the slide (the next 2 slides cover answer) |
| Slide 21 |  | | | **Facilitator info:**   - Cracked nipples, engorgement, and clogged ducts are non-infectious etiologies of breast symptoms in nursing people. - Review the characteristics of each issue outlined on the slide. - Clogged duct is often focal and some describe it as feeling like they’ve been kicked in the breast.   (Continued on next slide)  **** Additional Facilitator Info:**  For more information on Breastfeeding Challenges, please see:  Mitchell KB, Johnson HM, Rodriguez JM, Eglash A, Scherzinger C, Widmer K, Berens P, Miller B, and the Academic of Breastfeeding Medicine. Academy of Breastfeeding Medicine Clinical Protocol #36: The Mastitis Spectrum, Revised 2022. Breastfeeding Medicine. May 2022.360-376.^8^ |
| Slide 22 |  | | | **Facilitator info:**   - Mastitis and abscess are inflammatory/infectious etiologies of breast symptoms. Mastitis is more common than abscess. Emphasize that it is ok and recommended to continue nursing when you have mastitis. Mastitis has flu-like symptoms, while clogged duct does not. Question stems often describe a “wedge-shaped area of redness” when referring to mastitis. - If conservative care with mastitis is not effective, antibiotics should be initiated. If there is still not improvement, the patient should be assessed for possible abscess as this may require drainage as well as antibiotics. |
| Slide 23 |  | | | **Facilitator info:**  This slide summarize that our patient had mastitis and responded to conservative therapy. If you didn’t already, emphasize that we advise patients to continue breast/chestfeeding in the setting of mastitis or abscess. |
| Slide 24 |  | | | **Facilitator info:**  This slide aims to help students differentiate among various complications that can occur postoperatively in patients who have undergone a Cesarean delivery.  **Student Engagement:**  Have students complete this matching game. The animation on the slide connects the complication with its description.  Answers and teaching points:  1) Hematoma – E – a collection of blood underneath a wound that has been sutured closed  2) Seroma – B – a collection of serum underneath a wound that has been sutured closed  Hematoma and Seroma can serve as a nidus for bacterial growth. Simply draining them may be sufficient for treatment. Often incisions are allowed to heal by secondary intention once drained.  3) Dehiscence – G – fascial disruption due to abdominal wall tension overcoming tissue or suture strength or knot security. Dehiscence requires surgical repair. 4) Superficial wound infection – A – infection involving the skin or subcutaneous tissue 5) Deep wound infection – F – infection involving the deep soft tissues of the incision such as the fascia and muscle layers 6) Necrotizing fasciitis – C – rapid and progressive necrosis of subcutaneous tissue and fascia. Necrotizing fasciitis is an emergency. The other infections typically respond to antibiotic therapy though deep wound infections may also require surgical debridement 7) Endometritis – D - results from polymicrobial infection of the decidua, characterized by fever, fundal tenderness, and purulent discharge from the uterus |
| Slide 25 |  | | | **Facilitator info:** Peripartum cardiomyopathy is a dramatic complication sometimes seen in pregnancy.  Patients who experience this should be counseled about their increased risk in subsequent pregnancies, including maternal mortality. |
| Slide 26 |  | | |  |
| Slide 27 |  | | | **Facilitator info:**  Gynnifer discloses the symptoms on the slide to us.  **Student Engagement:**   - Ask each student to develop a differential diagnosis given these symptoms. - Ask a student to share their differential with the group. Next, ask others to share whether they included anything additional in their differential. - These will be discussed in the next few slides in more detail. - While we will focus on mental health here in small group, remind students not to forget about including anemia and thyroid disease in their differential diagnosis. |
| Slide 28 |  | | | **Facilitator info:** With the given symptoms, the three main mental health etiologies on our differential include blues, depression, and psychosis. Our job as providers is to differentiate among these and provide the appropriate support.  ****Additional Facilitator Info:**  For more information on Peripartum Mood Disorders, please see: Screening and diagnosis of mental health conditions during pregnancy and postpartum. Clinical Practice Guideline No. 4. American College of Obstetricians and Gynecologists. Obstet Gynecol 2023;141:1232-61.^9^ |
| Slide 29 |  | | | **Student Engagement:**  - Ask students to list important follow-up questions to Gynnifer’s complaint of depressed feelings.  -Remind students that the questions they ask should help them discern from among the items on the differential diagnosis and determine any need for emergency care.  -Some key questions include:   - Have you had any thoughts about harming yourself or the baby? - How are you sleeping? - How often do you experience crying spells? - What other symptoms have you noticed? (guilt, irritability, anxiety, fear) - Personal or family history of mood disorders? |
| Slide 30 |  | | | **Facilitator info:** To follow up on the list of additional questions generated with the previous slide, this slide reviews Gynnifer’s response to some of those questions:  Her dominant symptoms are difficulty sleeping, frequent crying spells, guilt and irritability.  These have been present for the last few weeks.  She denies personal history of mood disorder but her mother experienced postpartum depression. |
| Slide 31 |  | | | **Facilitator info:** Share that “baby blues” are very common in the first few days after delivery and usually improve without treatment. |
| Slide 32 |  | | | **Facilitator info:** Be on the lookout for postpartum depression in the first year after delivery, but it usually starts soon after.  PPD meets DSM criteria for major depression as outlined on the slide.  Postpartum Depression is treated as other depression with therapy +/- medication. |
| Slide 33 |  | | | **Facilitator info:** Psychosis is uncommon but is an emergency! Patient and child safety must be addressed. |
| Slide 34 |  | | | **Facilitator info:**  There are multiple validated postpartum depression questionnaires; the EPDS is the one we use in our office.  Patient fills out the questionnaire prior to provider visit. |
| Slide 35 |  | | | **Facilitator info:**   - We are going to cover an overview of IPV here but acknowledge that this is NOT sufficient didactic information to stand alone for learning in this clerkship or on this topic. Remind students that the pre-work and other learning materials will provide additional information. - Intimate partner violence (IPV) is even more common during pregnancy than at baseline. - Screening for IPV is an important skill to gain during the reproductive health rotation. - Mention to students that it can take years and exposure to dozens of patients before gaining confidence in addressing IPV with patients. If a student suspects this, they should involve their preceptor ASAP to assist.   **** Additional Facilitator Info:**  For more information on Intimate Partner Violence, please see:  Intimate Partner Violence. Committee Opinion No. 518. American College of Obstetricians and Gynecologists. Obstet Gynecol 2012;119:412-7.^10^ |
| Slide 36 |  | | | **Facilitator info:**  Important elements to include when screening for IPV:   - Create a safe space for the patient - Normalize the screening and ensure that patients know you ask everyone - Center their well-being and that of their baby in the postpartum context - Reassure them about confidentiality |
| Slide 37 |  | | | **Facilitator Info:**   - Even though there is no gold standard, it is important to assess this routinely using some systemic approach. - Written screening tools may be more likely to elicit the information than face to face questions. Including a question on your written intake form is one way to give patients an opportunity to bring this up as a concern for the visit, but remember to review the form and address it as needed. - If you use face to face questioning, use open-ended screening questions. Use terms like “trauma”, “hurt”, ”injured” and not “victim” or “domestic violence.” |
| Slide 38 |  | | | **Facilitator info:** Inform students that there are multiple tools that could be used. This is one framework we use and teach.  R – Remember that it is important to screen routinely! Written screenings (ex. intake forms) are an important tool as patients may disclose IPV more readily in that format.  A- Go beyond simply asking if they feel safe in their environment. Specifically ask if they have been hit, kicked, etc  D- Document your suspicion as this may be important in the patient’s future care or any legal cases that result.  A – It is very important to be sure that you review safety when IPV is occurring! Deadly violence is more likely if there are guns or other weapons in the home. If the violent outbursts or actions have been increasing or growing more severe, the risk of serious injury or death increases.  R - Regardless of the patient’s readiness to leave the situation, be sure to provide resources and to give some suggestions about an exit plan. Involve social worker if patient is open to it. |
| Slide 39 |  | | | **Facilitator info:** This slide provides an opportunity to highlight which populations are at increased risk of IPV and warning signs that should especially prompt screening. |
| Slide 40 |  | | | **Facilitator info:** Reminders: It is good practice to have a list of local and national resources handy that can be readily provided to patients as needed.  Express empathy and reassure that you will continue close follow-up and help identify resources.  Engage the interdisciplinary team members in your setting to help access safety resources, counseling resources, etc. In many settings, including ours, this is our social worker.  Apply the principles of trauma-informed care for this patient going forward, emphasizing “safety” and “agency” and exploring what things would make them feel safe and comfortable during future visits. |
| Slide 41 |  | | | **Facilitator info:** This slide summarizes the conclusion of our patient’s care today. |

**References and Additional Resources:**

1. Optimizing postpartum care. ACOG Committee Opinion No. 736. American College of Obstetricians and Gynecologists. Obstet Gynecol 2018;131:e140-50.
2. Document by Reproductive Health Access Project, retrieved from [https://www.reproductiveaccess.org/contraception/](https://urldefense.com/v3/__https:/www.reproductiveaccess.org/contraception/__;!!Ab1_Rw!GO26JbWtEEvbAnKtJyUs_B-cRD4zwDw50isoNsq_oKNng12-O0iQyJ4pSfTHysyXacKyEvpygzNwZFmE$) on October 2, 2024. Creative commons license associated: https://creativecommons.org/licenses/by-nc-sa/4.0/
3. Family planning: a global handbook for providers (2011 update). Baltimore, MD; Geneva, Switzerland: CCP and WHO; 2011; and Trussell J. Contraceptive failure in the United States. Contraception 2011; 83: 397–404.
4. Curtis KM, Tepper NK, Jatlaoui TC, et al. U.S. Medical Eligibility Criteria for Contraceptive Use, 2016. MMWR Recomm Rep 2016;65(No. RR-3):1-104.
5. Interpregnancy care. Obstetric Care Consensus No. 8. American College of Obstetricians and Gynecologists. Obstet Gynecol 2019;133:e51-72.
6. Valderramos, S. Chapter 70: Postpartum Care. In: *ObGyn Secrets.* 4^th^ ed. Elsevier Inc; 2017: 322-325.
7. Thromboembolism in pregnancy. ACOG Practice Bulletin No. 196. American College of Obstetricians and Gynecologists. Obstet Gynecol 2018;132:e1-17.
8. Mitchell KB, Johnson HM, Rodriguez JM, Eglash A, Scherzinger C, Widmer K, Berens P, Miller B, and the Academic of Breastfeeding Medicine. Academy of Breastfeeding Medicine Clinical Protocol #36: The Mastitis Spectrum, Revised 2022. Breastfeeding Medicine. May 2022.360-376
9. Screening and diagnosis of mental health conditions during pregnancy and postpartum. Clinical Practice Guideline No. 4. American College of Obstetricians and Gynecologists. Obstet Gynecol 2023;141:1232-61.
10. Intimate Partner Violence. Committee Opinion No. 518. American College of Obstetricians and Gynecologists. Obstet Gynecol 2012;119:412-7.
