# Supplementary material for: A Flipped Classroom Case to Introduce OB/GYN Clerkship Students to Contraception, Postpartum Care, and Intimate Partner Violence Screening
Source: MedEdPORTAL. 2025 Apr 9;21:11505. doi: 10.15766/mep_2374-8265.11505 (PMC11978902; doi:10.15766/mep_2374-8265.11505)
Supplement: Supplementary file 1 — Student Prework.docxContraception Cards.pptxPostpartum Slides.pptxFacilitator Guide.docxFacilitator Survey.docxStudent Survey.docx [file mep_2374-8265.11505-s001.zip › B. Contraception Cards.pptx]

## Slide 1
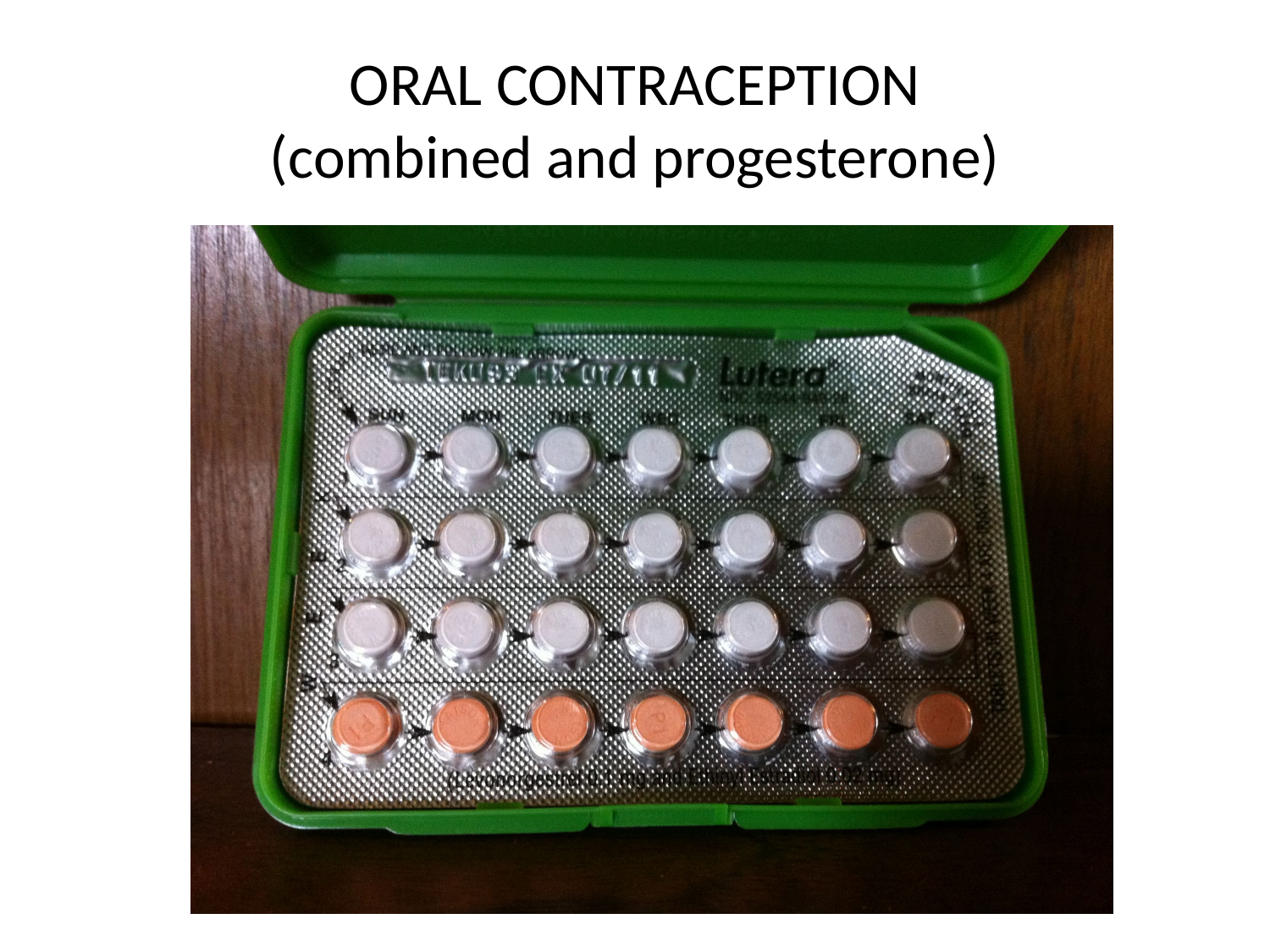

# ORAL CONTRACEPTION (combined and progesterone)

## Slide 2
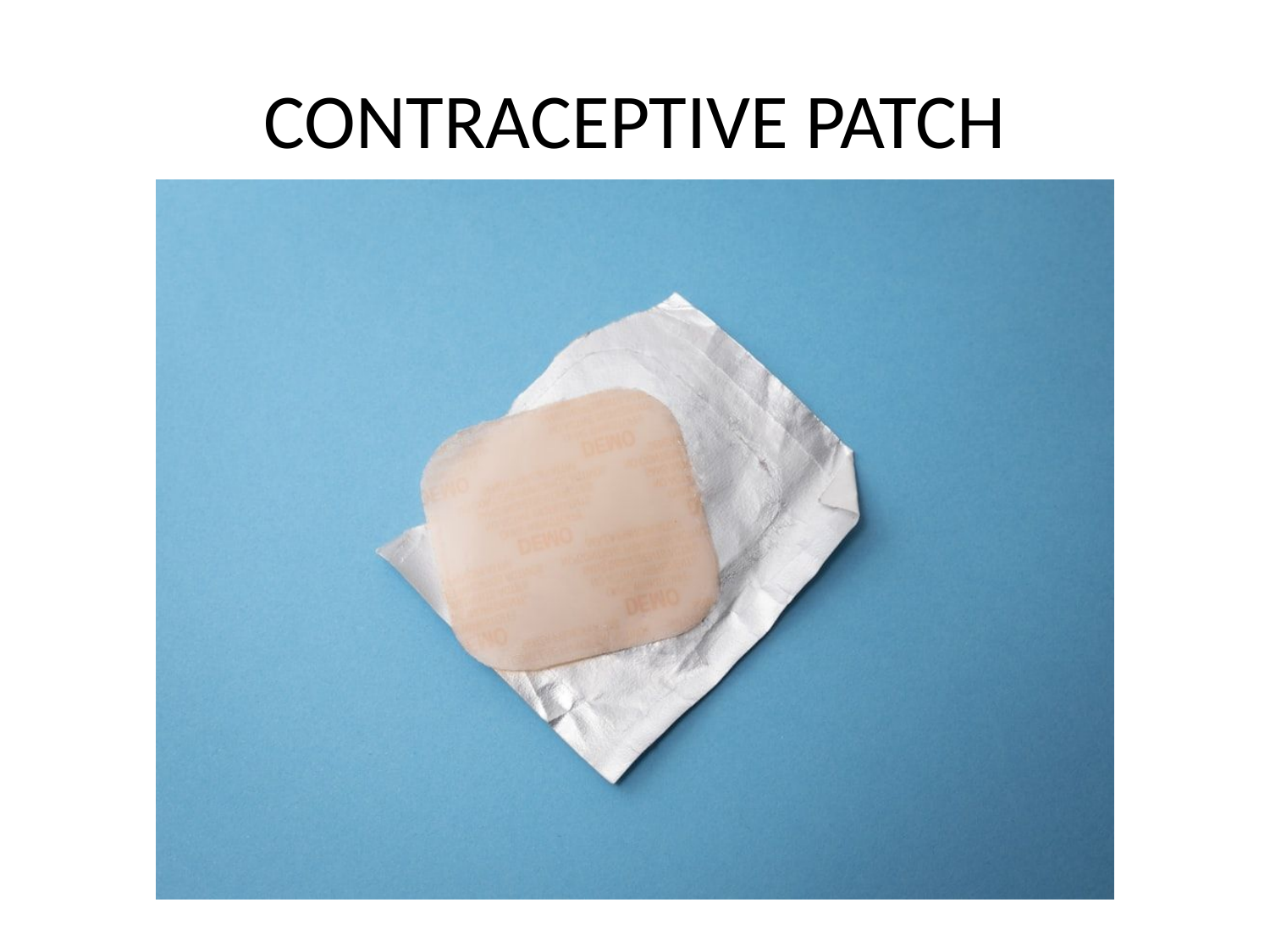

# CONTRACEPTIVE PATCH

## Slide 3
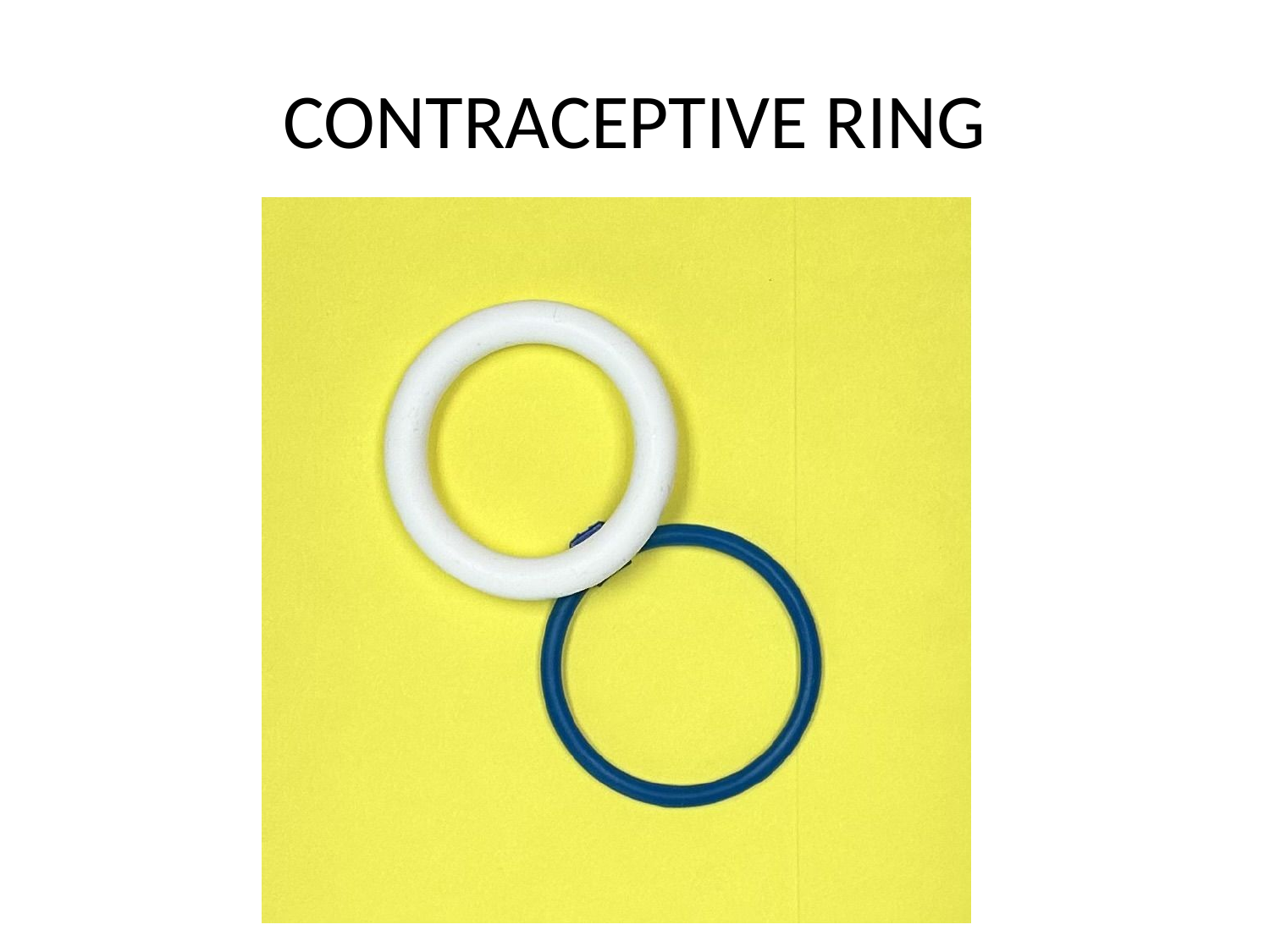

# CONTRACEPTIVE RING

## Slide 4
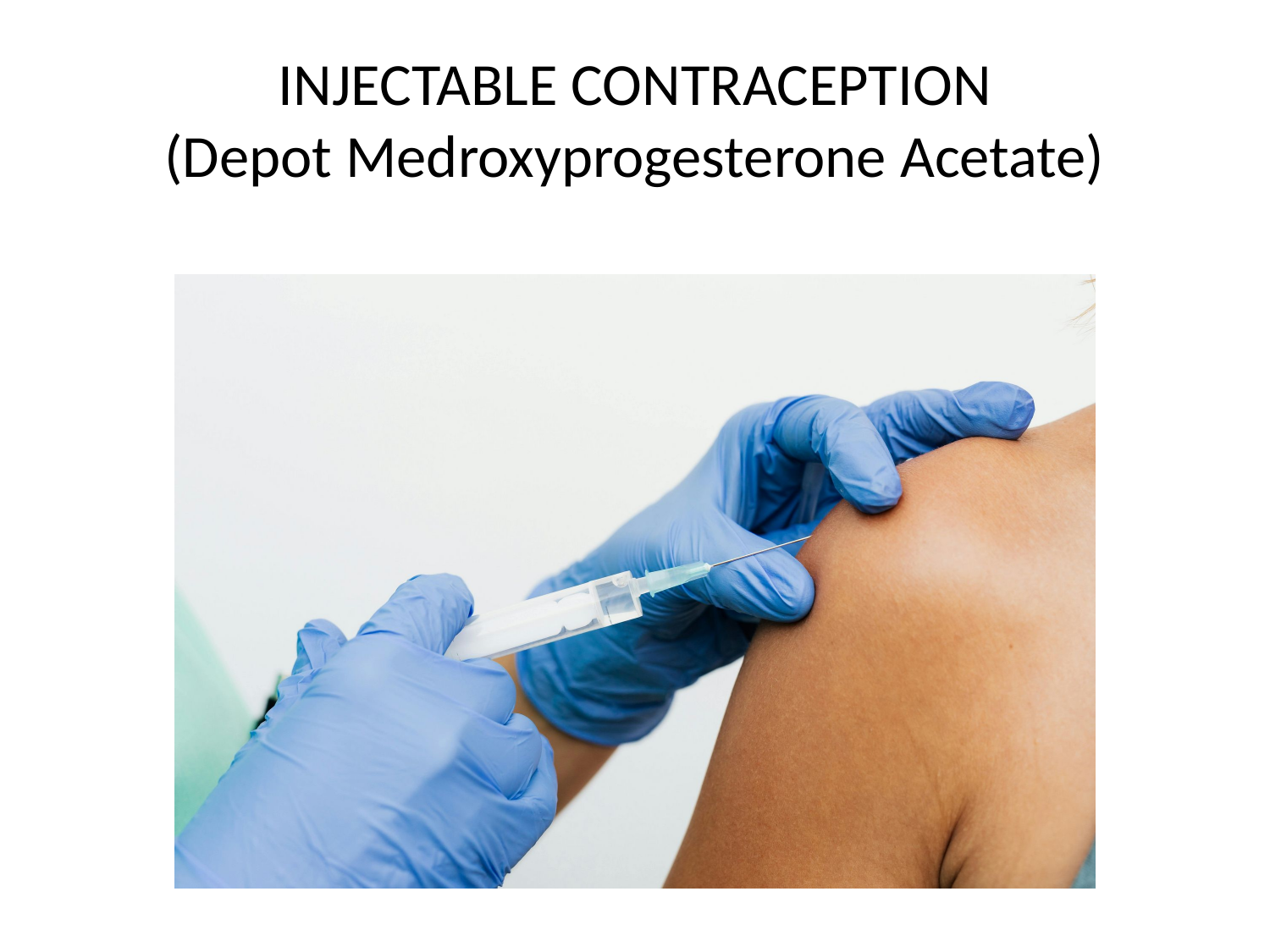

# INJECTABLE CONTRACEPTION (Depot Medroxyprogesterone Acetate)

## Slide 5
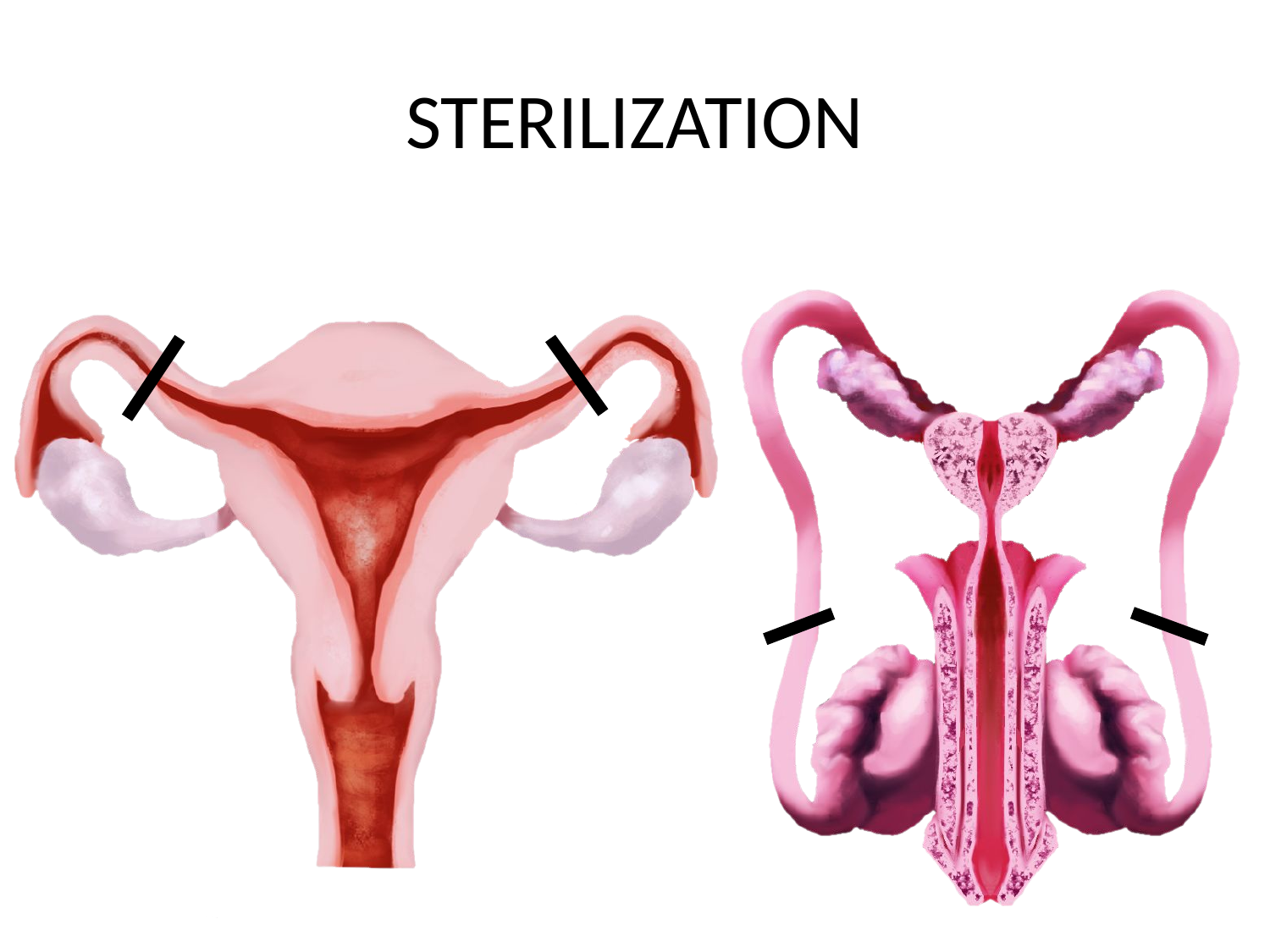

# STERILIZATION

## Slide 6
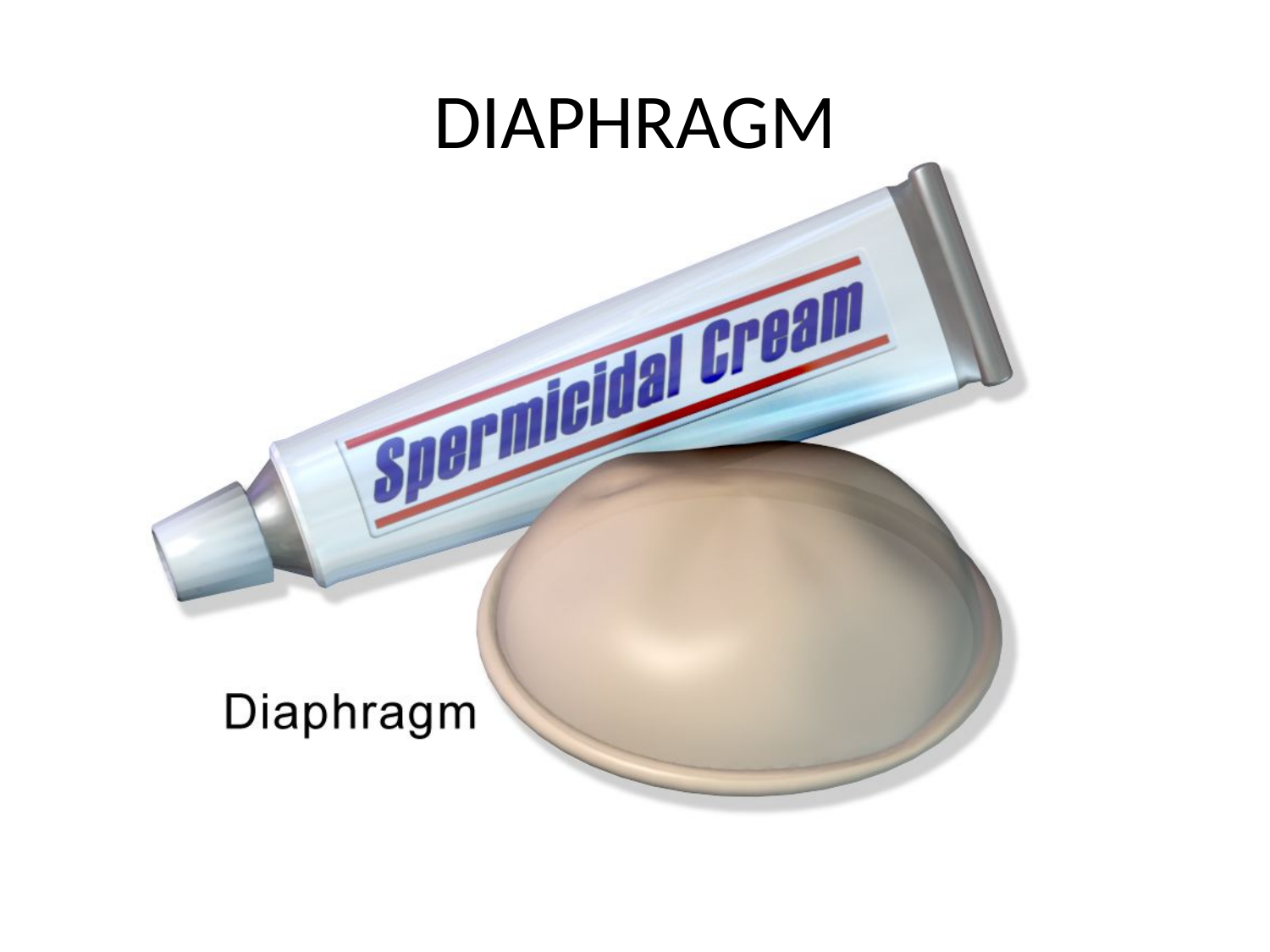

# DIAPHRAGM

## Slide 7
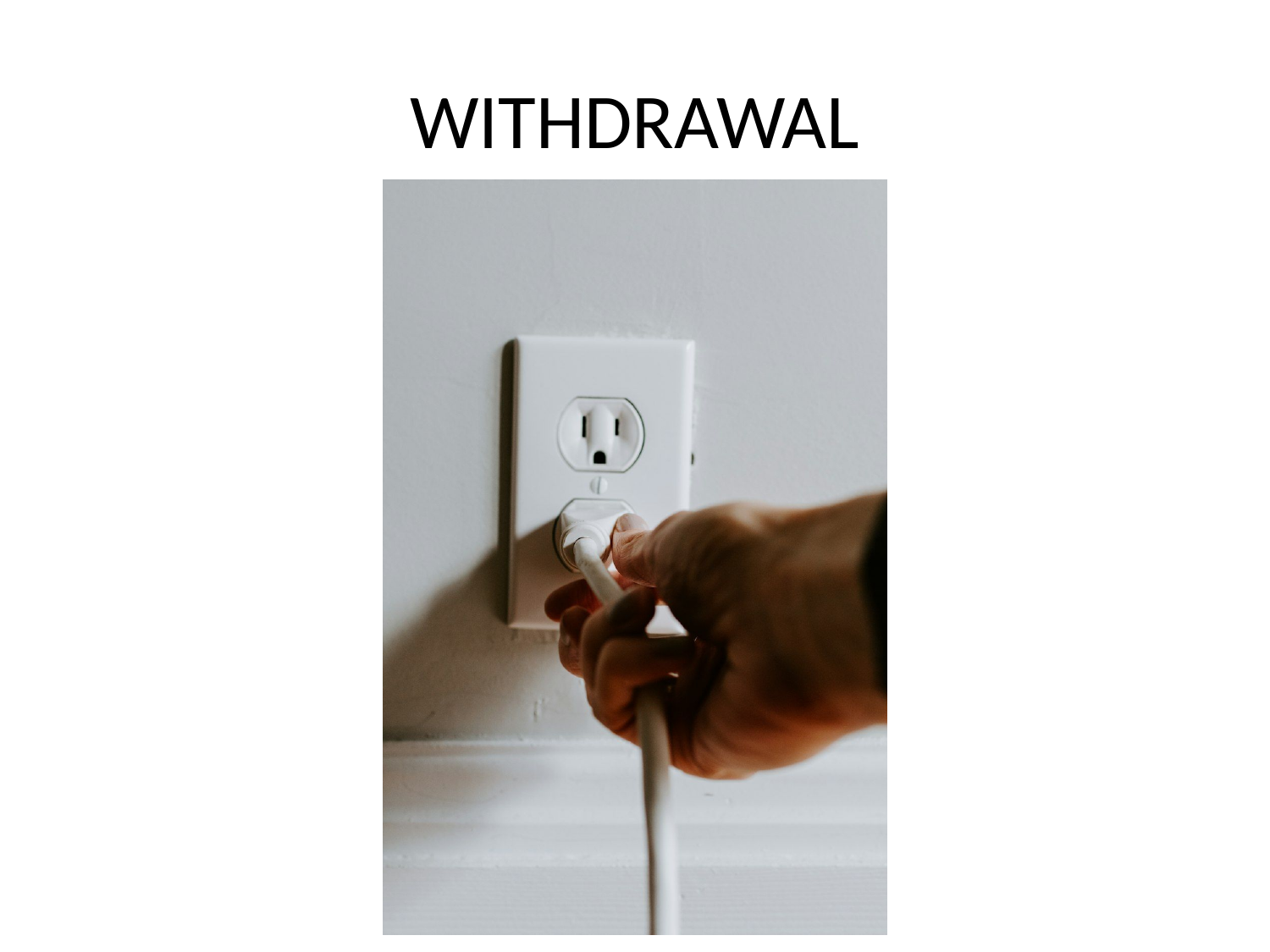

# WITHDRAWAL

## Slide 8
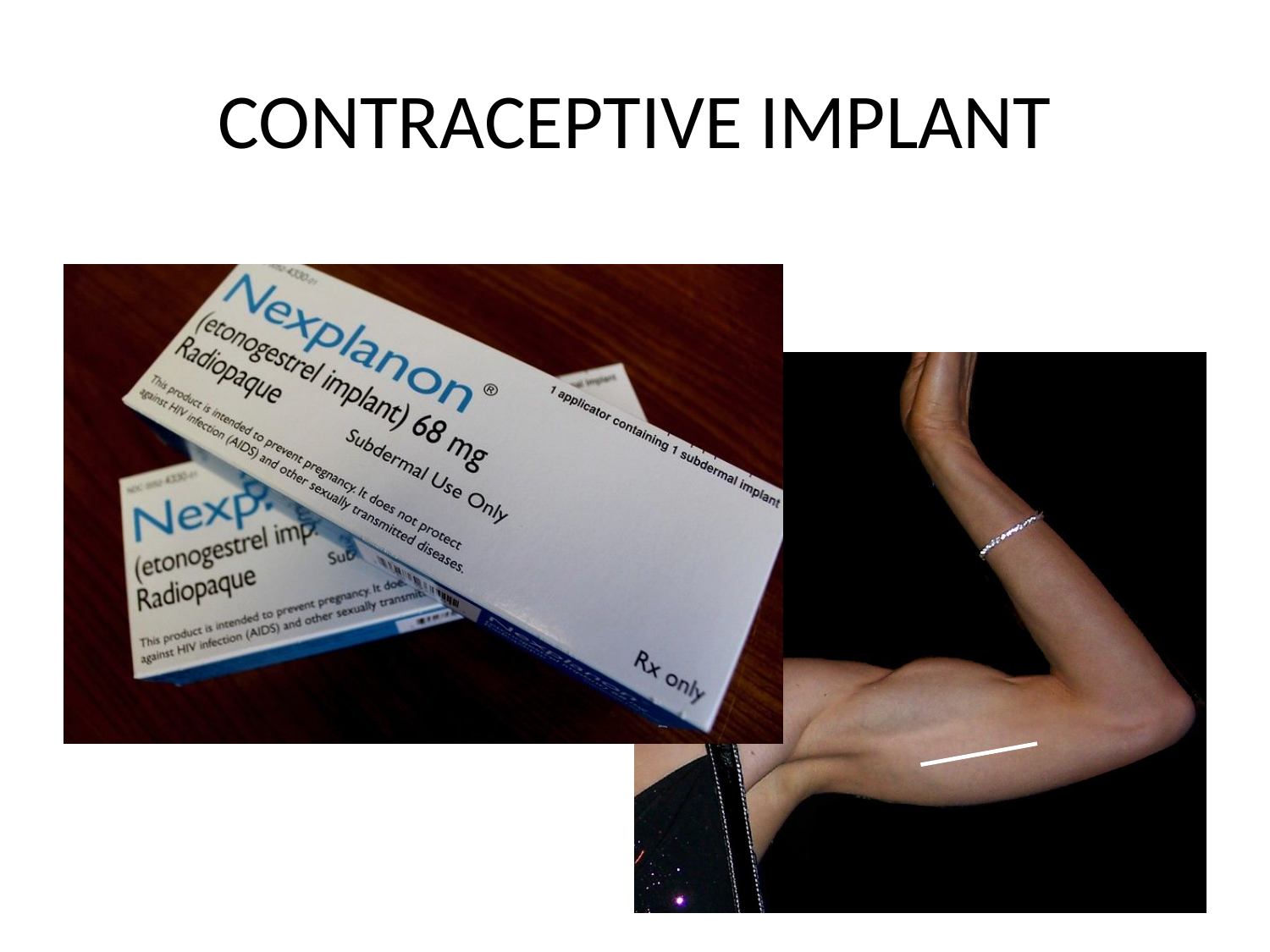

# CONTRACEPTIVE IMPLANT

## Slide 9
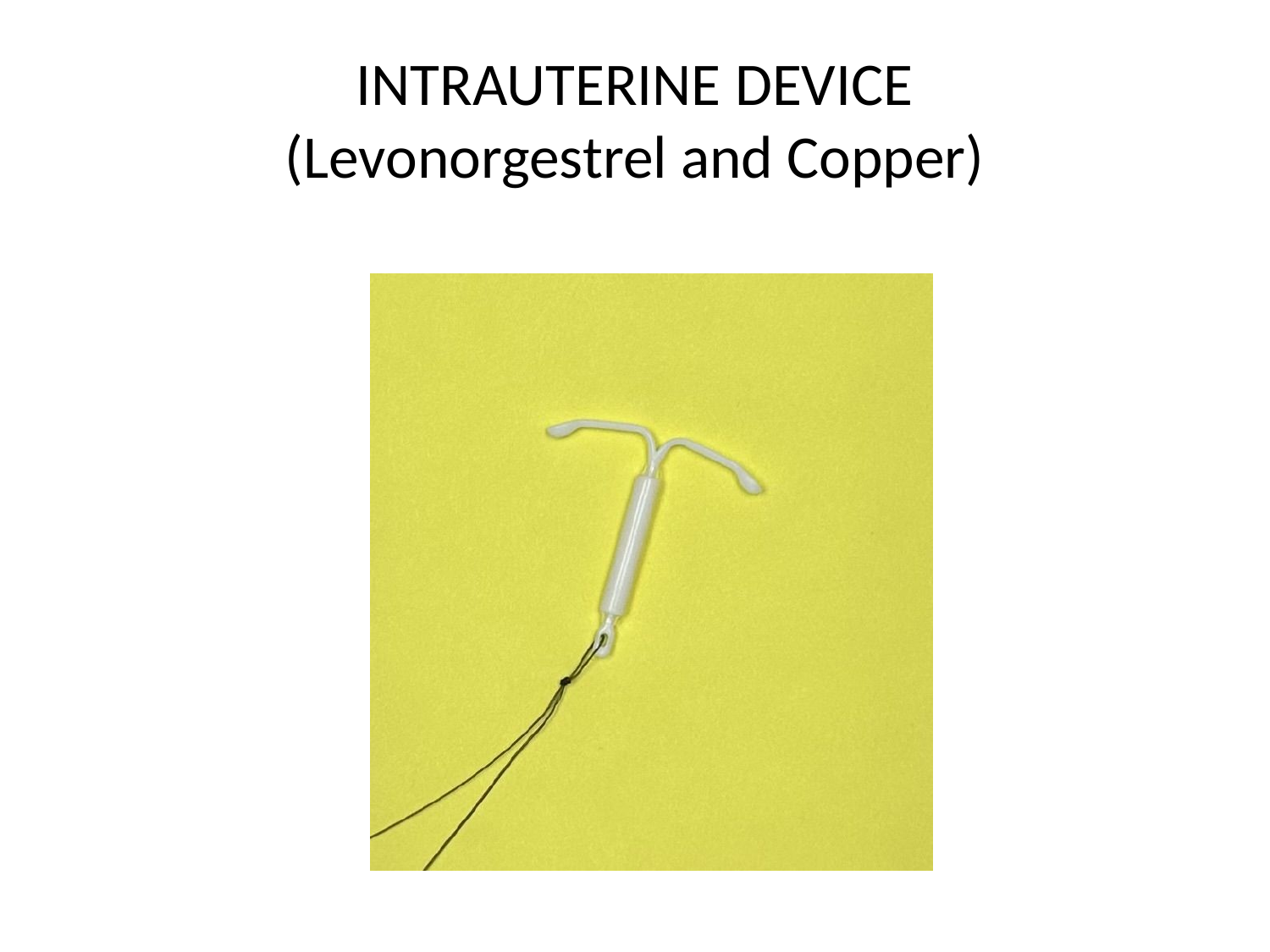

# INTRAUTERINE DEVICE (Levonorgestrel and Copper)

## Slide 10
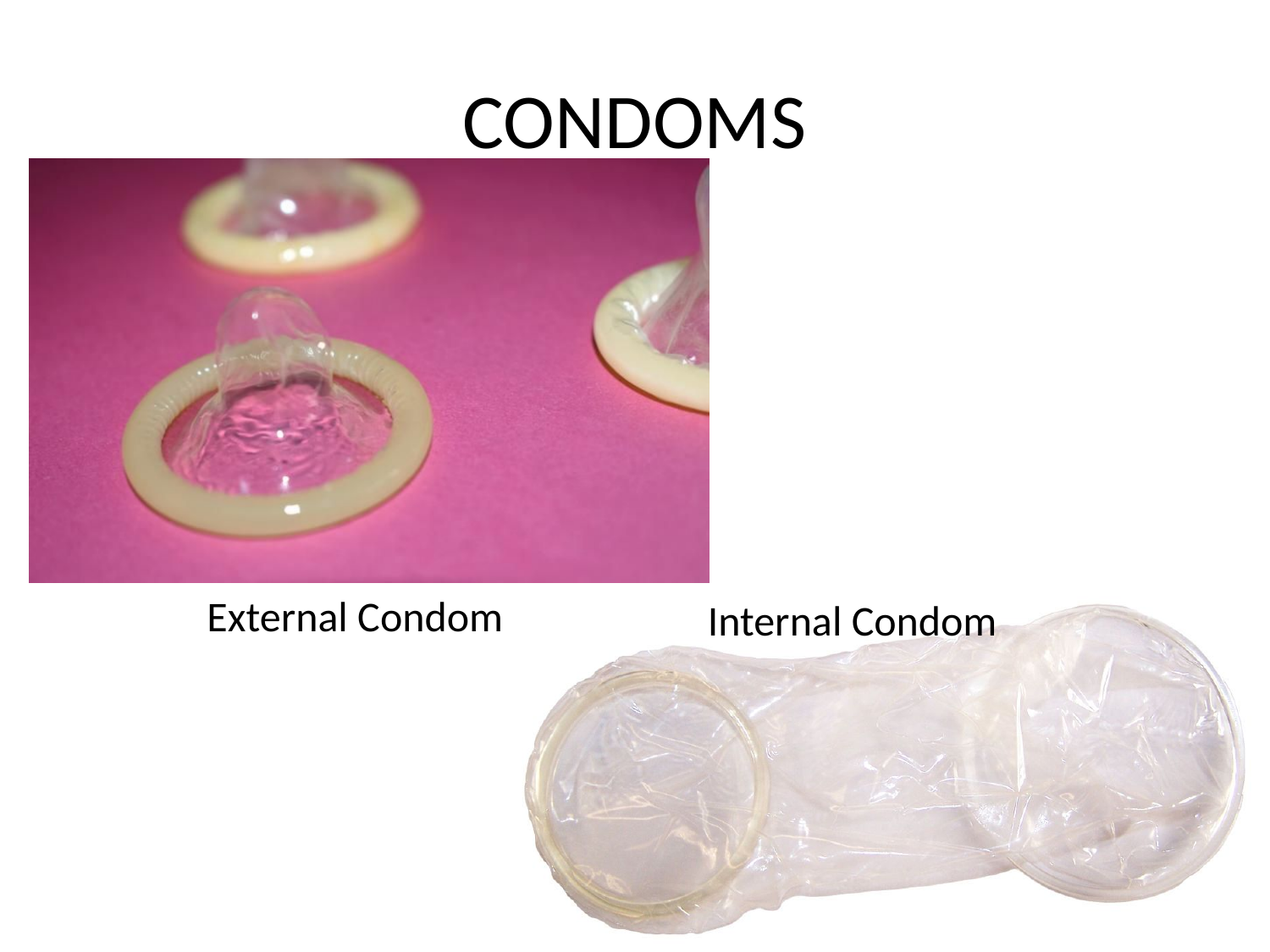

# CONDOMS
External Condom
Internal Condom

## Slide 11
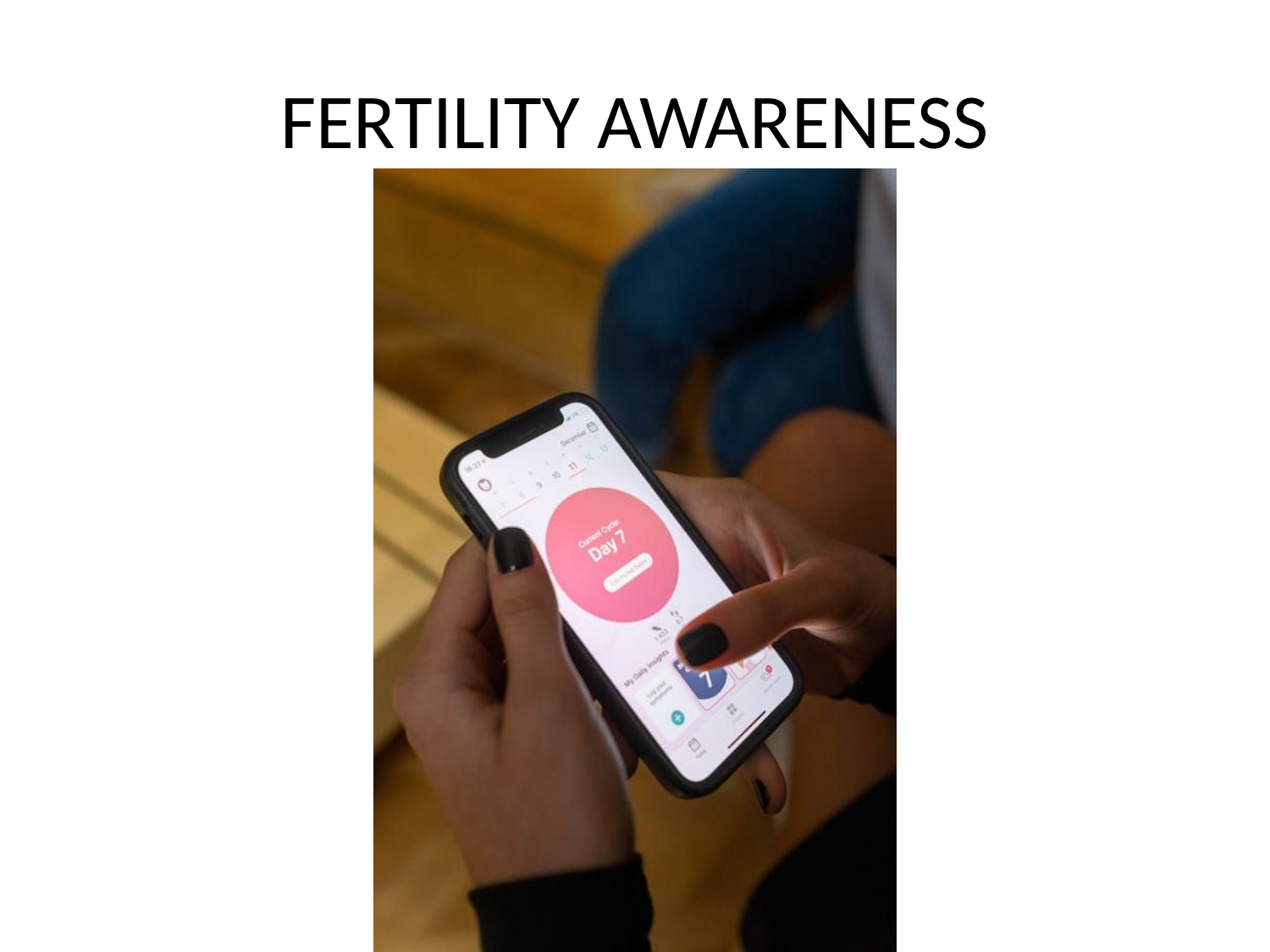

# FERTILITY AWARENESS

## Slide 12
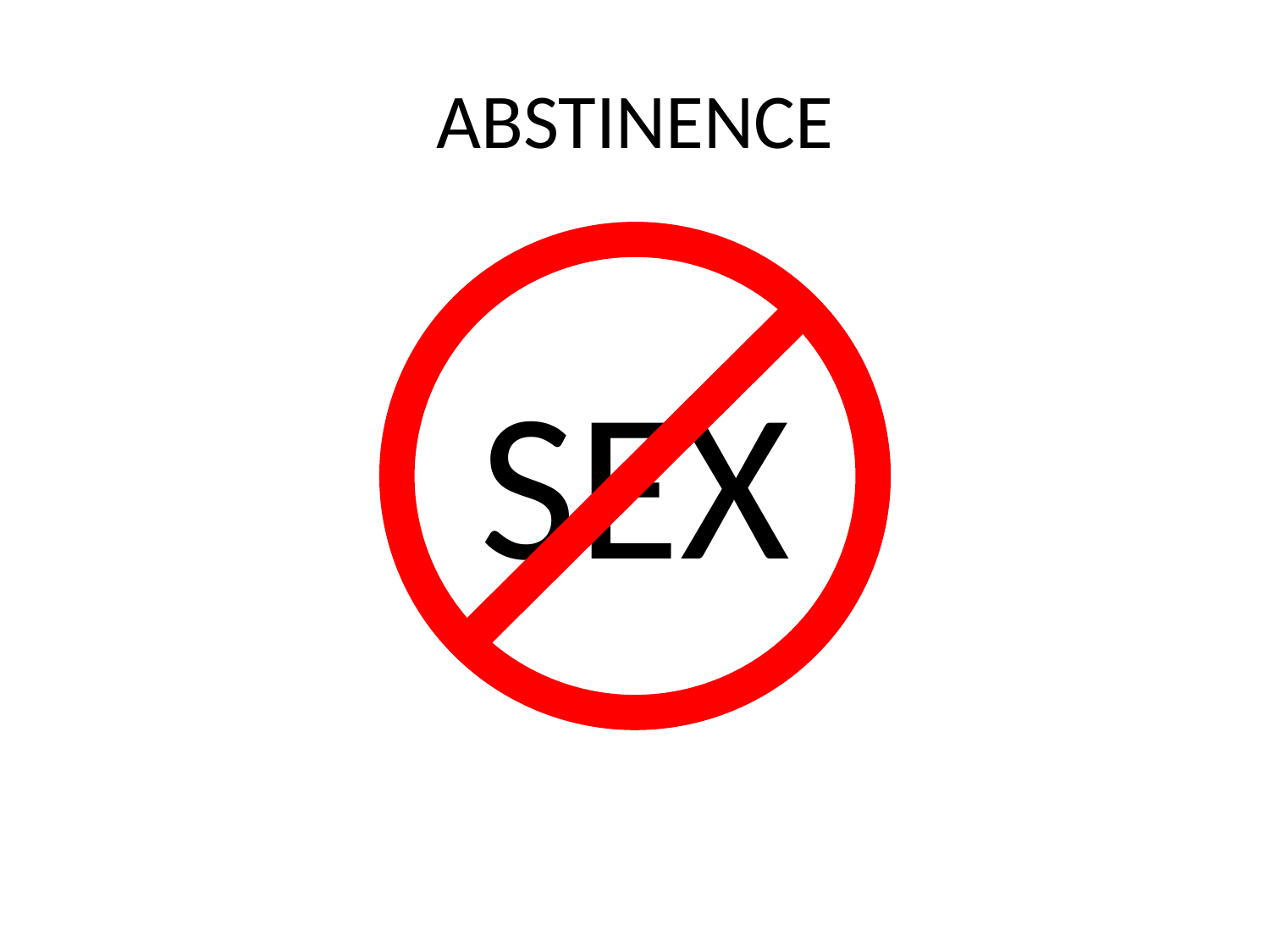

# ABSTINENCE
SEX

## Slide 13
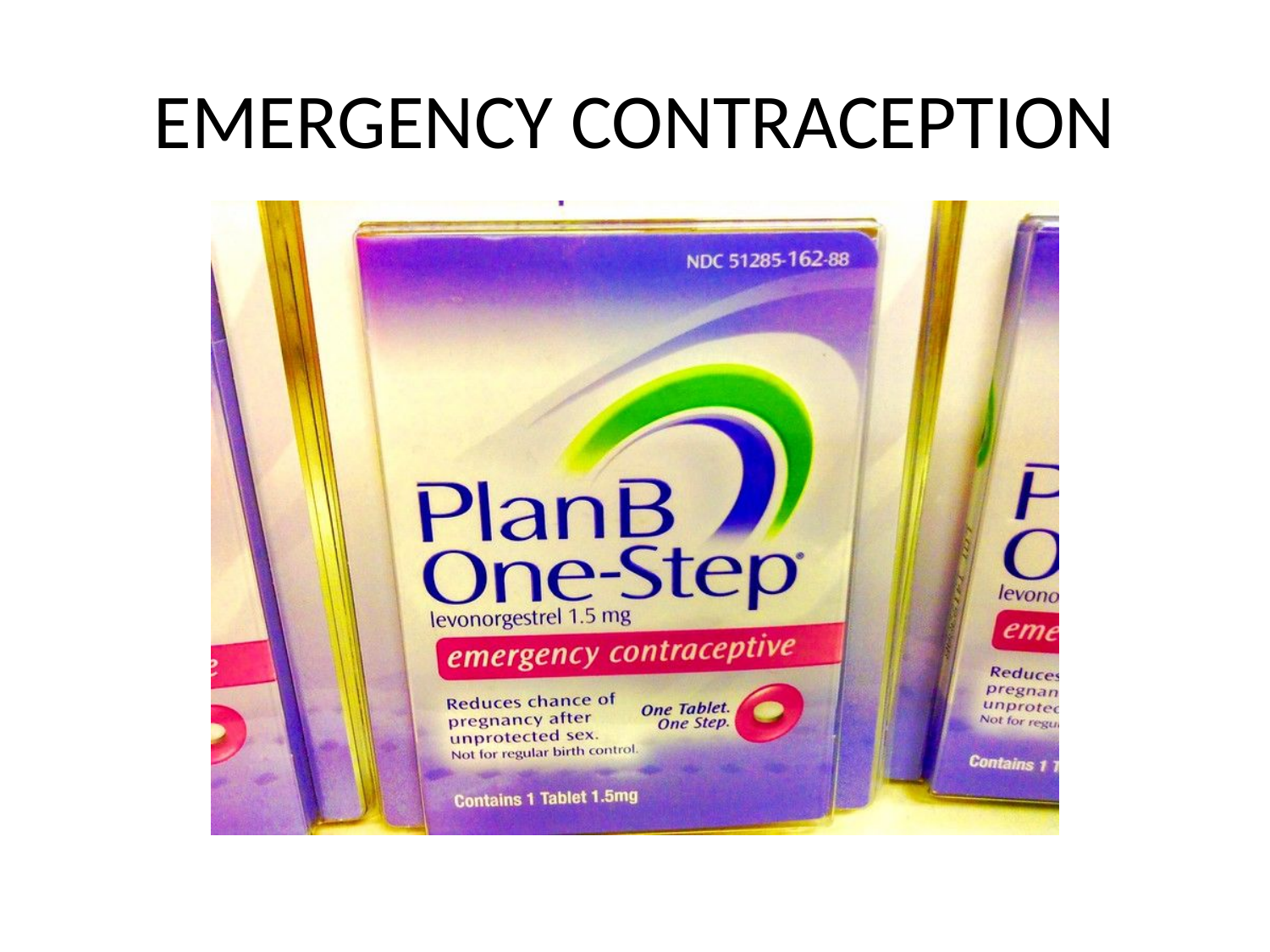

# EMERGENCY CONTRACEPTION
