# Supplementary material for: A Flipped Classroom Case to Introduce OB/GYN Clerkship Students to Contraception, Postpartum Care, and Intimate Partner Violence Screening
Source: MedEdPORTAL. 2025 Apr 9;21:11505. doi: 10.15766/mep_2374-8265.11505 (PMC11978902; doi:10.15766/mep_2374-8265.11505)
Supplement: Supplementary file 1 — Student Prework.docxContraception Cards.pptxPostpartum Slides.pptxFacilitator Guide.docxFacilitator Survey.docxStudent Survey.docx [file mep_2374-8265.11505-s001.zip › C. Postpartum Slides.pptx]

## Slide 1
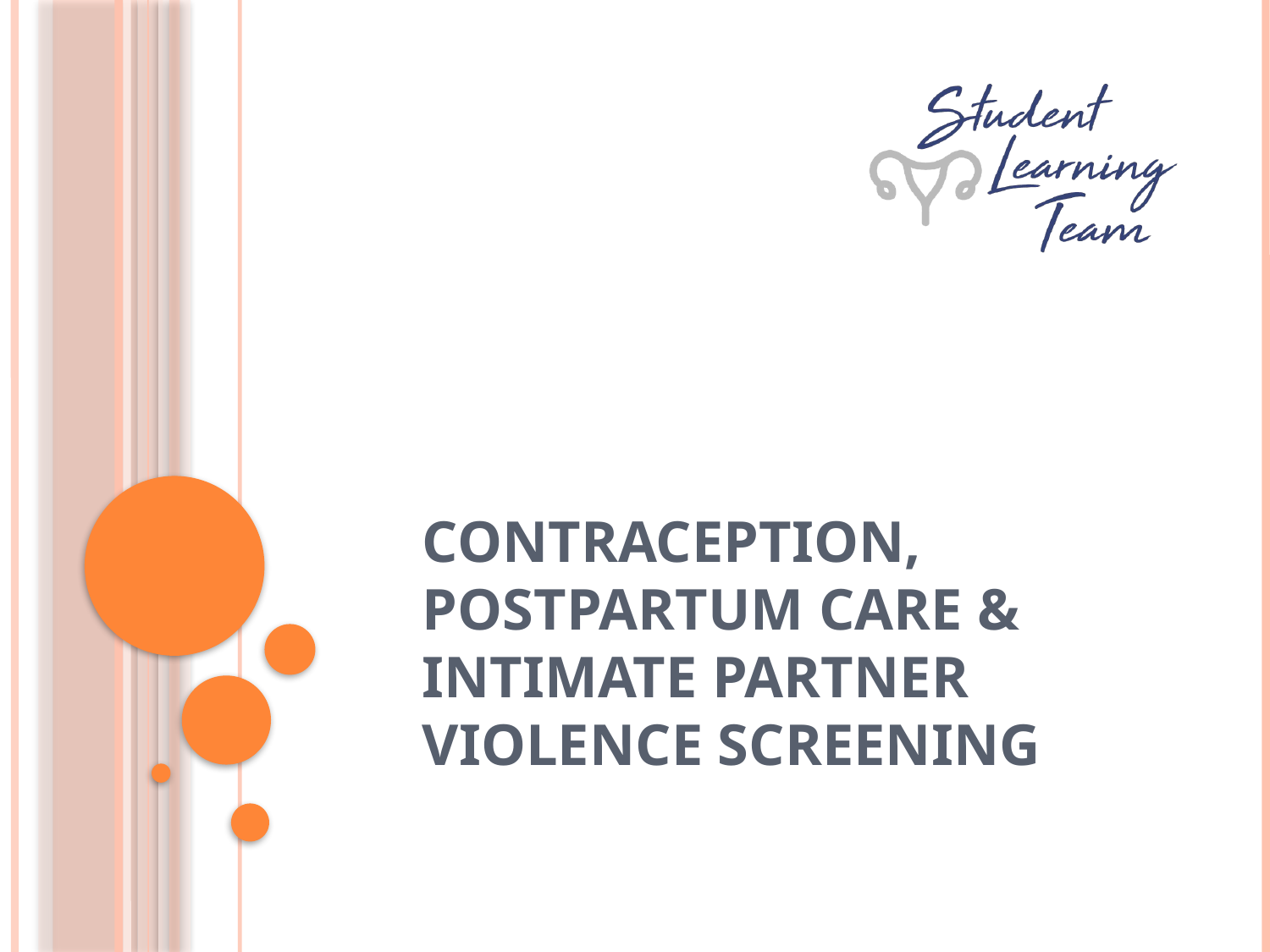

# contraception, postpartum care & intimate partner violence screening

## Slide 2
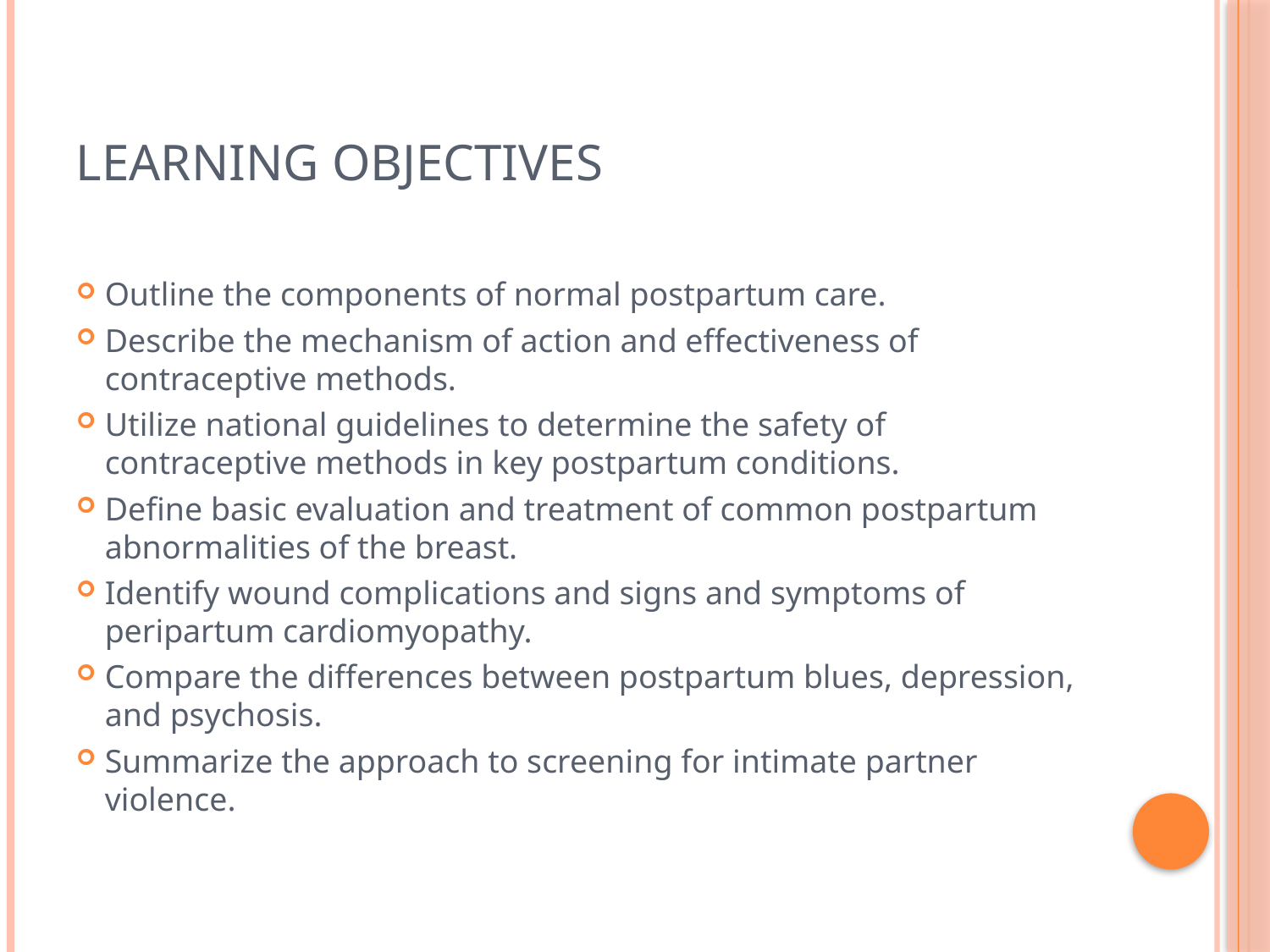

# Learning objectives
Outline the components of normal postpartum care.
Describe the mechanism of action and effectiveness of contraceptive methods.
Utilize national guidelines to determine the safety of contraceptive methods in key postpartum conditions.
Define basic evaluation and treatment of common postpartum abnormalities of the breast.
Identify wound complications and signs and symptoms of peripartum cardiomyopathy.
Compare the differences between postpartum blues, depression, and psychosis.
Summarize the approach to screening for intimate partner violence.

## Slide 3
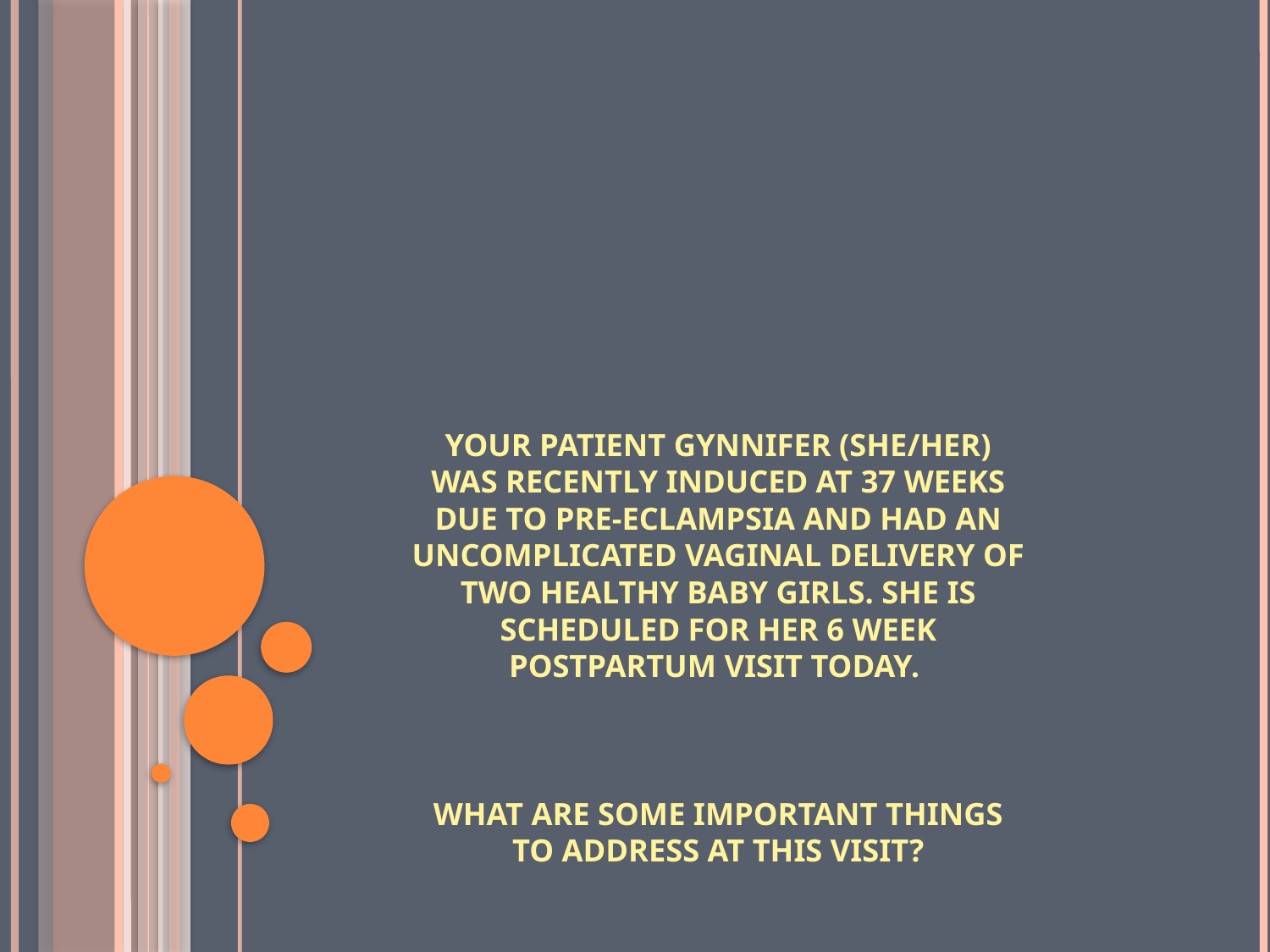

# Your patient Gynnifer (she/her) was recently induced at 37 weeks due to pre-eclampsia and had an uncomplicated vaginal delivery of two healthy baby girls. She is scheduled for her 6 week postpartum visit today. What are some important things to address at this visit?

## Slide 4
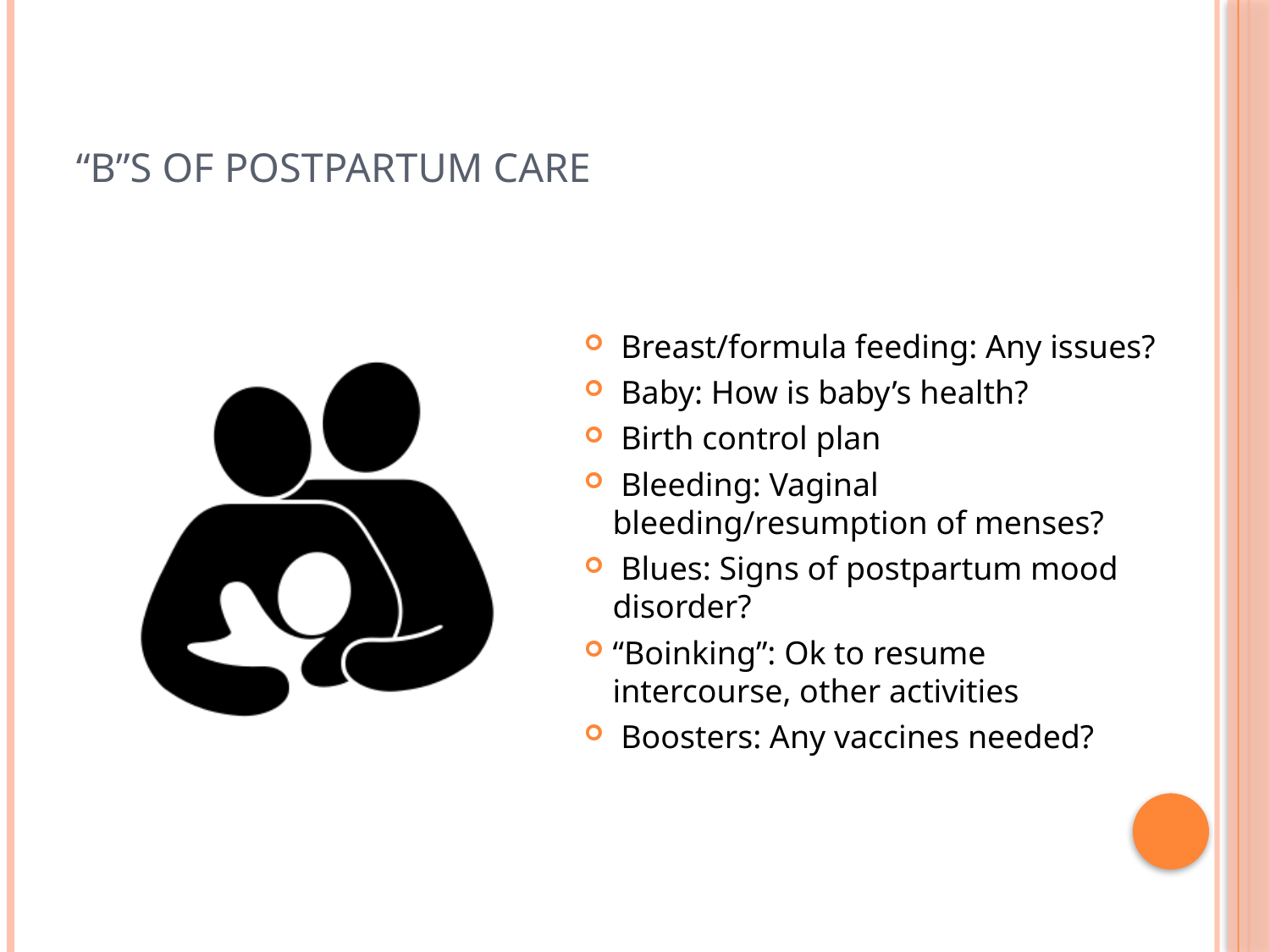

# “B”s of postpartum care
 Breast/formula feeding: Any issues?
 Baby: How is baby’s health?
 Birth control plan
 Bleeding: Vaginal bleeding/resumption of menses?
 Blues: Signs of postpartum mood disorder?
“Boinking”: Ok to resume intercourse, other activities
 Boosters: Any vaccines needed?

## Slide 5
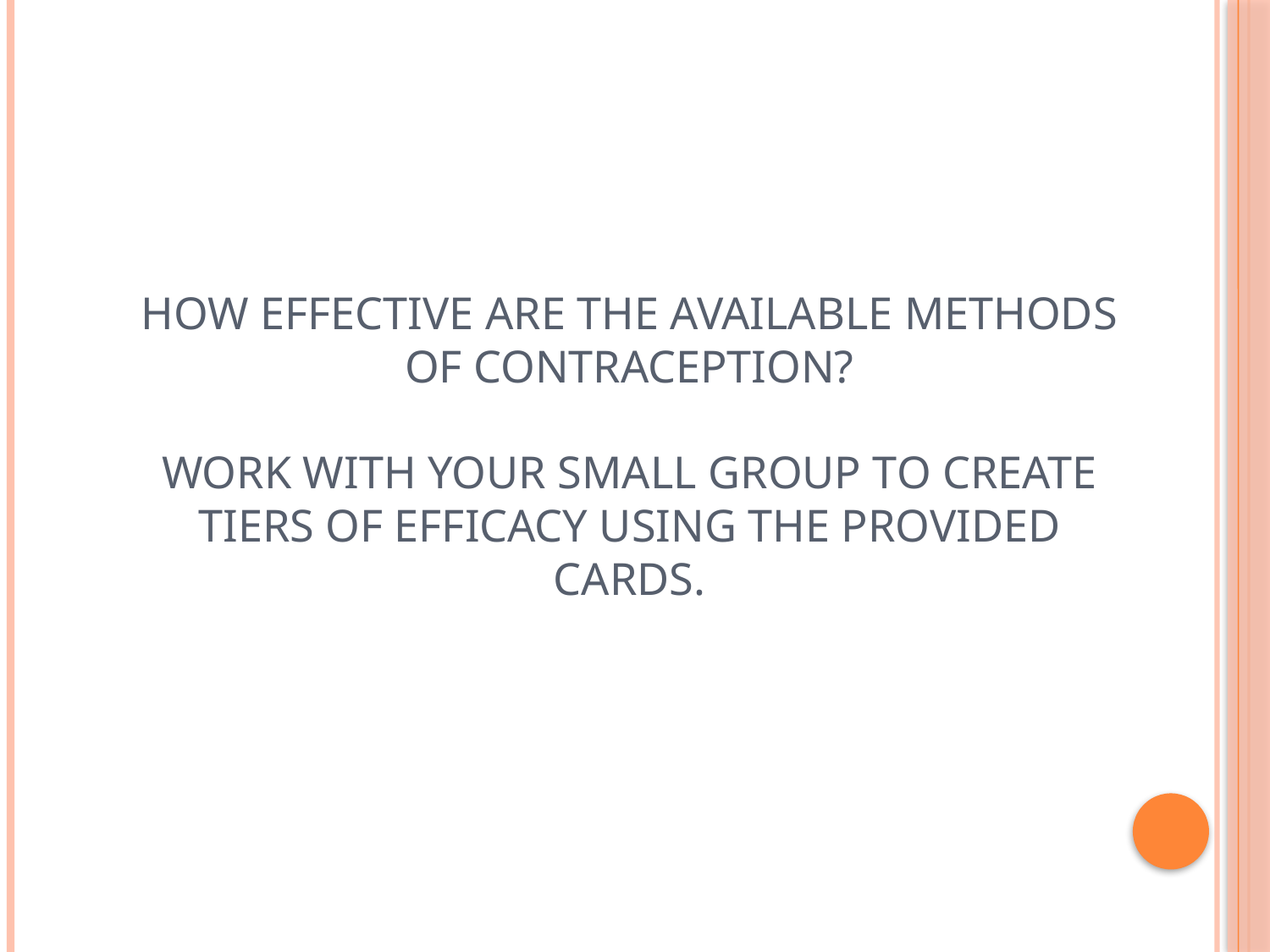

# How effective are the available methods of contraception? Work with your small group to create tiers of efficacy using the provided cards.

## Slide 6
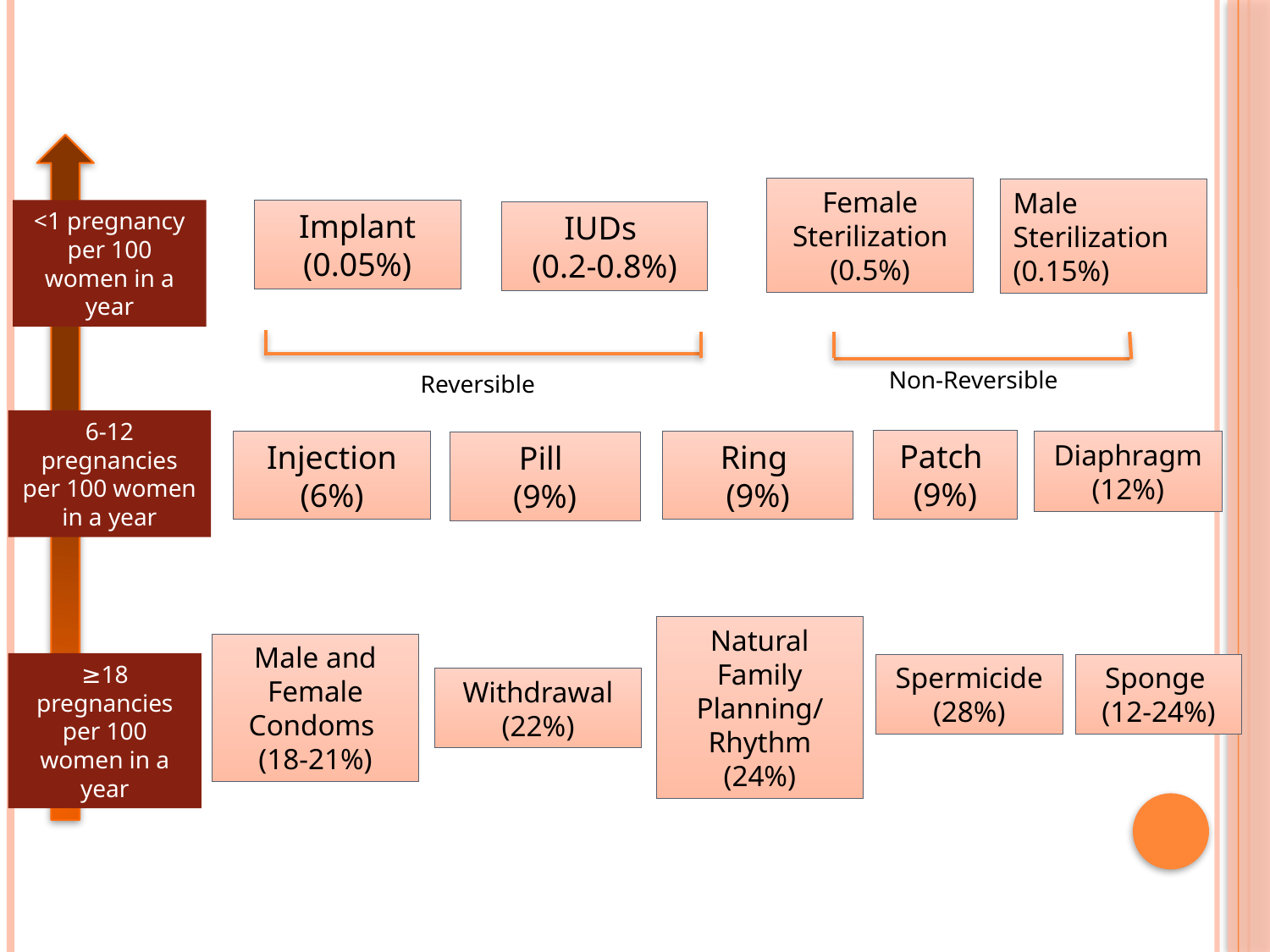

Female Sterilization (0.5%)
Male Sterilization (0.15%)
<1 pregnancy per 100 women in a year
Implant (0.05%)
IUDs
(0.2-0.8%)
Non-Reversible
Reversible
6-12 pregnancies per 100 women in a year
Patch
(9%)
Injection (6%)
Ring
(9%)
Diaphragm (12%)
Pill
(9%)
Natural Family Planning/
Rhythm (24%)
Male and Female Condoms
(18-21%)
≥18 pregnancies per 100 women in a year
Spermicide (28%)
Sponge
(12-24%)
Withdrawal (22%)

## Slide 7
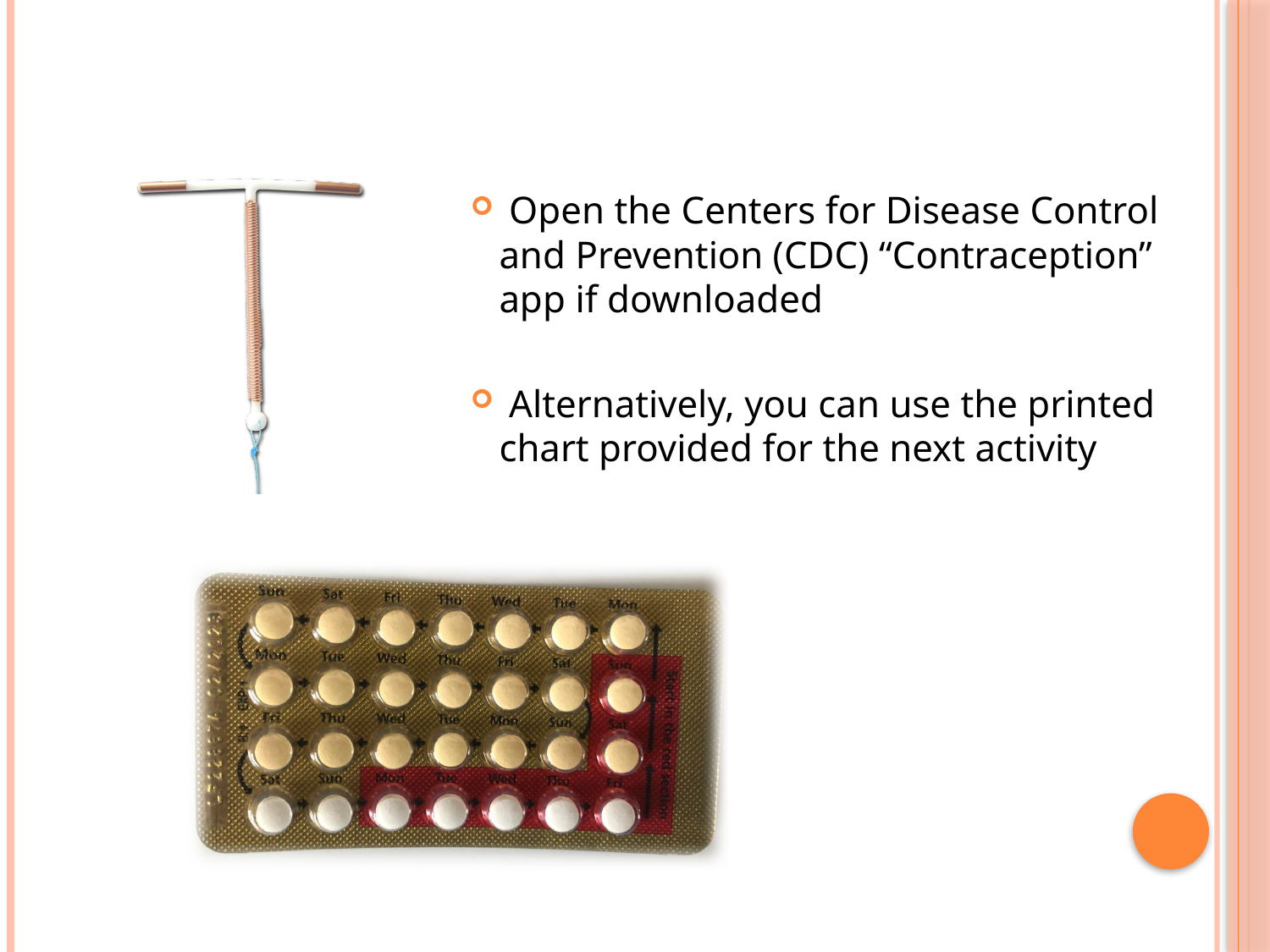

Open the Centers for Disease Control and Prevention (CDC) “Contraception” app if downloaded
 Alternatively, you can use the printed chart provided for the next activity

## Slide 8
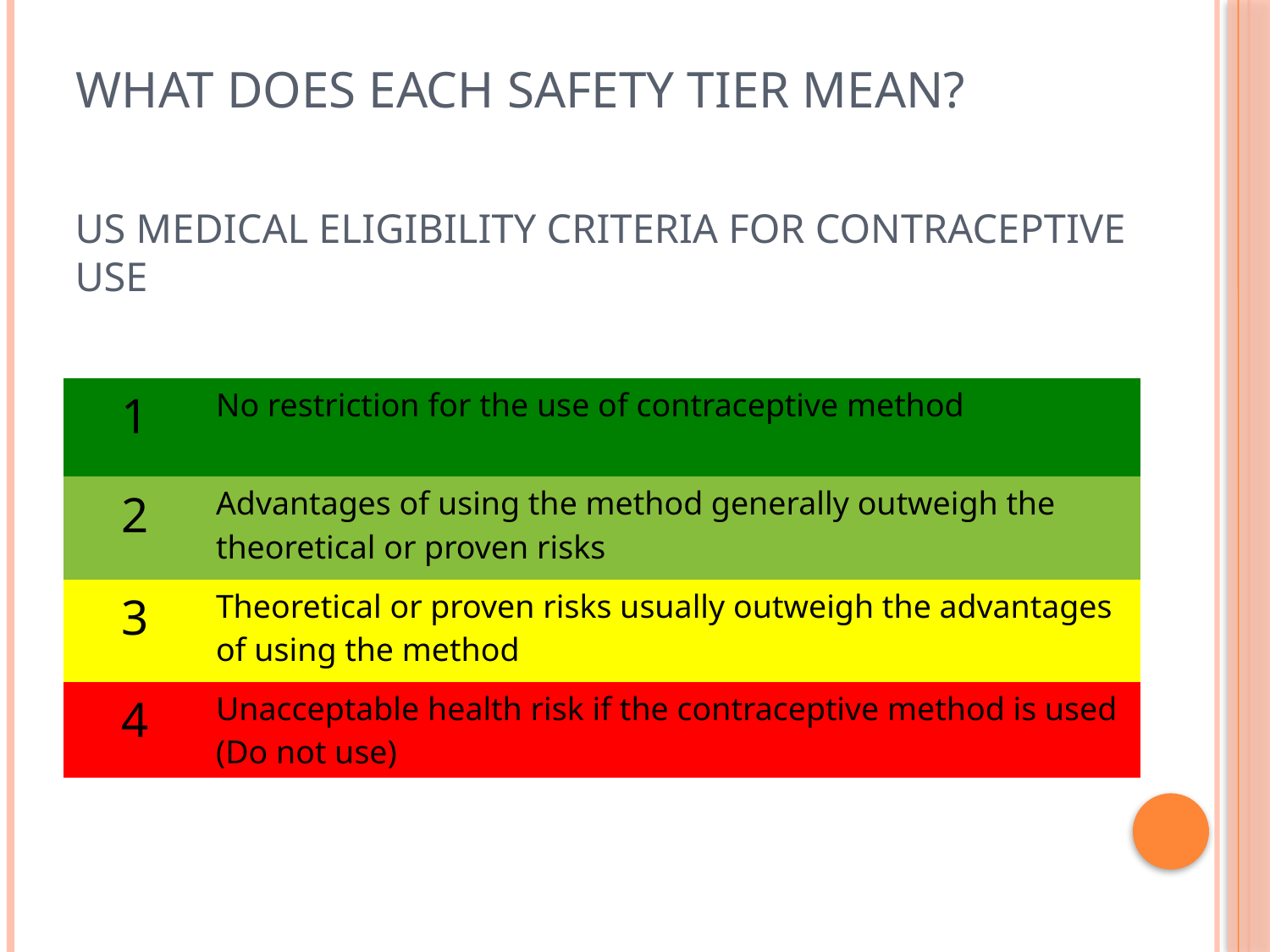

What does each safety tier mean?
# US Medical Eligibility Criteria for Contraceptive Use
| 1 | No restriction for the use of contraceptive method |
| --- | --- |
| 2 | Advantages of using the method generally outweigh the theoretical or proven risks |
| 3 | Theoretical or proven risks usually outweigh the advantages of using the method |
| 4 | Unacceptable health risk if the contraceptive method is used (Do not use) |

## Slide 9
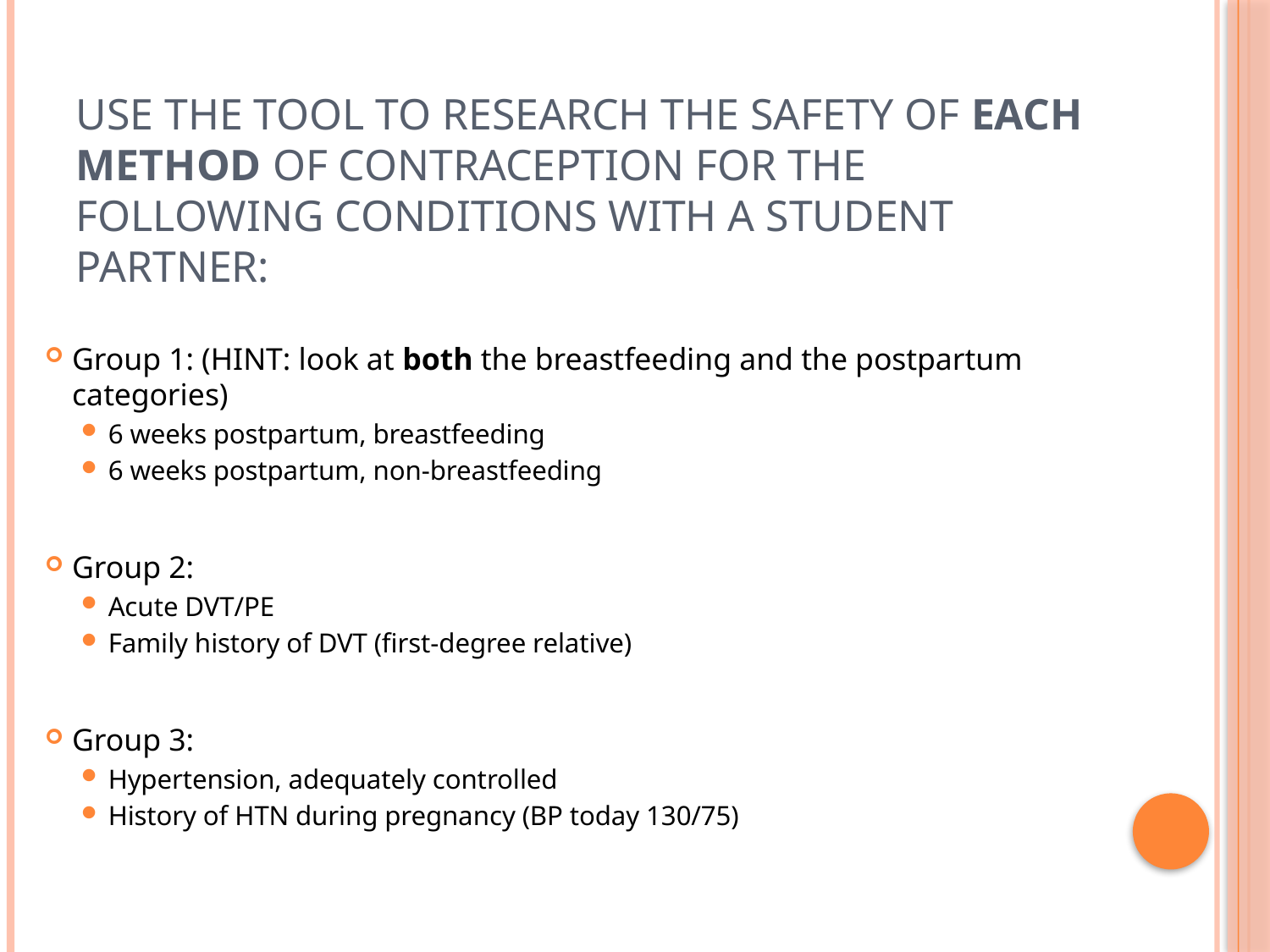

# Use the tool to research the safety of each method of contraception for the following conditions with a student partner:
Group 1: (HINT: look at both the breastfeeding and the postpartum categories)
6 weeks postpartum, breastfeeding
6 weeks postpartum, non-breastfeeding
Group 2:
Acute DVT/PE
Family history of DVT (first-degree relative)
Group 3:
Hypertension, adequately controlled
History of HTN during pregnancy (BP today 130/75)

## Slide 10
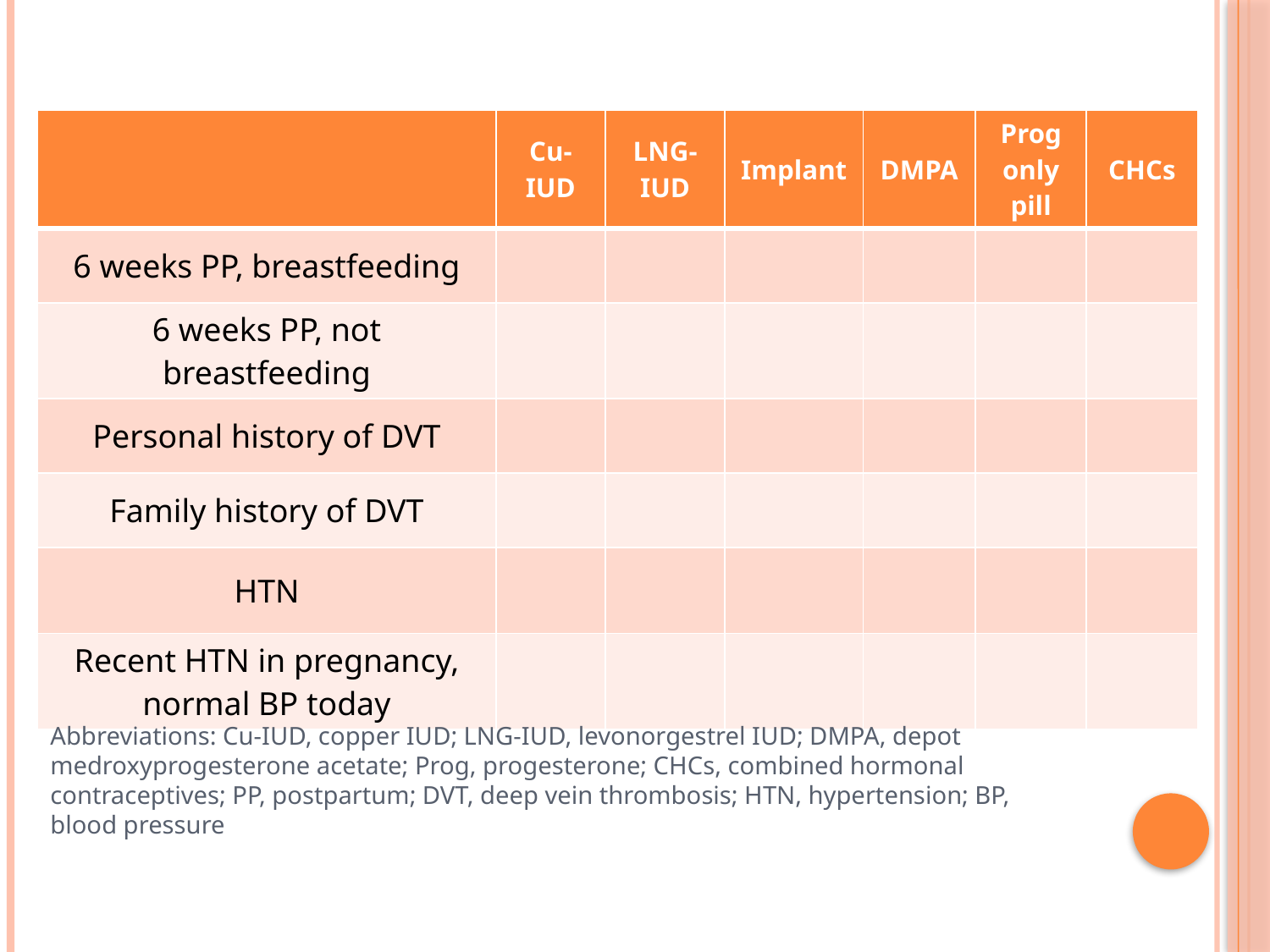

| | Cu-IUD | LNG-IUD | Implant | DMPA | Prog only pill | CHCs |
| --- | --- | --- | --- | --- | --- | --- |
| 6 weeks PP, breastfeeding | | | | | | |
| 6 weeks PP, not breastfeeding | | | | | | |
| Personal history of DVT | | | | | | |
| Family history of DVT | | | | | | |
| HTN | | | | | | |
| Recent HTN in pregnancy, normal BP today | | | | | | |
Abbreviations: Cu-IUD, copper IUD; LNG-IUD, levonorgestrel IUD; DMPA, depot medroxyprogesterone acetate; Prog, progesterone; CHCs, combined hormonal contraceptives; PP, postpartum; DVT, deep vein thrombosis; HTN, hypertension; BP, blood pressure

## Slide 11
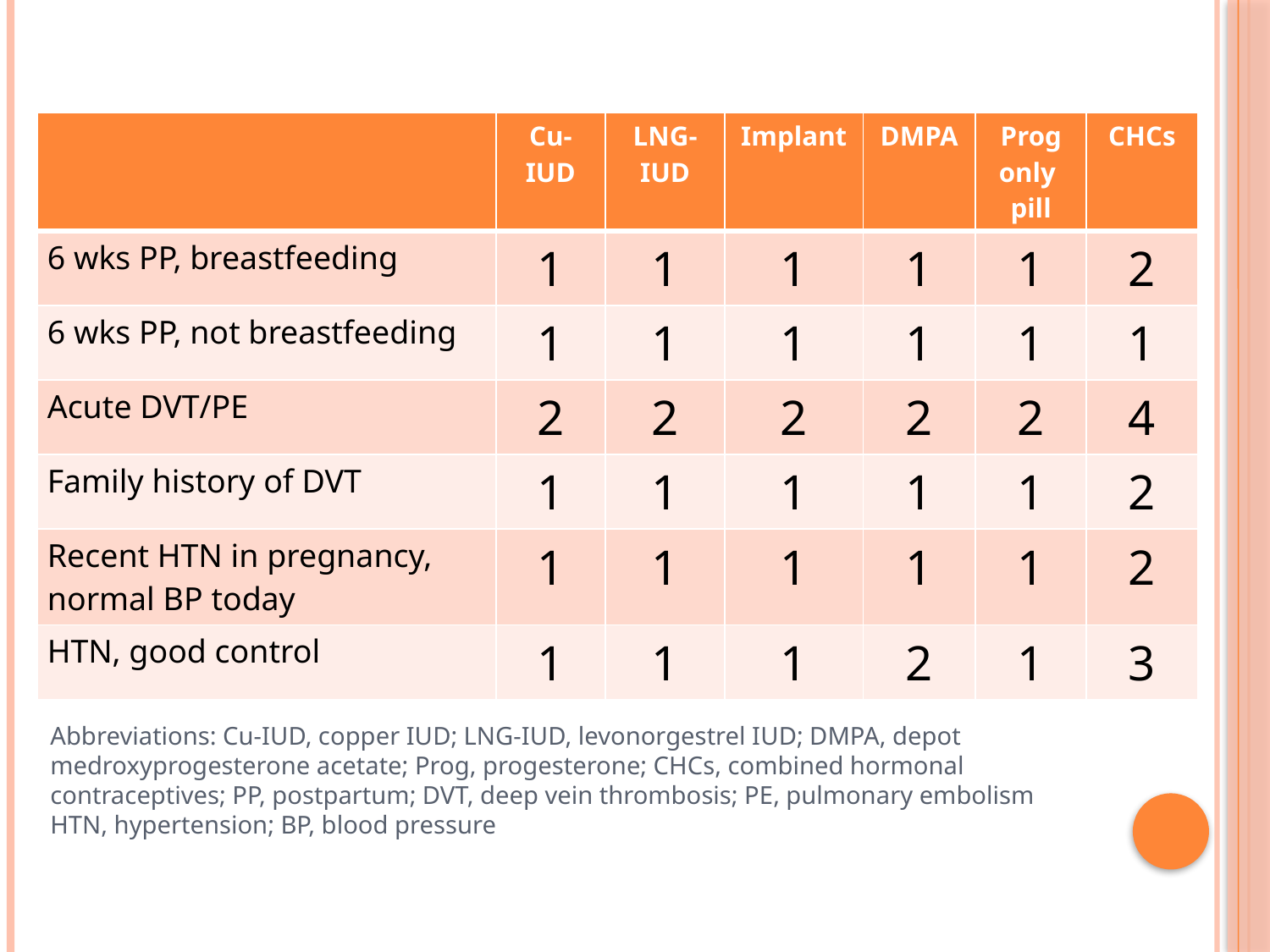

| | Cu-IUD | LNG-IUD | Implant | DMPA | Prog only pill | CHCs |
| --- | --- | --- | --- | --- | --- | --- |
| 6 wks PP, breastfeeding | 1 | 1 | 1 | 1 | 1 | 2 |
| 6 wks PP, not breastfeeding | 1 | 1 | 1 | 1 | 1 | 1 |
| Acute DVT/PE | 2 | 2 | 2 | 2 | 2 | 4 |
| Family history of DVT | 1 | 1 | 1 | 1 | 1 | 2 |
| Recent HTN in pregnancy, normal BP today | 1 | 1 | 1 | 1 | 1 | 2 |
| HTN, good control | 1 | 1 | 1 | 2 | 1 | 3 |
Abbreviations: Cu-IUD, copper IUD; LNG-IUD, levonorgestrel IUD; DMPA, depot medroxyprogesterone acetate; Prog, progesterone; CHCs, combined hormonal contraceptives; PP, postpartum; DVT, deep vein thrombosis; PE, pulmonary embolism HTN, hypertension; BP, blood pressure

## Slide 12
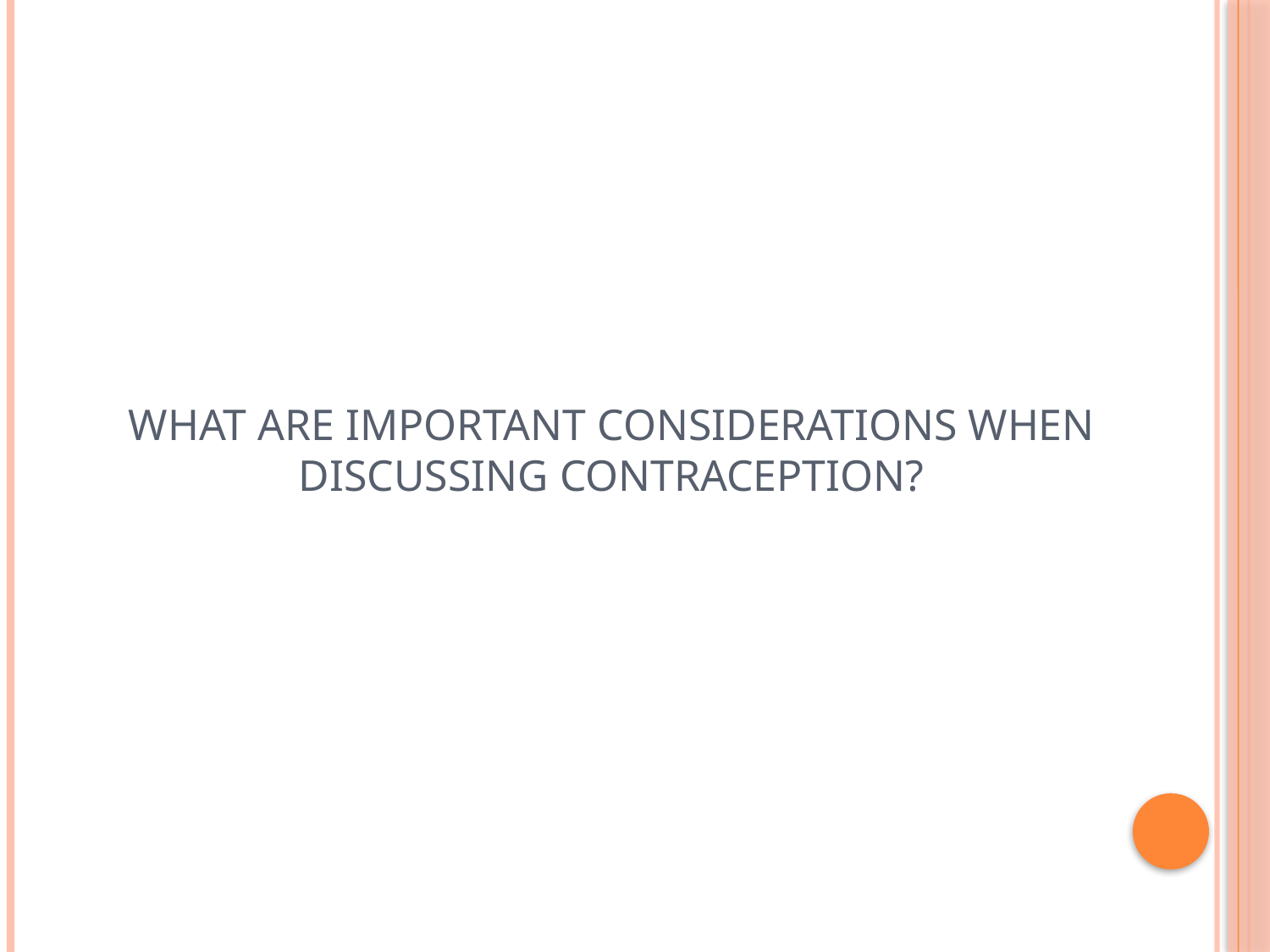

# What are important considerations when discussing contraception?

## Slide 13
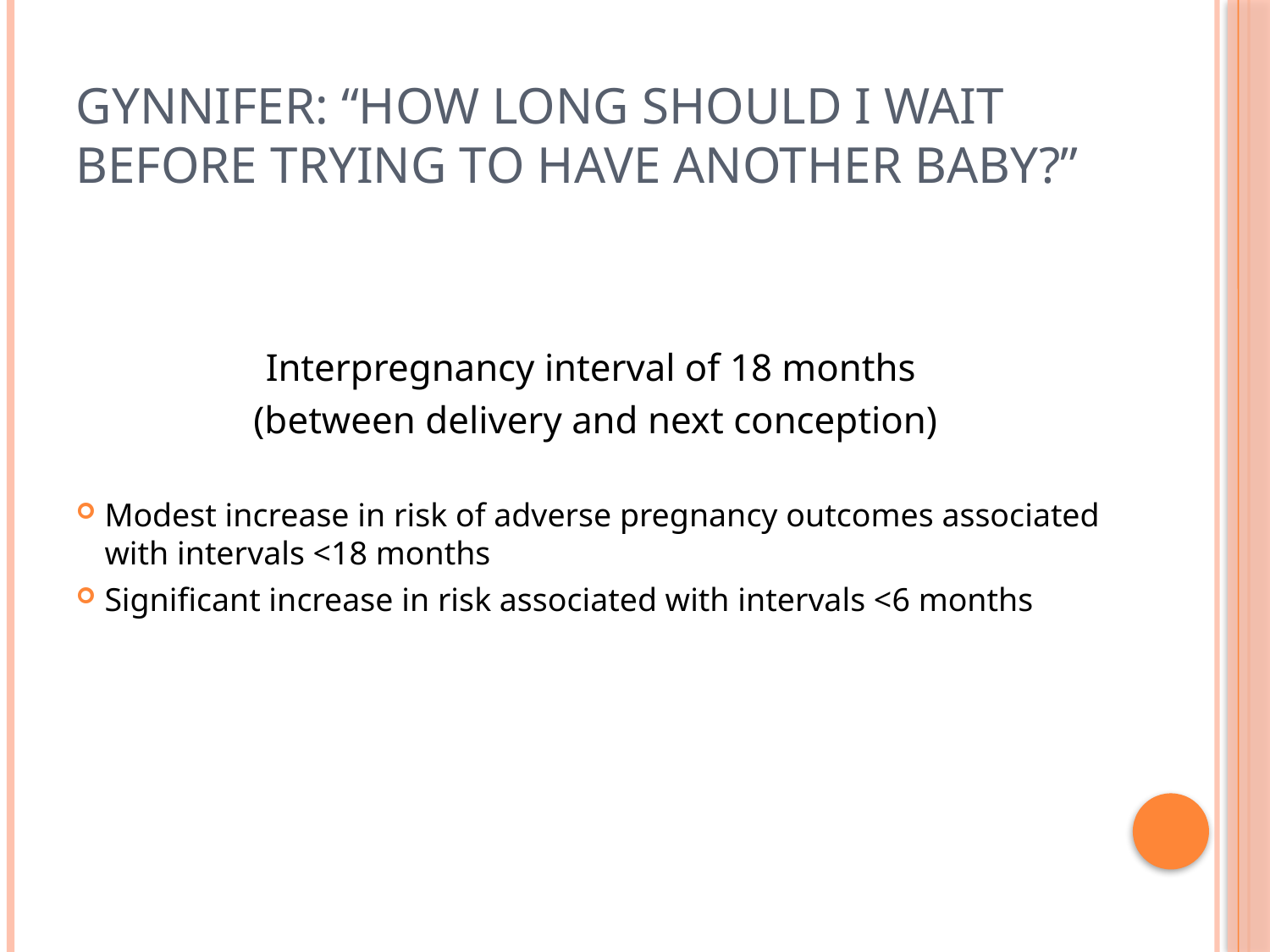

# Gynnifer: “How long should I wait before trying to have another baby?”
Interpregnancy interval of 18 months
(between delivery and next conception)
Modest increase in risk of adverse pregnancy outcomes associated with intervals <18 months
Significant increase in risk associated with intervals <6 months

## Slide 14
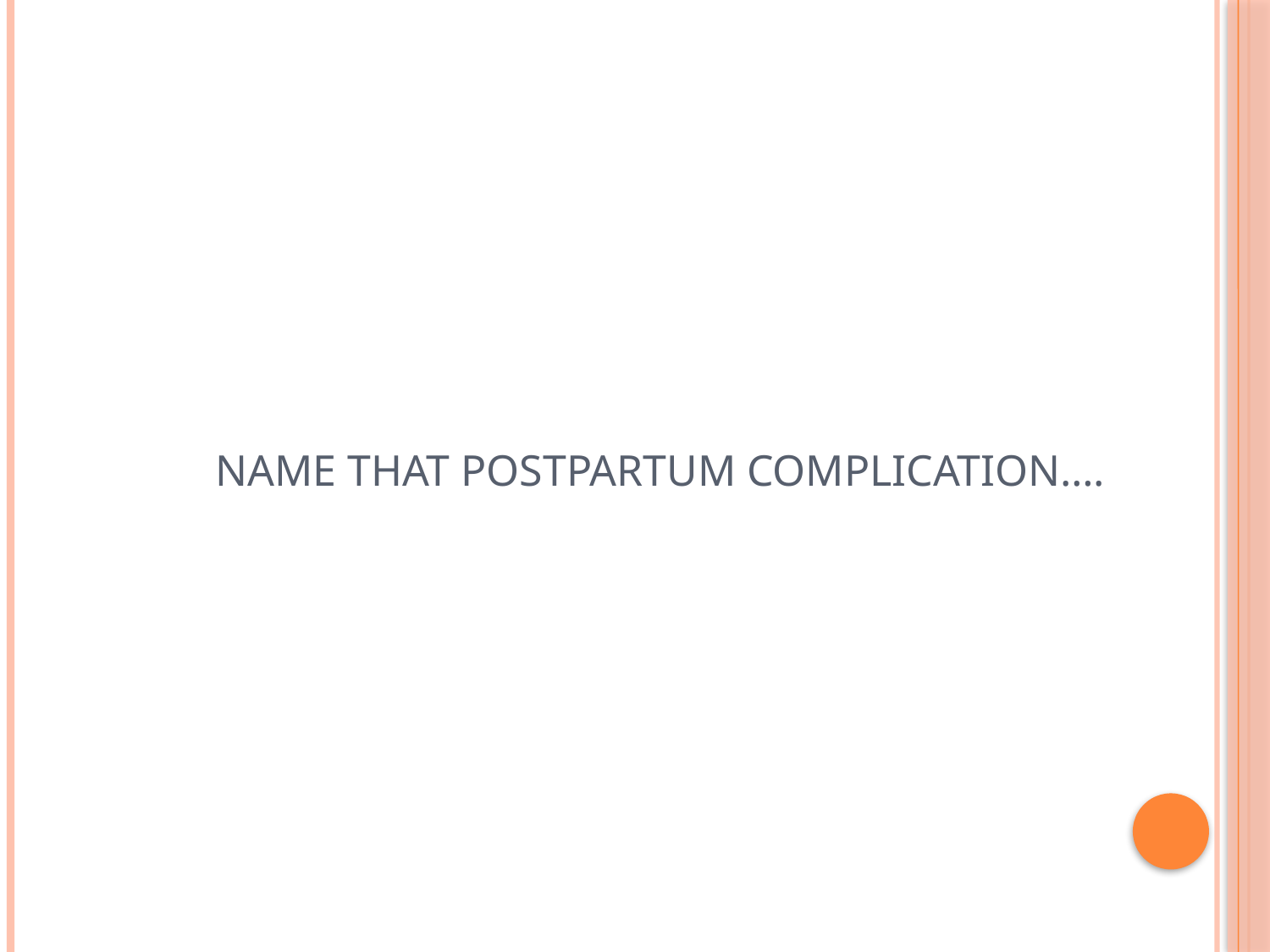

# Name that postpartum complication….

## Slide 15
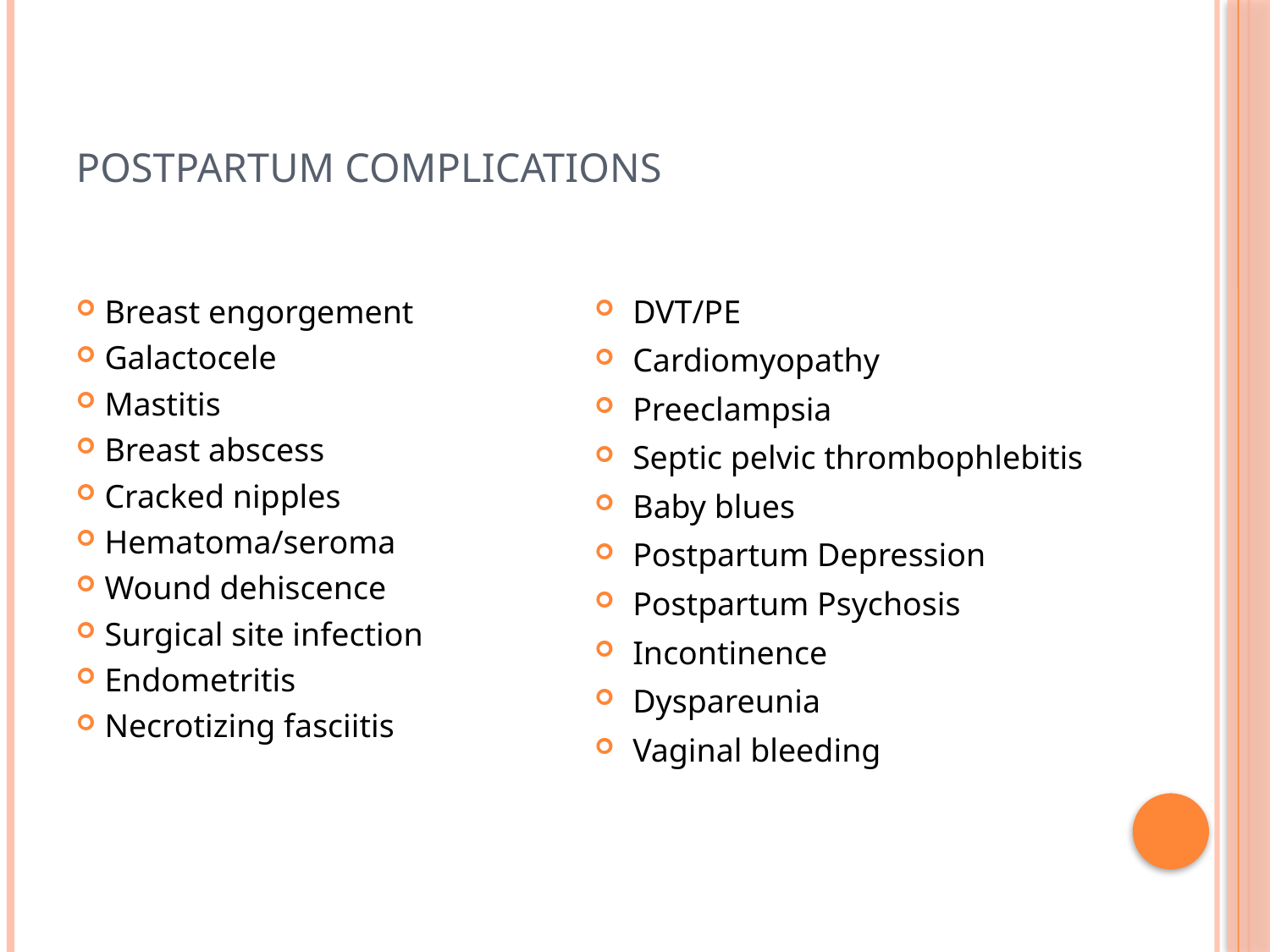

# Postpartum Complications
Breast engorgement
Galactocele
Mastitis
Breast abscess
Cracked nipples
Hematoma/seroma
Wound dehiscence
Surgical site infection
Endometritis
Necrotizing fasciitis
DVT/PE
Cardiomyopathy
Preeclampsia
Septic pelvic thrombophlebitis
Baby blues
Postpartum Depression
Postpartum Psychosis
Incontinence
Dyspareunia
Vaginal bleeding

## Slide 16
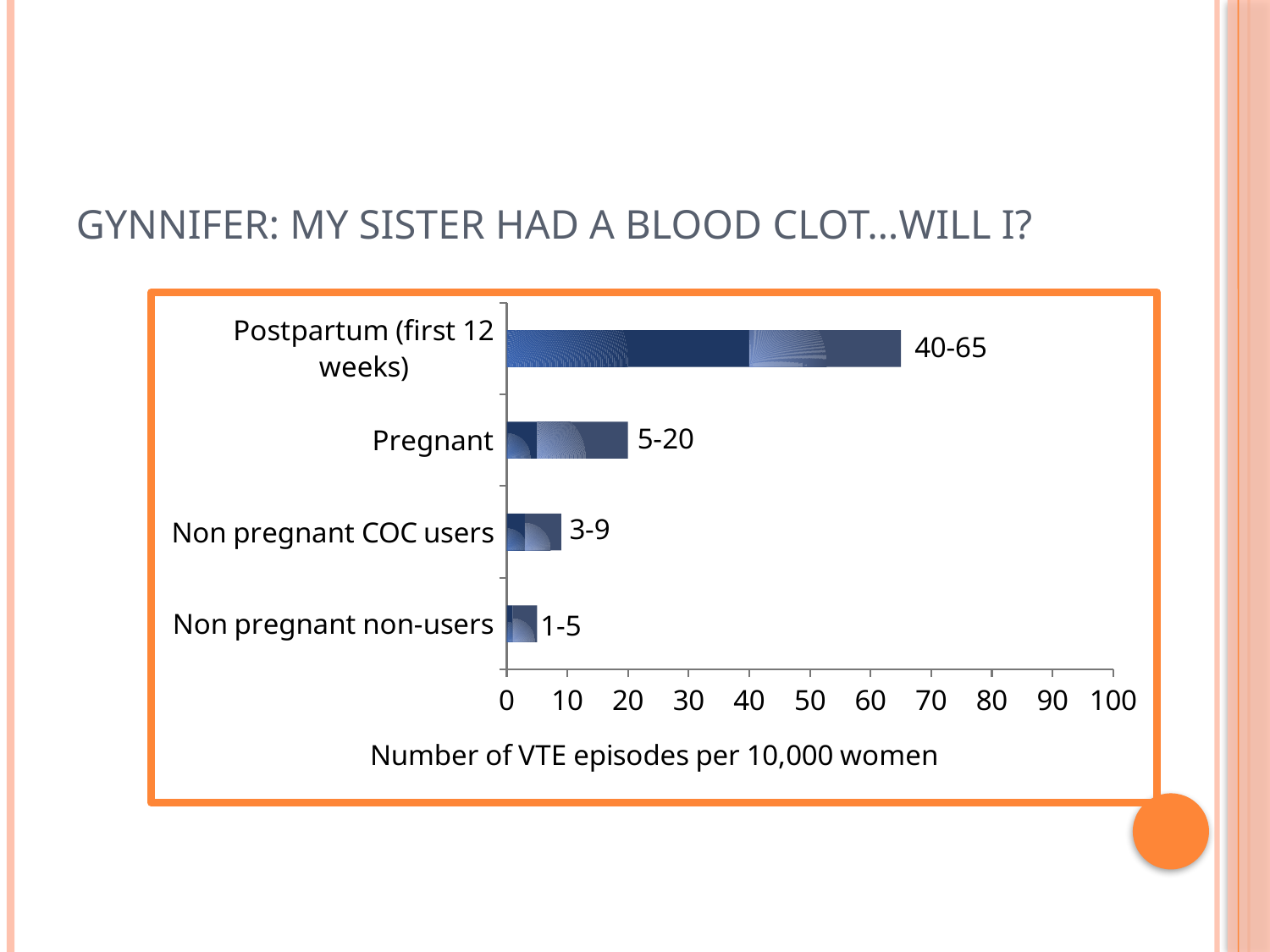

# Gynnifer: My sister had a blood clot…will i?
### Chart
| Category | Number | Column1 |
|---|---|---|
| Non pregnant non-users | 1.0 | 4.0 |
| Non pregnant COC users | 3.0 | 6.0 |
| Pregnant | 5.0 | 15.0 |
| Postpartum (first 12 weeks) | 40.0 | 25.0 |

## Slide 17
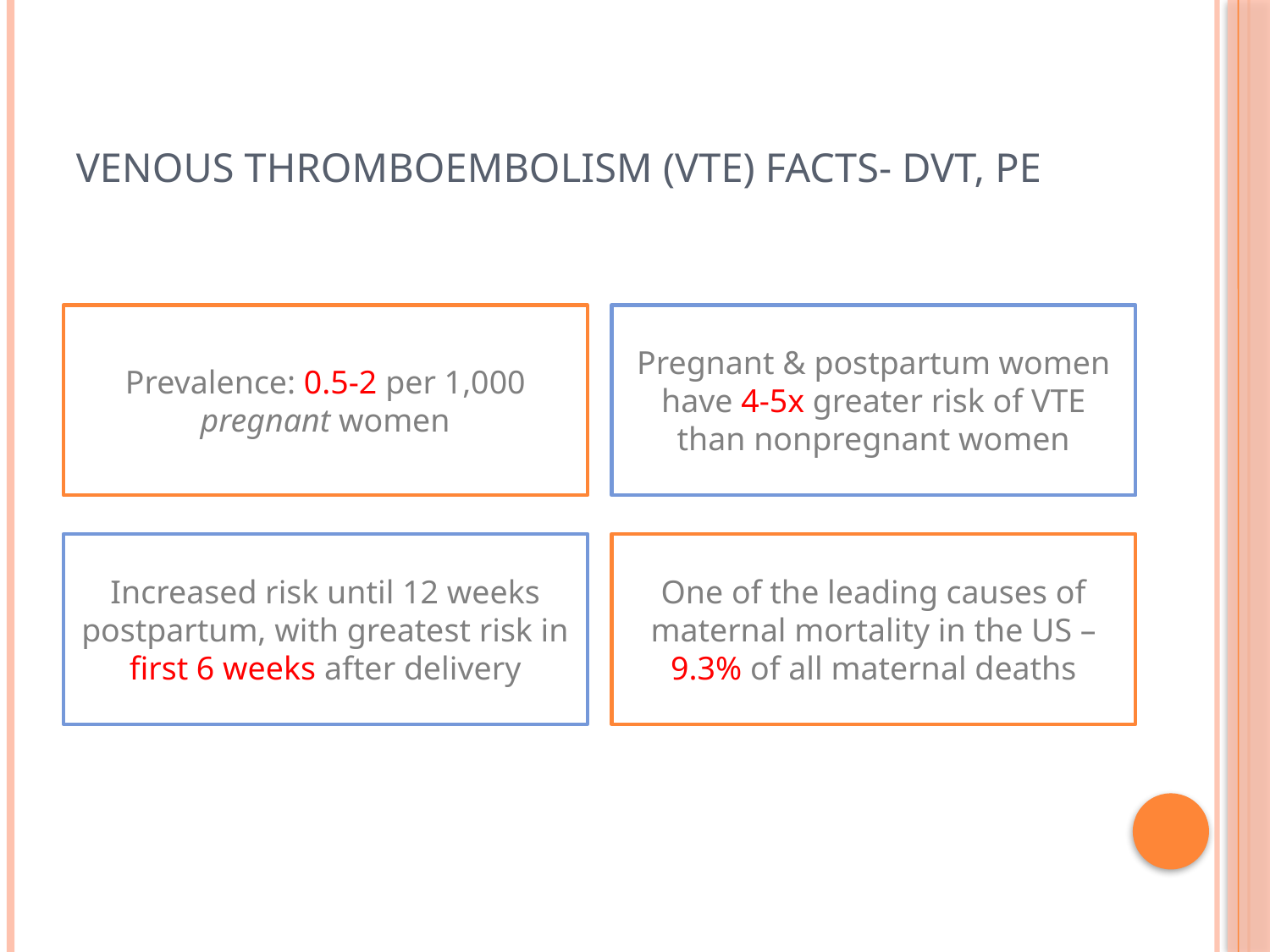

# Venous Thromboembolism (VTE) Facts- DVT, PE
Prevalence: 0.5-2 per 1,000 pregnant women
Pregnant & postpartum women have 4-5x greater risk of VTE than nonpregnant women
Increased risk until 12 weeks postpartum, with greatest risk in first 6 weeks after delivery
One of the leading causes of maternal mortality in the US – 9.3% of all maternal deaths

## Slide 18
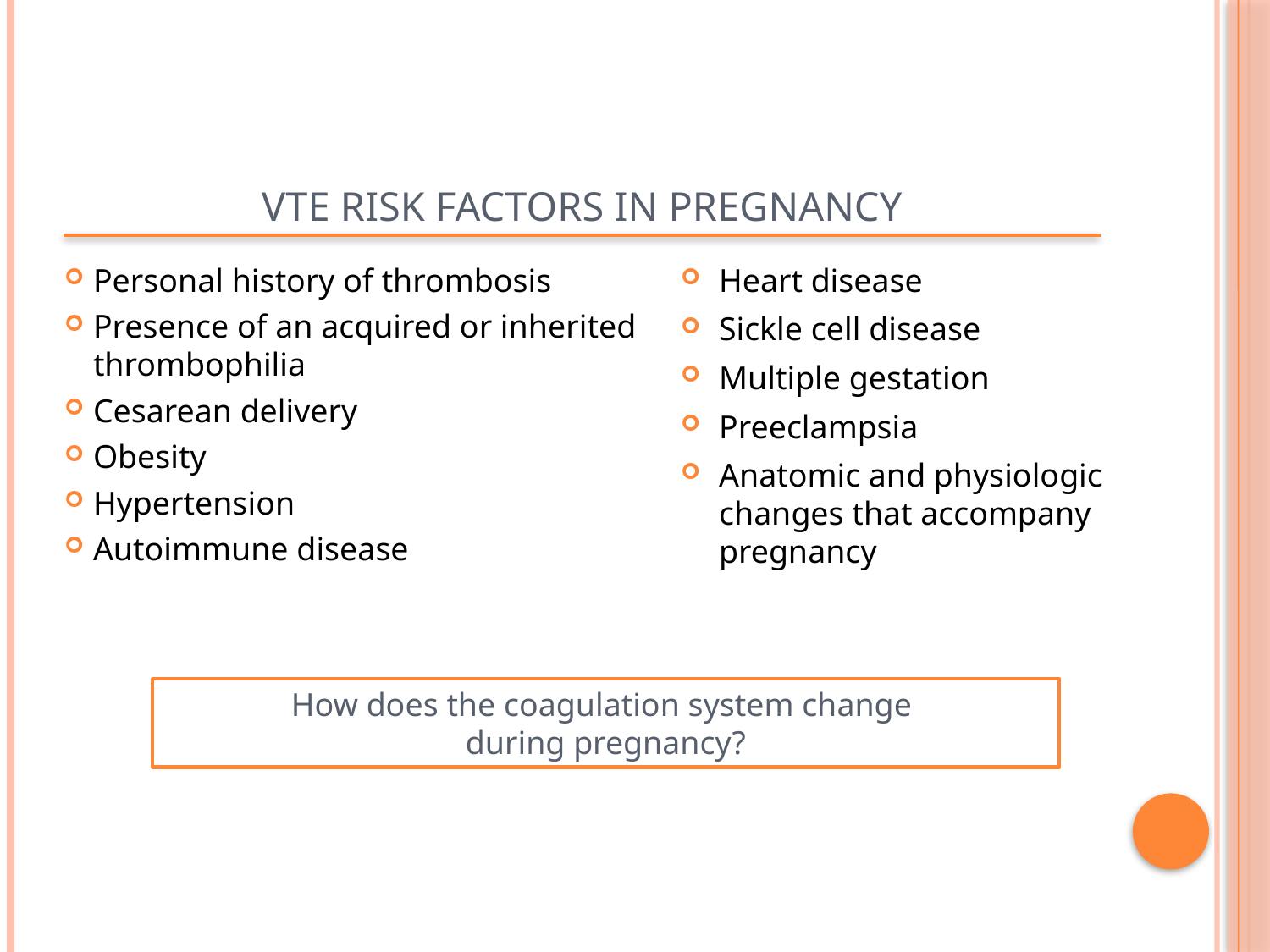

# VTE Risk Factors in pregnancy
Heart disease
Sickle cell disease
Multiple gestation
Preeclampsia
Anatomic and physiologic changes that accompany pregnancy
Personal history of thrombosis
Presence of an acquired or inherited thrombophilia
Cesarean delivery
Obesity
Hypertension
Autoimmune disease
How does the coagulation system change
during pregnancy?

## Slide 19
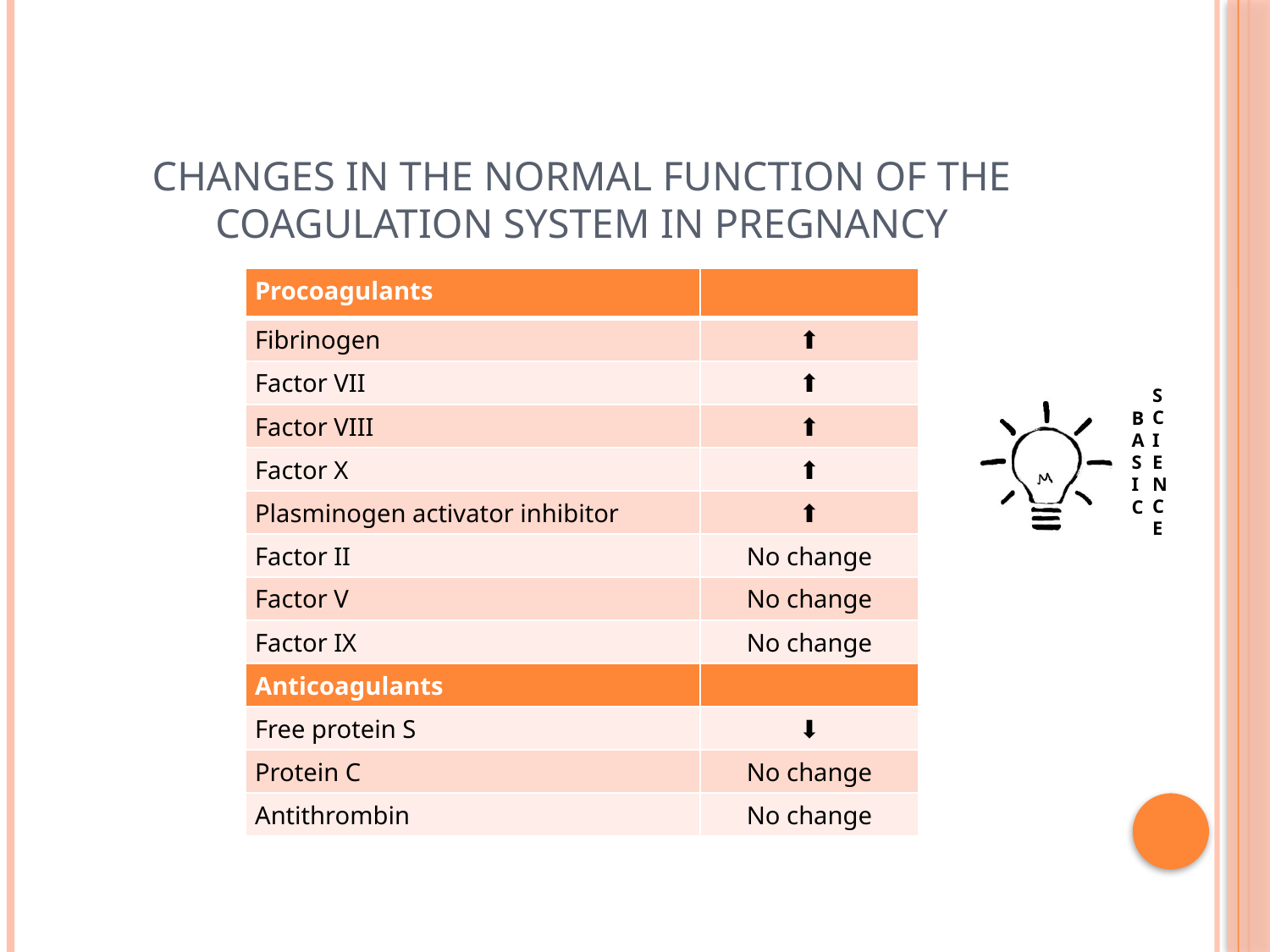

# Changes in the normal function of the coagulation system in pregnancy
| Procoagulants | |
| --- | --- |
| Fibrinogen | ⬆ |
| Factor VII | ⬆ |
| Factor VIII | ⬆ |
| Factor X | ⬆ |
| Plasminogen activator inhibitor | ⬆ |
| Factor II | No change |
| Factor V | No change |
| Factor IX | No change |
| Anticoagulants | |
| Free protein S | ⬇ |
| Protein C | No change |
| Antithrombin | No change |
S
C
I
E
NC
E
B
A
S
I
C

## Slide 20
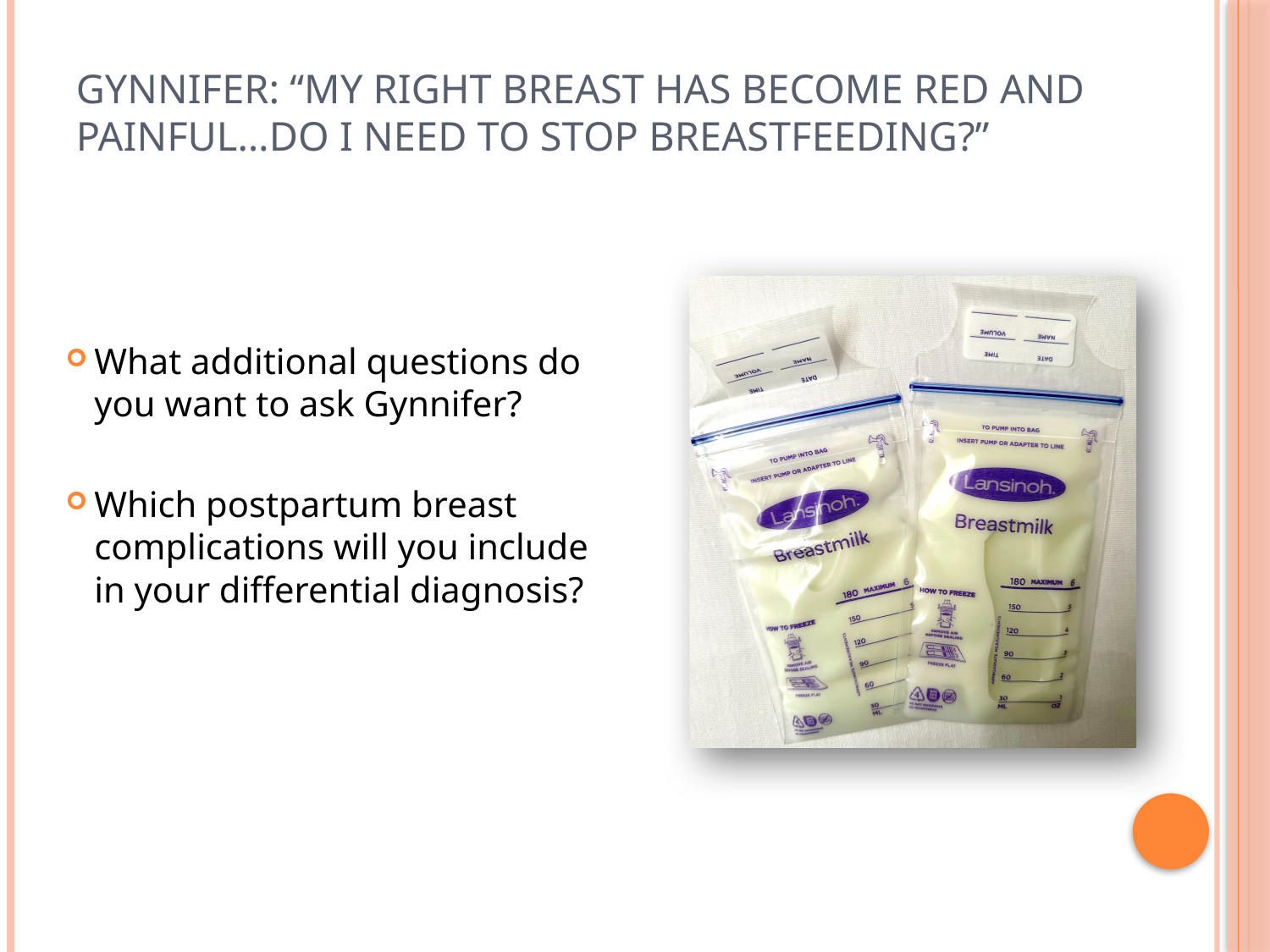

# Gynnifer: “My right breast has become red and painful…do I need to stop breastfeeding?”
What additional questions do you want to ask Gynnifer?
Which postpartum breast complications will you include in your differential diagnosis?

## Slide 21
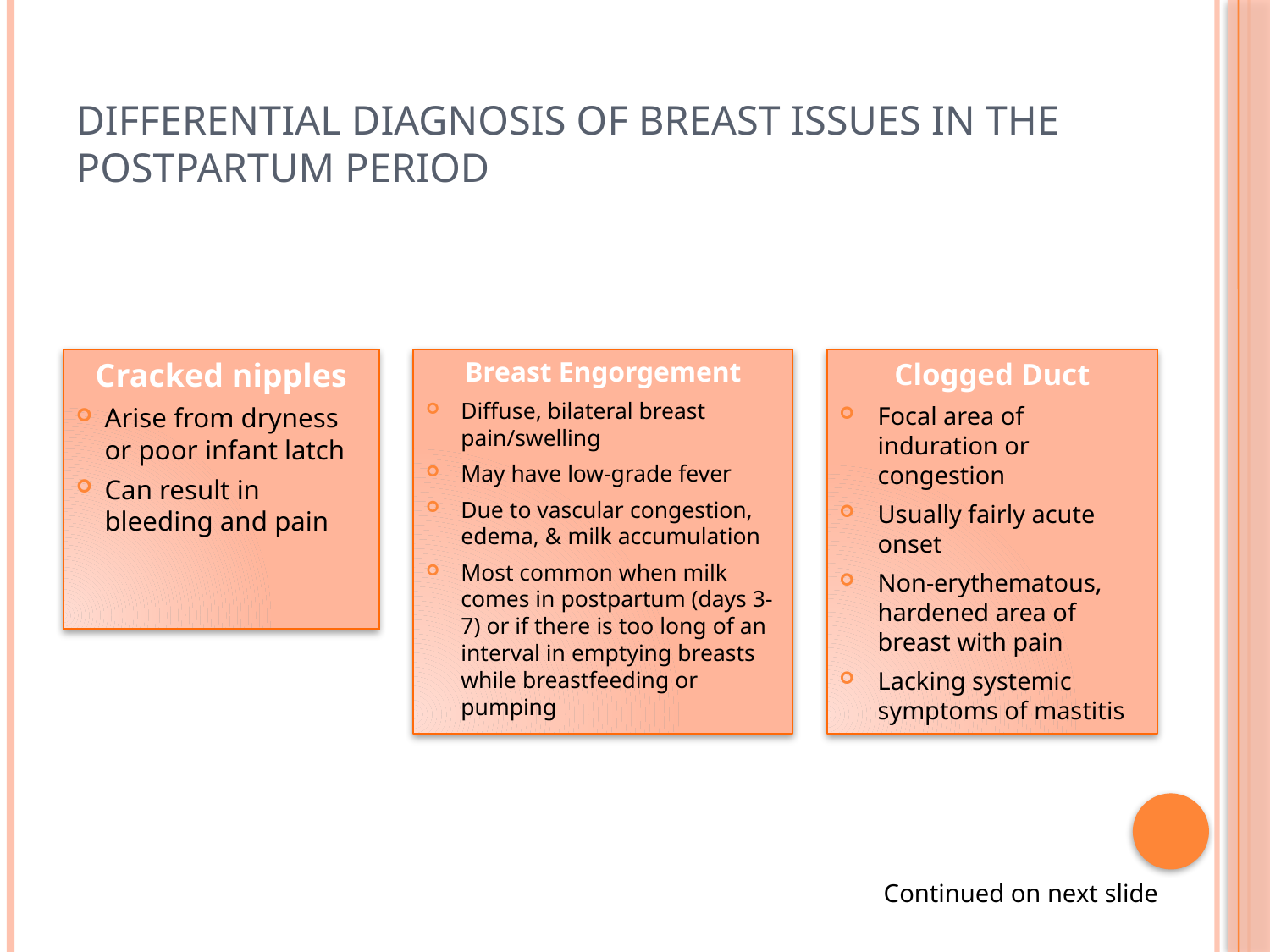

# Differential Diagnosis of breast issues in the postpartum period
Breast Engorgement
Diffuse, bilateral breast pain/swelling
May have low-grade fever
Due to vascular congestion, edema, & milk accumulation
Most common when milk comes in postpartum (days 3-7) or if there is too long of an interval in emptying breasts while breastfeeding or pumping
Cracked nipples
Arise from dryness or poor infant latch
Can result in bleeding and pain
Clogged Duct
Focal area of induration or congestion
Usually fairly acute onset
Non-erythematous, hardened area of breast with pain
Lacking systemic symptoms of mastitis
Continued on next slide

## Slide 22
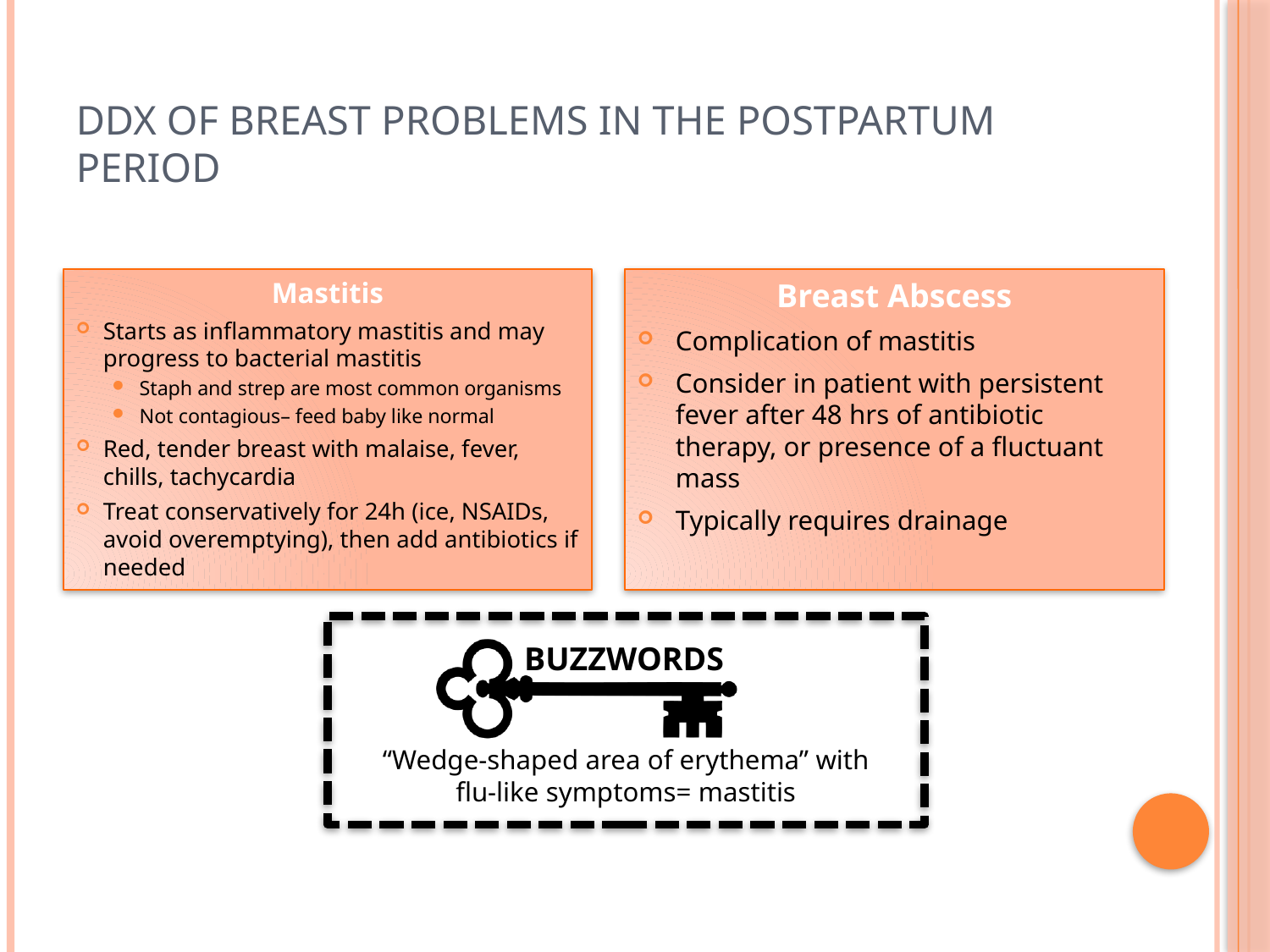

# DDX of breast problems in the postpartum period
Mastitis
Starts as inflammatory mastitis and may progress to bacterial mastitis
Staph and strep are most common organisms
Not contagious– feed baby like normal
Red, tender breast with malaise, fever, chills, tachycardia
Treat conservatively for 24h (ice, NSAIDs, avoid overemptying), then add antibiotics if needed
Breast Abscess
Complication of mastitis
Consider in patient with persistent fever after 48 hrs of antibiotic therapy, or presence of a fluctuant mass
Typically requires drainage
BUZZWORDS
“Wedge-shaped area of erythema” with flu-like symptoms= mastitis

## Slide 23
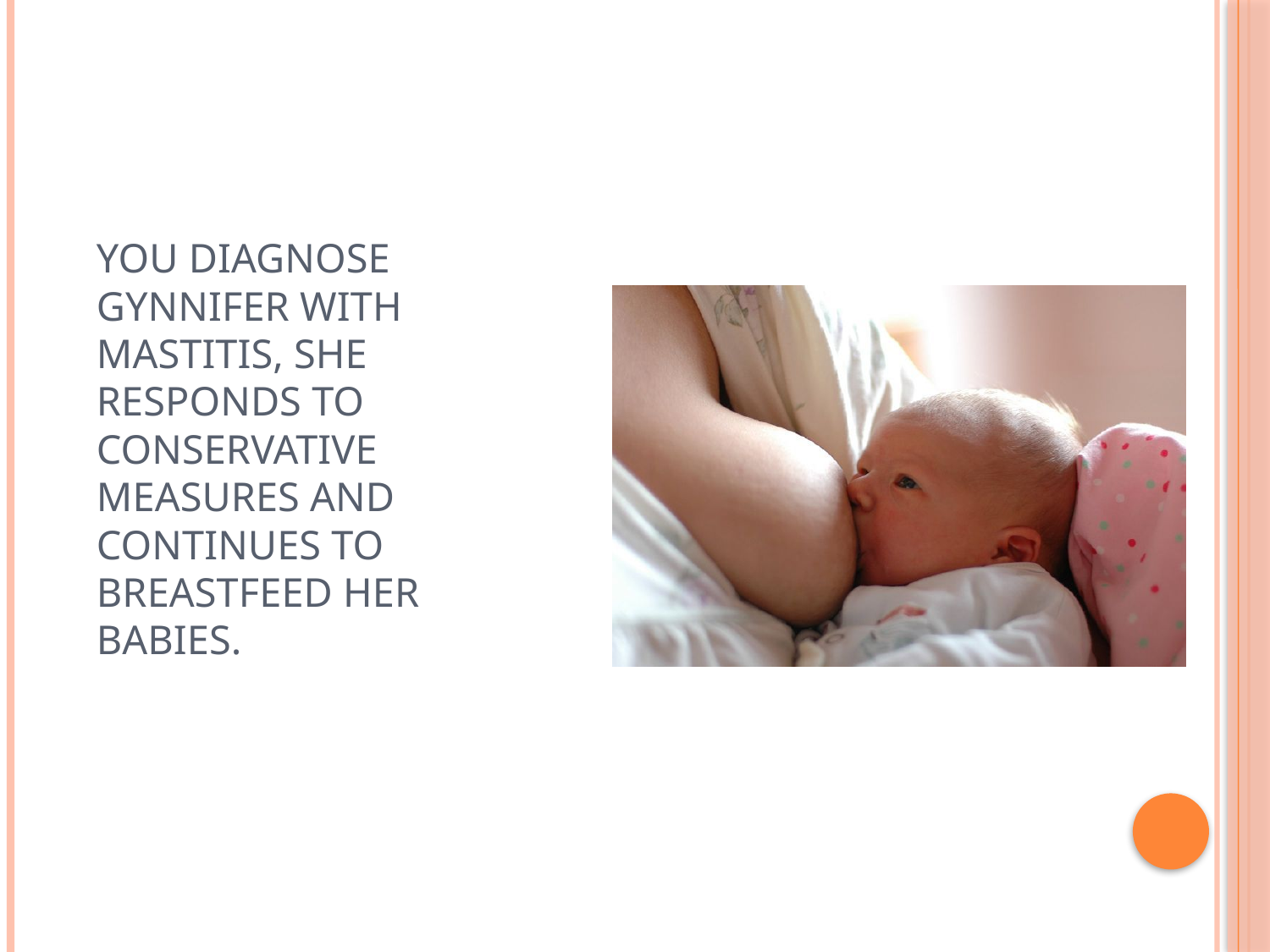

# You diagnose Gynnifer with mastitis, she responds to conservative measures and continues to breastfeed her babies.

## Slide 24
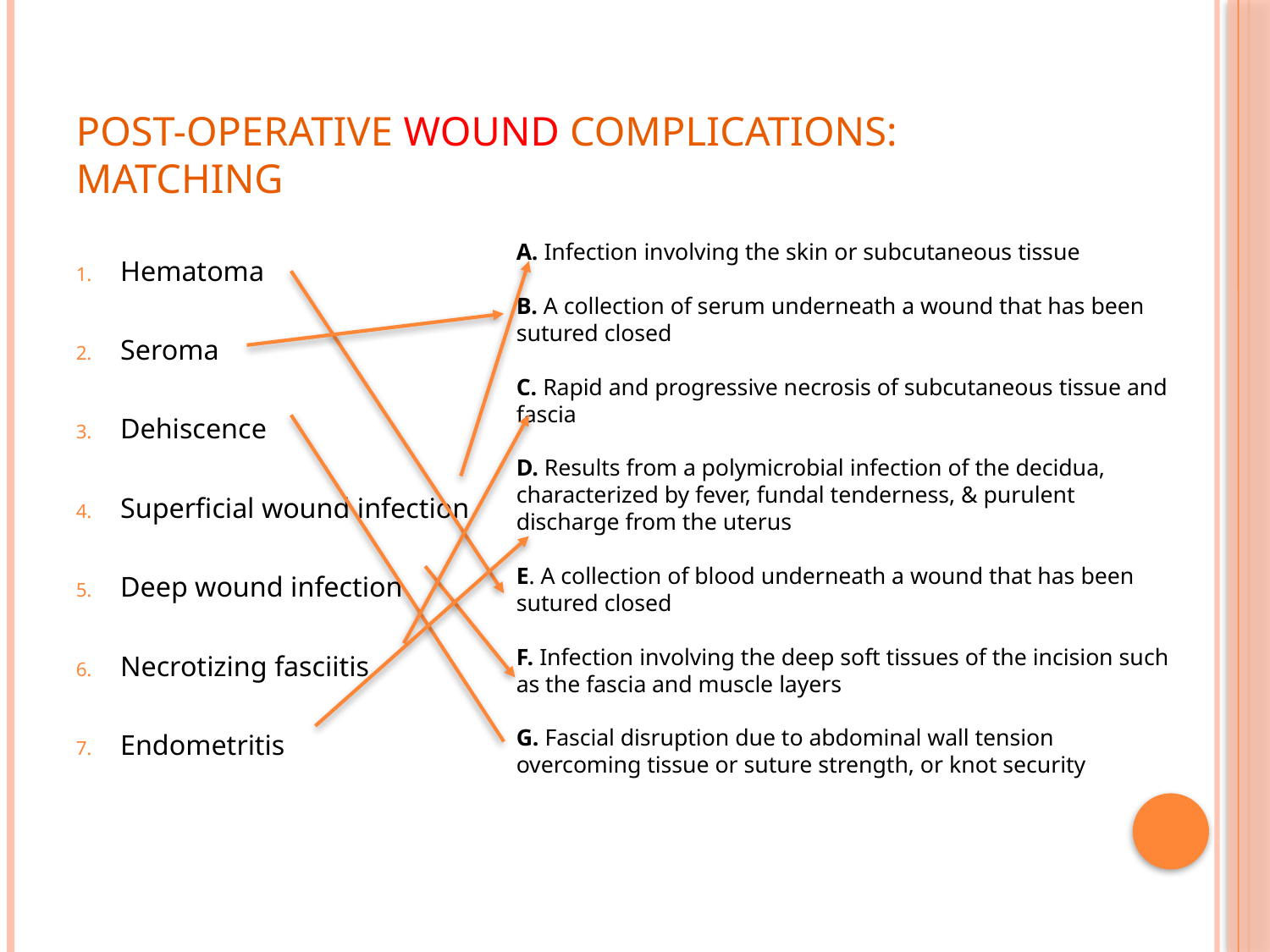

# Post-operative wound complications: MATCHING
A. Infection involving the skin or subcutaneous tissue
B. A collection of serum underneath a wound that has been sutured closed
C. Rapid and progressive necrosis of subcutaneous tissue and fascia
D. Results from a polymicrobial infection of the decidua, characterized by fever, fundal tenderness, & purulent discharge from the uterus
E. A collection of blood underneath a wound that has been sutured closed
F. Infection involving the deep soft tissues of the incision such as the fascia and muscle layers
G. Fascial disruption due to abdominal wall tension overcoming tissue or suture strength, or knot security
Hematoma
Seroma
Dehiscence
Superficial wound infection
Deep wound infection
Necrotizing fasciitis
Endometritis

## Slide 25
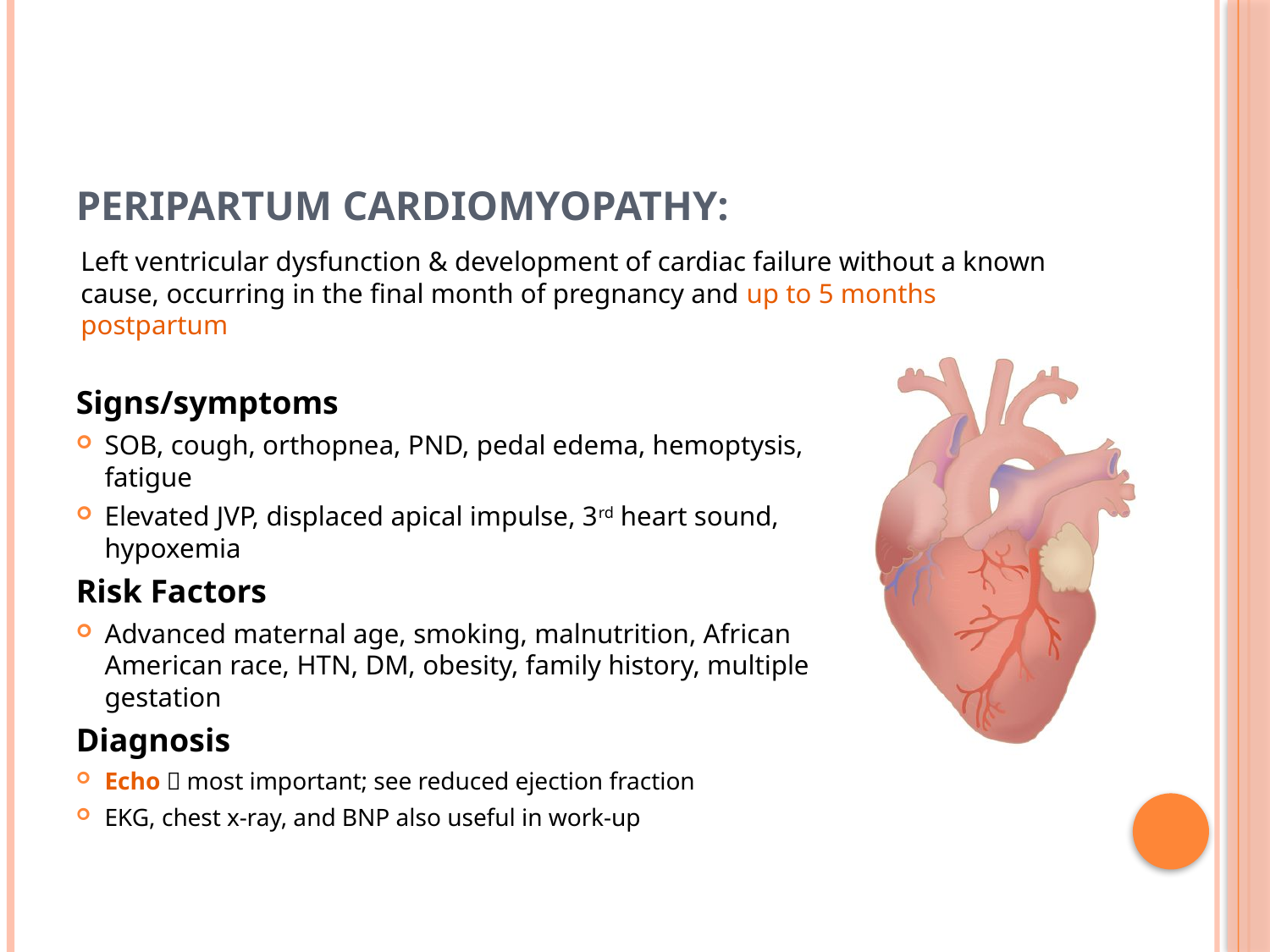

# Peripartum cardiomyopathy:
Left ventricular dysfunction & development of cardiac failure without a known cause, occurring in the final month of pregnancy and up to 5 months postpartum
Signs/symptoms
SOB, cough, orthopnea, PND, pedal edema, hemoptysis, fatigue
Elevated JVP, displaced apical impulse, 3rd heart sound, hypoxemia
Risk Factors
Advanced maternal age, smoking, malnutrition, African American race, HTN, DM, obesity, family history, multiple gestation
Diagnosis
Echo  most important; see reduced ejection fraction
EKG, chest x-ray, and BNP also useful in work-up

## Slide 26
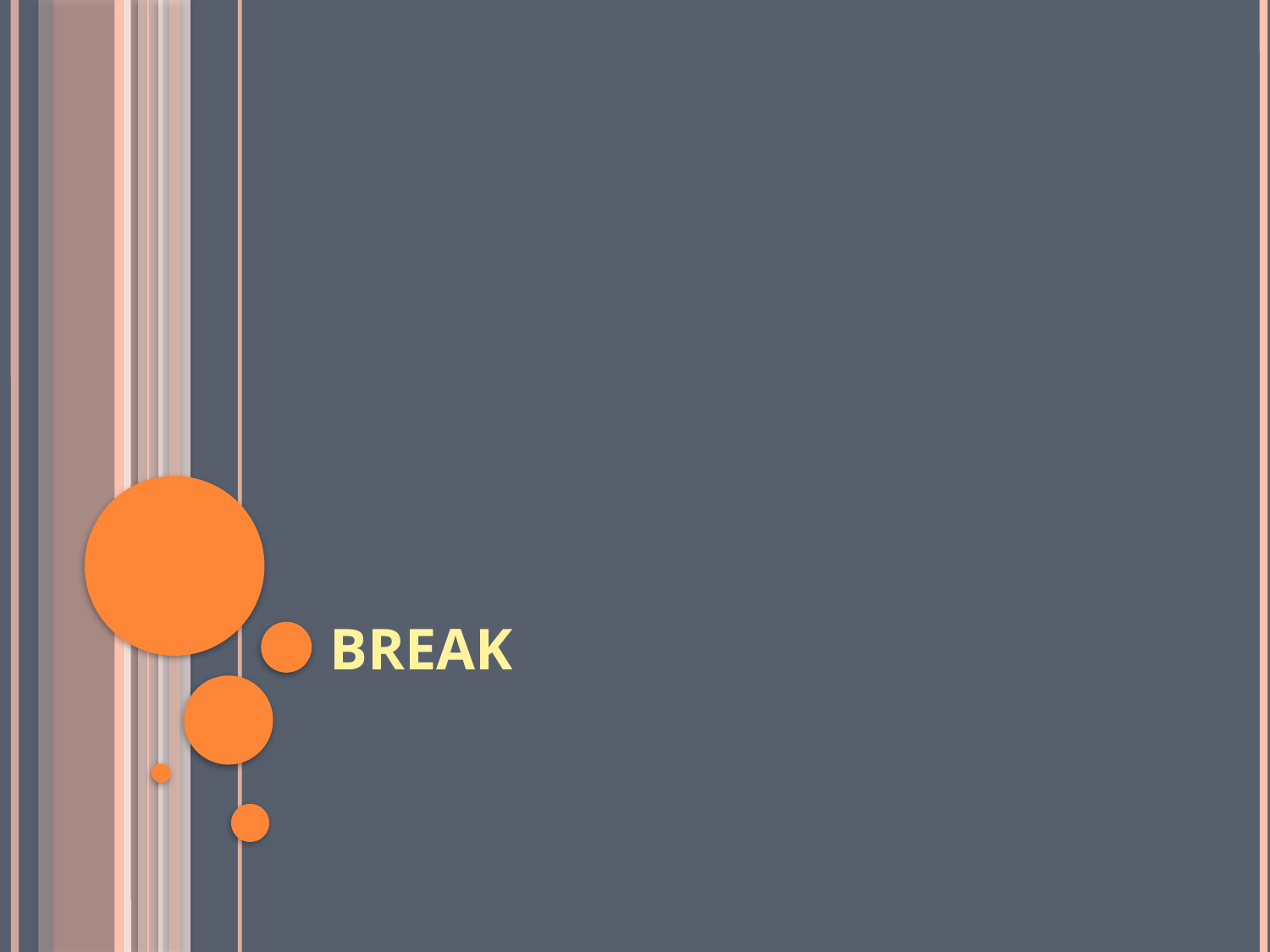

# Break

## Slide 27
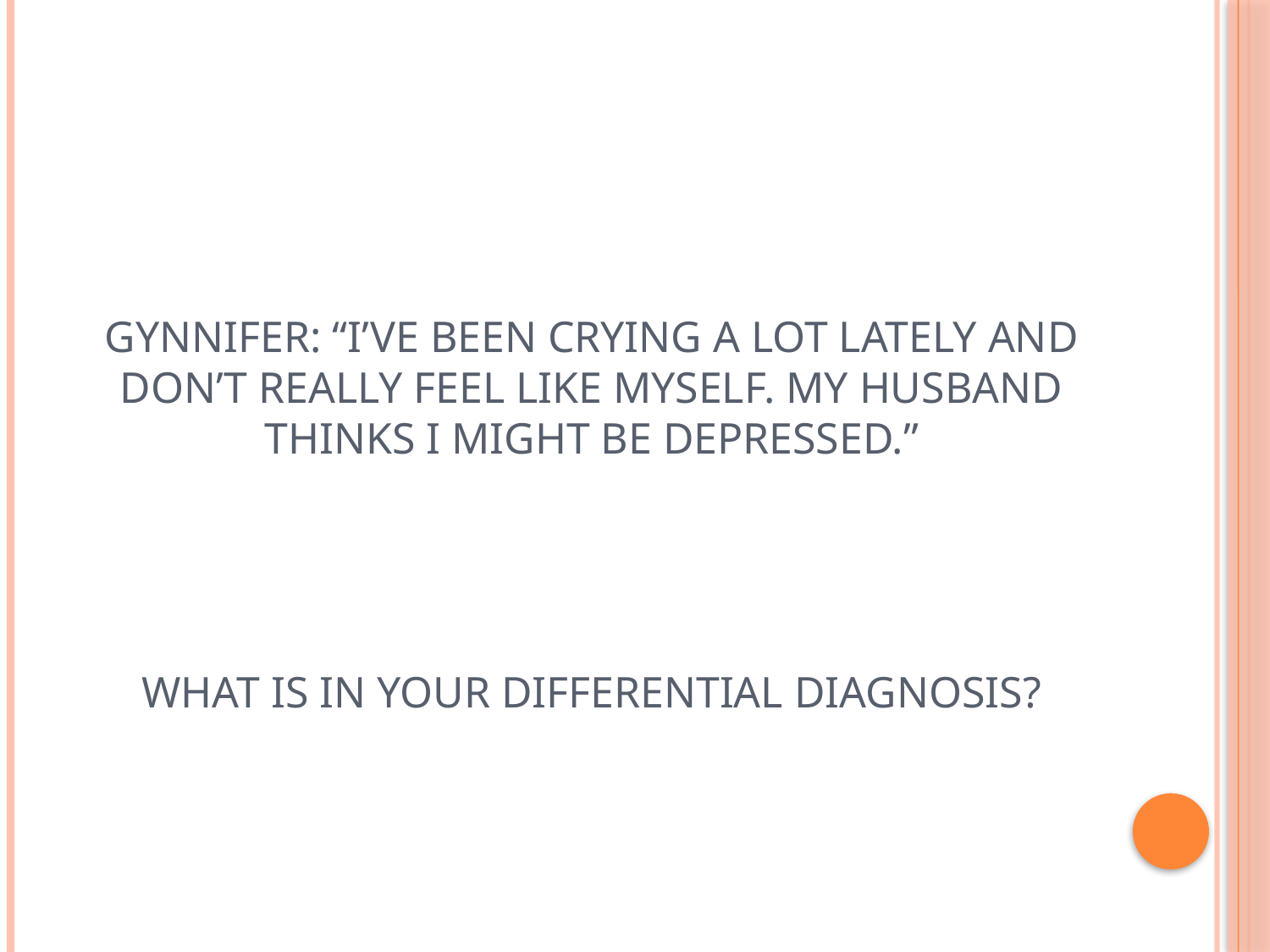

# Gynnifer: “I’ve been crying a lot lately and don’t really feel like myself. My husband thinks I might be depressed.” What is in your differential diagnosis?

## Slide 28
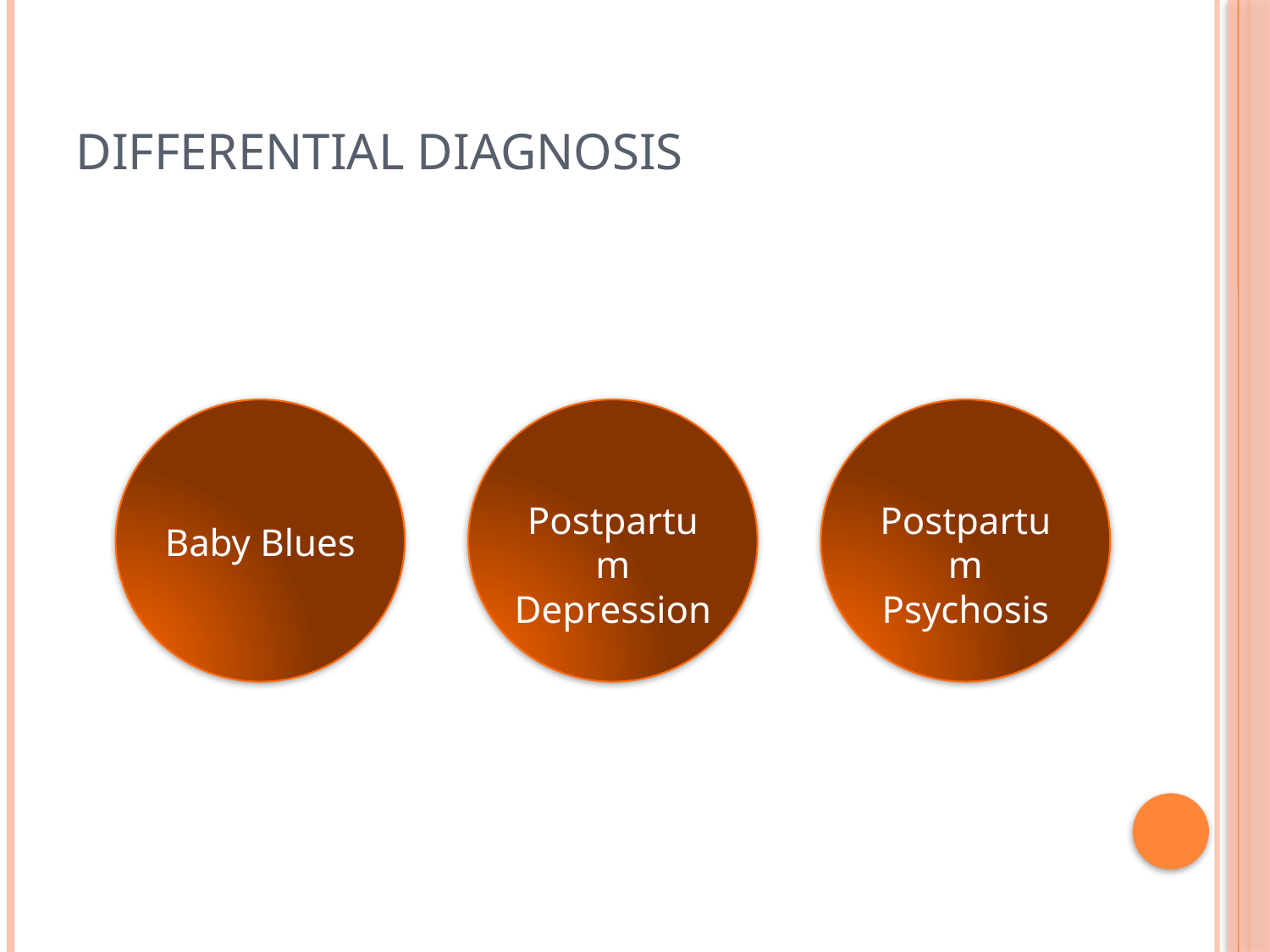

# differential diagnosis
Postpartum Depression
Postpartum Psychosis
Baby Blues

## Slide 29
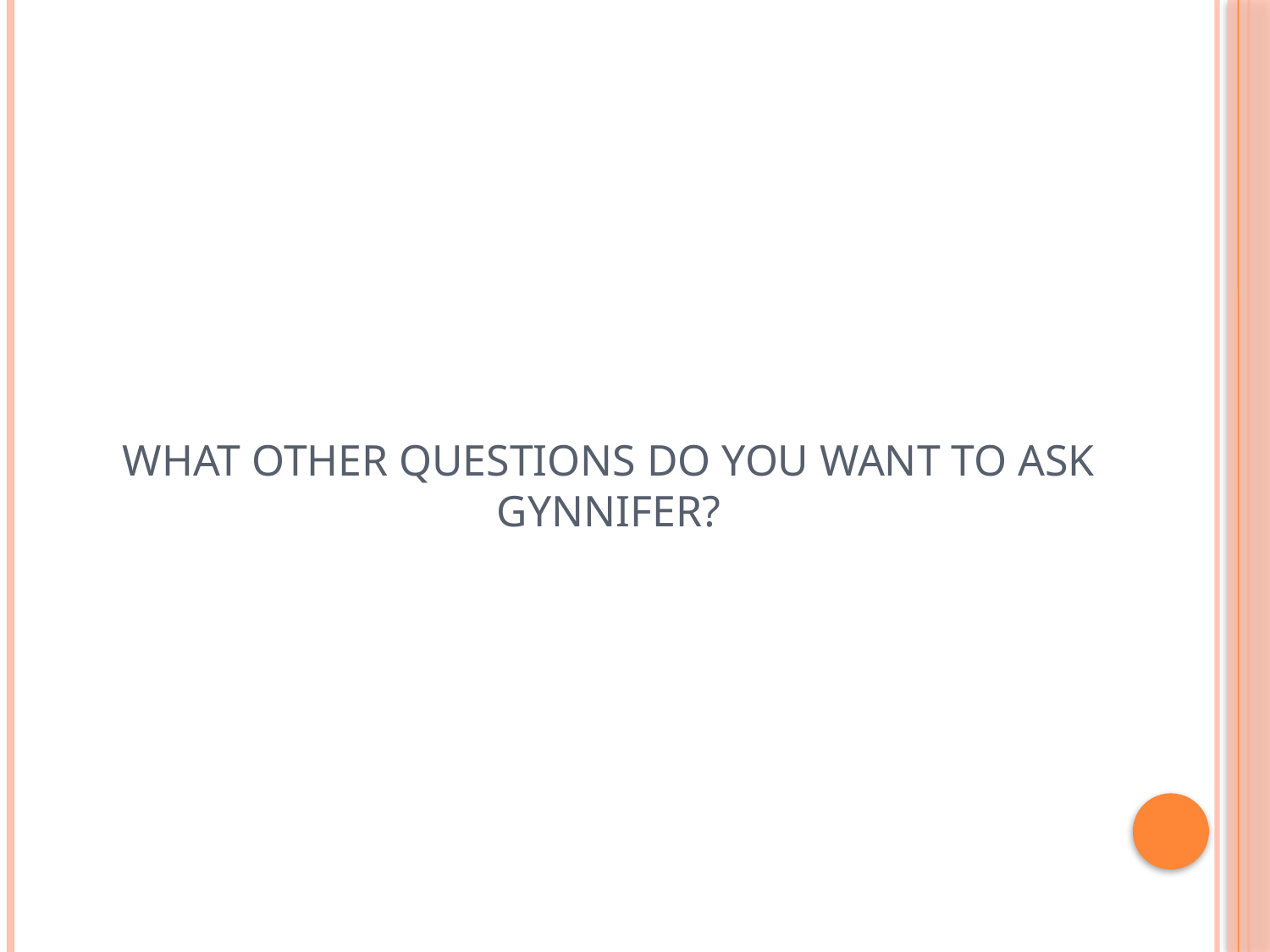

# What other questions do you want to ask gynnifer?

## Slide 30
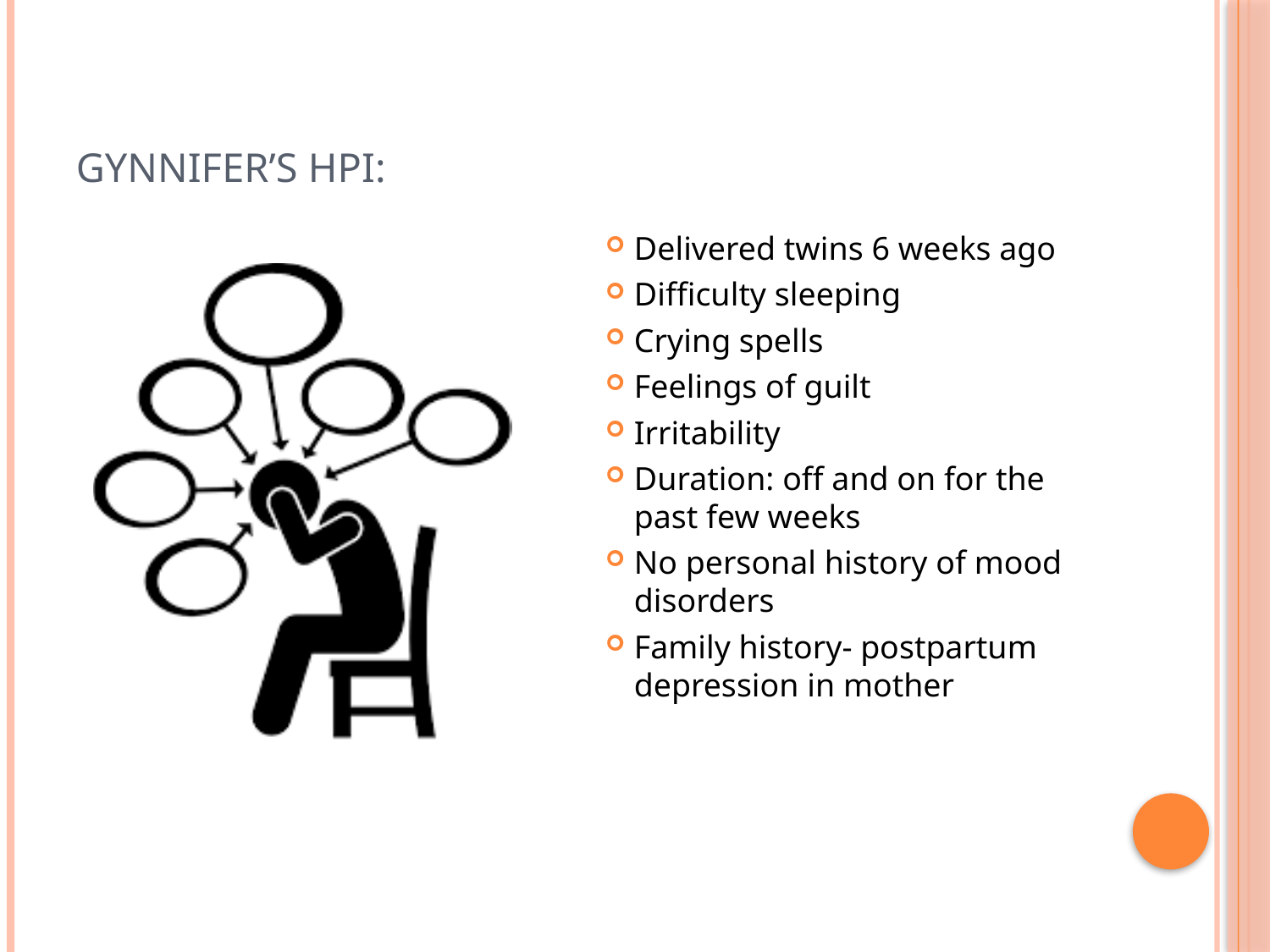

# Gynnifer’s HPI:
Delivered twins 6 weeks ago
Difficulty sleeping
Crying spells
Feelings of guilt
Irritability
Duration: off and on for the past few weeks
No personal history of mood disorders
Family history- postpartum depression in mother

## Slide 31
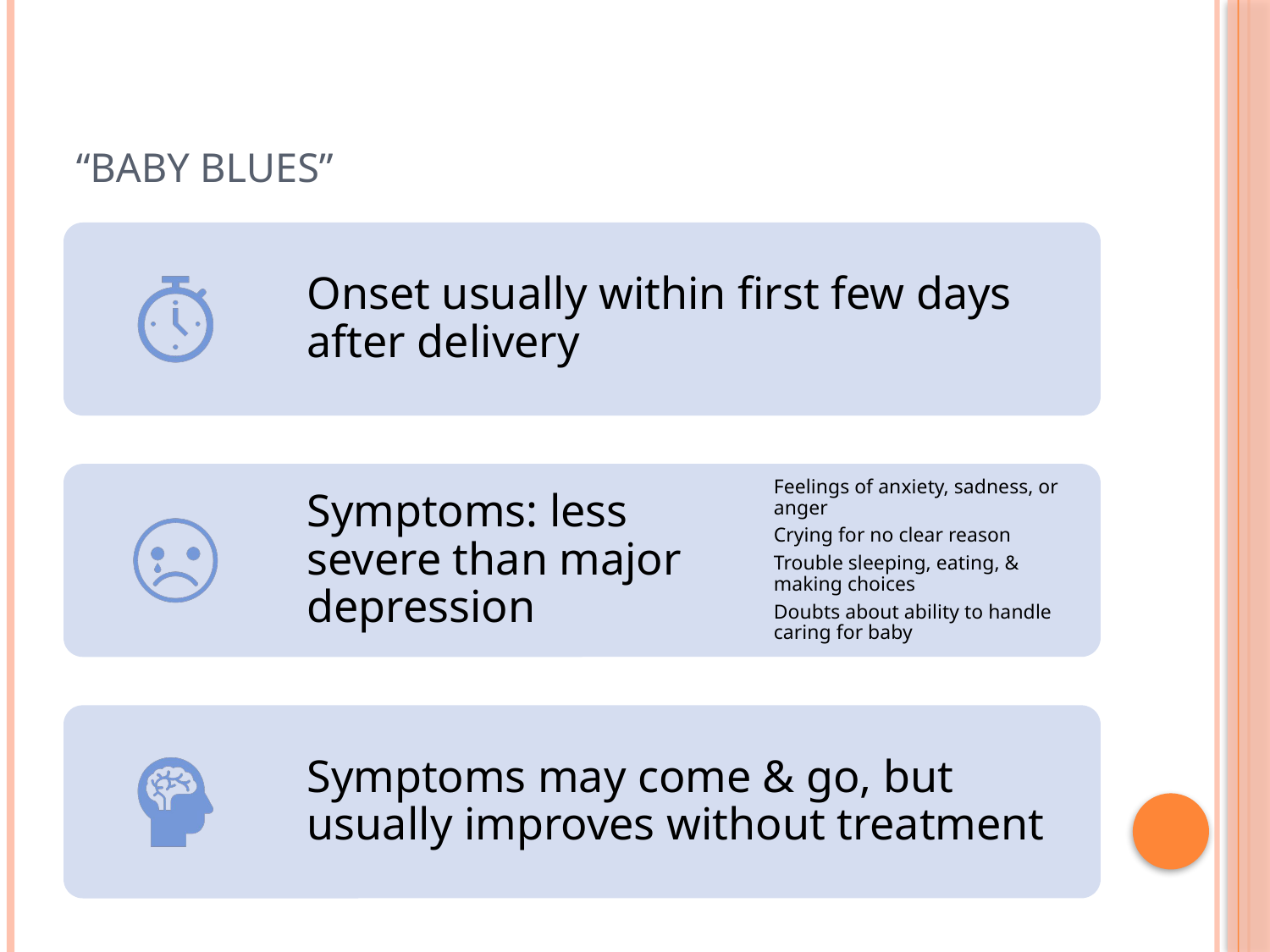

# “Baby Blues”

## Slide 32
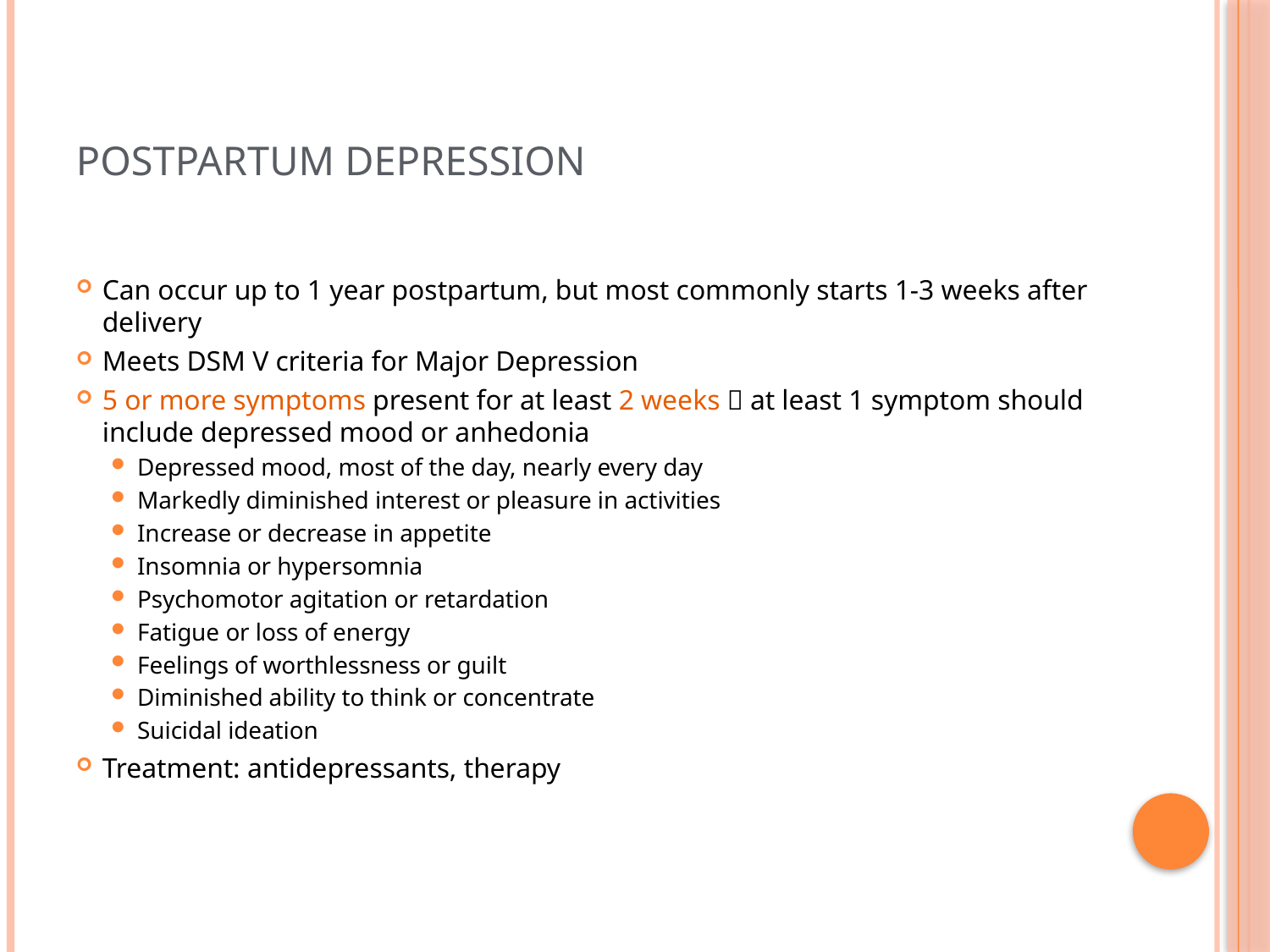

# Postpartum Depression
Can occur up to 1 year postpartum, but most commonly starts 1-3 weeks after delivery
Meets DSM V criteria for Major Depression
5 or more symptoms present for at least 2 weeks  at least 1 symptom should include depressed mood or anhedonia
Depressed mood, most of the day, nearly every day
Markedly diminished interest or pleasure in activities
Increase or decrease in appetite
Insomnia or hypersomnia
Psychomotor agitation or retardation
Fatigue or loss of energy
Feelings of worthlessness or guilt
Diminished ability to think or concentrate
Suicidal ideation
Treatment: antidepressants, therapy

## Slide 33
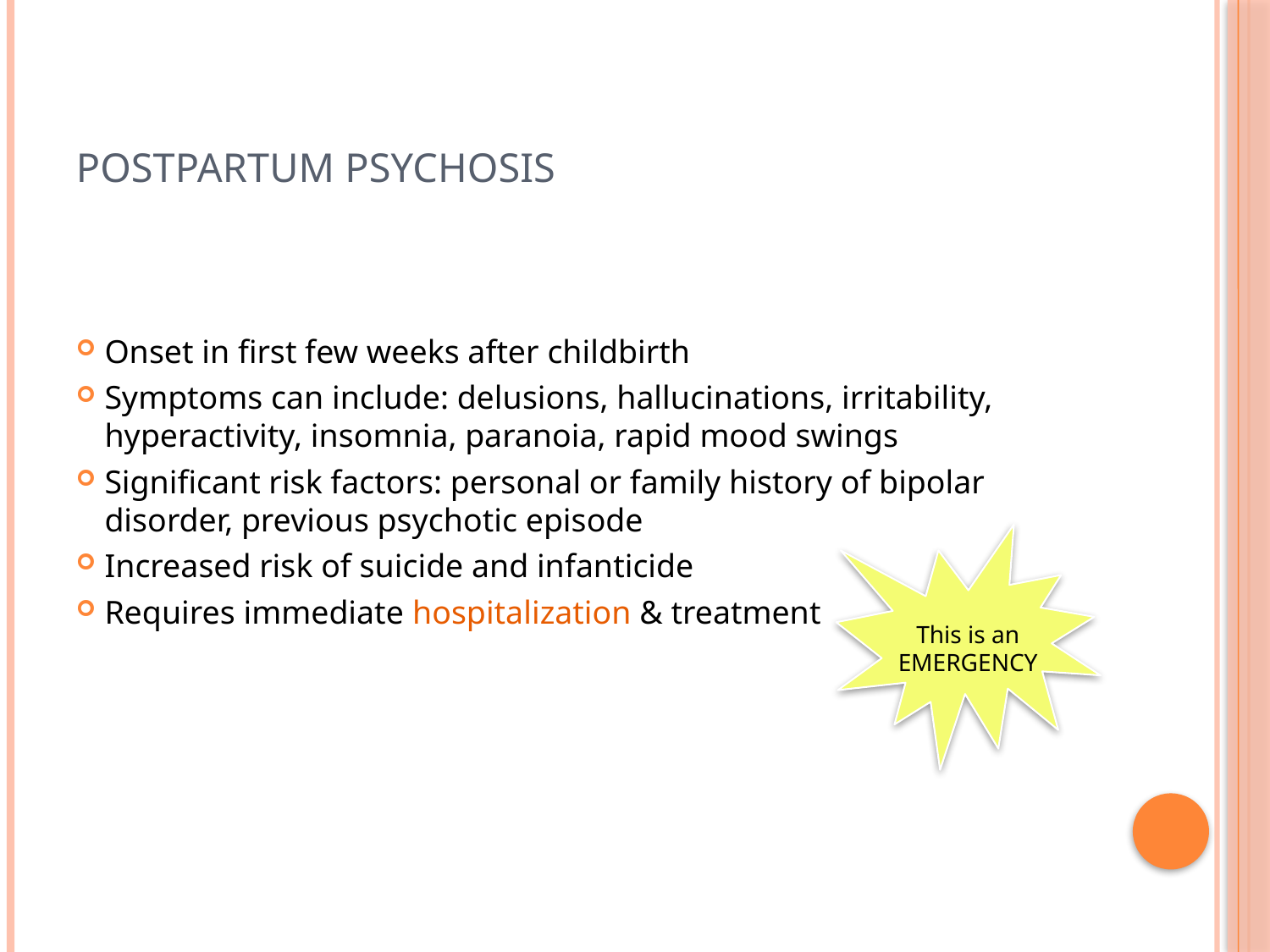

# Postpartum psychosis
Onset in first few weeks after childbirth
Symptoms can include: delusions, hallucinations, irritability, hyperactivity, insomnia, paranoia, rapid mood swings
Significant risk factors: personal or family history of bipolar disorder, previous psychotic episode
Increased risk of suicide and infanticide
Requires immediate hospitalization & treatment
This is an EMERGENCY

## Slide 34
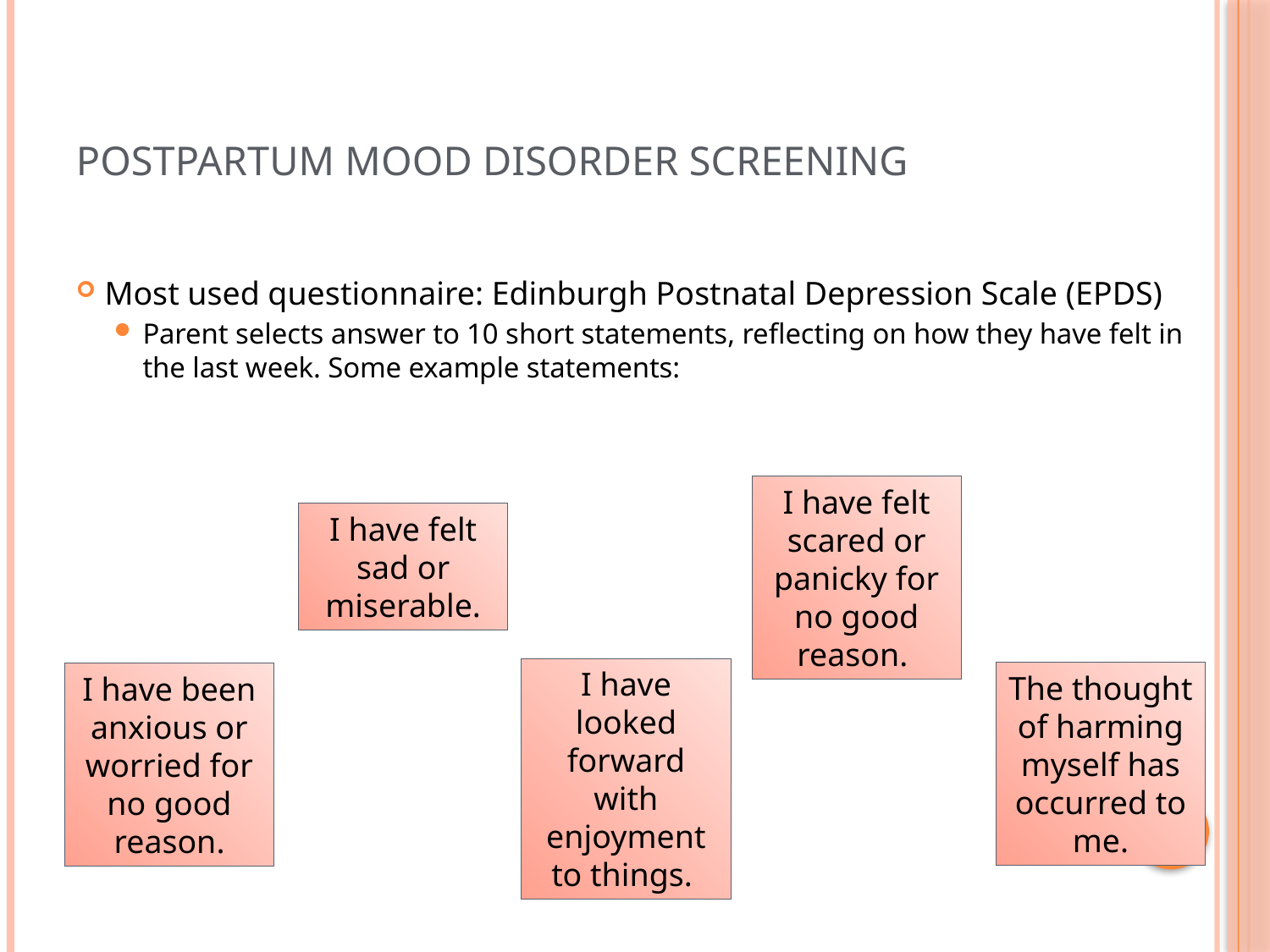

# Postpartum Mood Disorder Screening
Most used questionnaire: Edinburgh Postnatal Depression Scale (EPDS)
Parent selects answer to 10 short statements, reflecting on how they have felt in the last week. Some example statements:
I have felt scared or panicky for no good reason.
I have felt sad or miserable.
I have looked forward with enjoyment to things.
The thought of harming myself has occurred to me.
I have been anxious or worried for no good reason.

## Slide 35
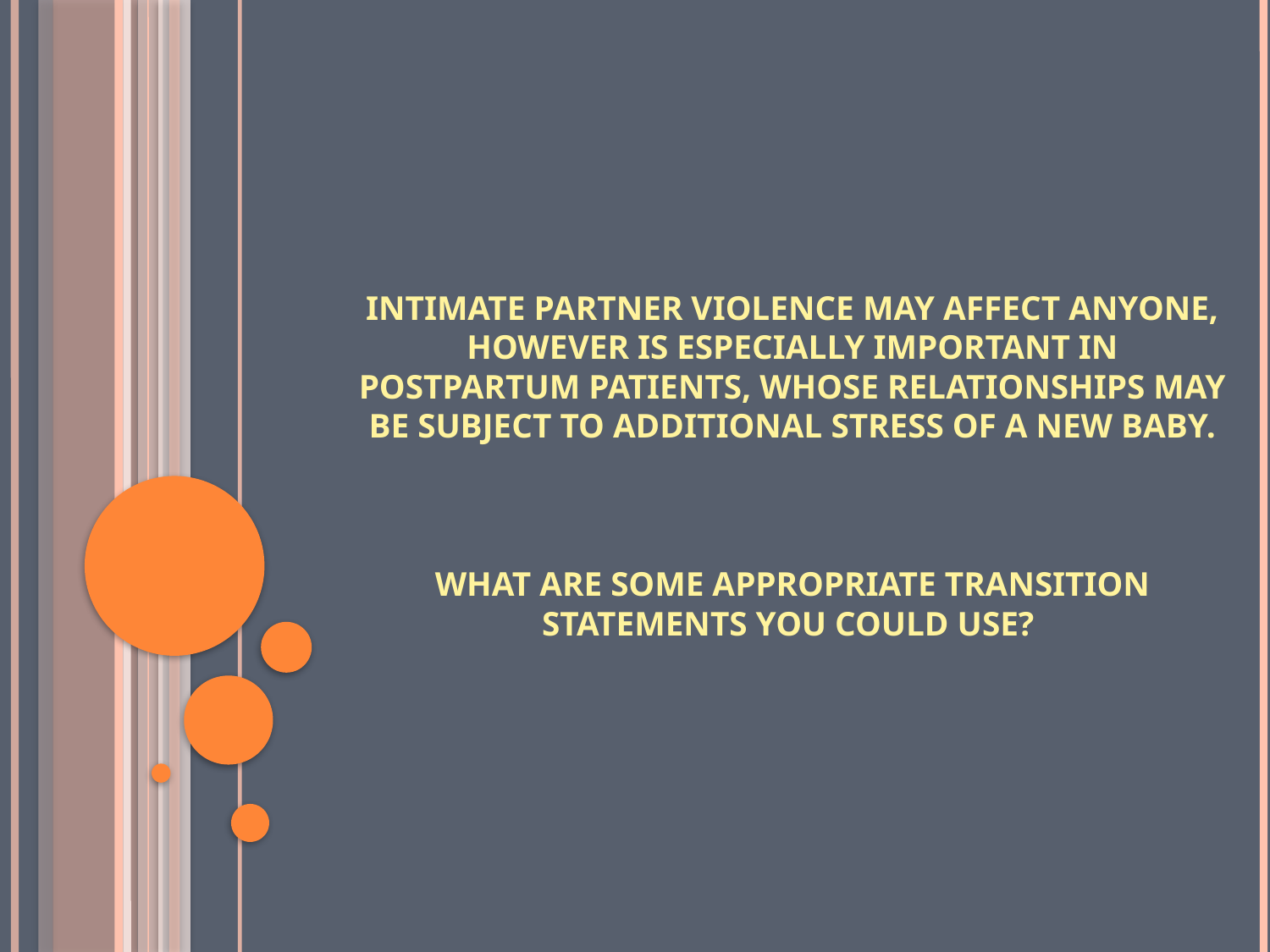

# intimate partner violence may affect anyone, however is especially important in postpartum patients, whose relationships may be subject to additional stress of a new baby. What are some appropriate transition statements you could use?

## Slide 36
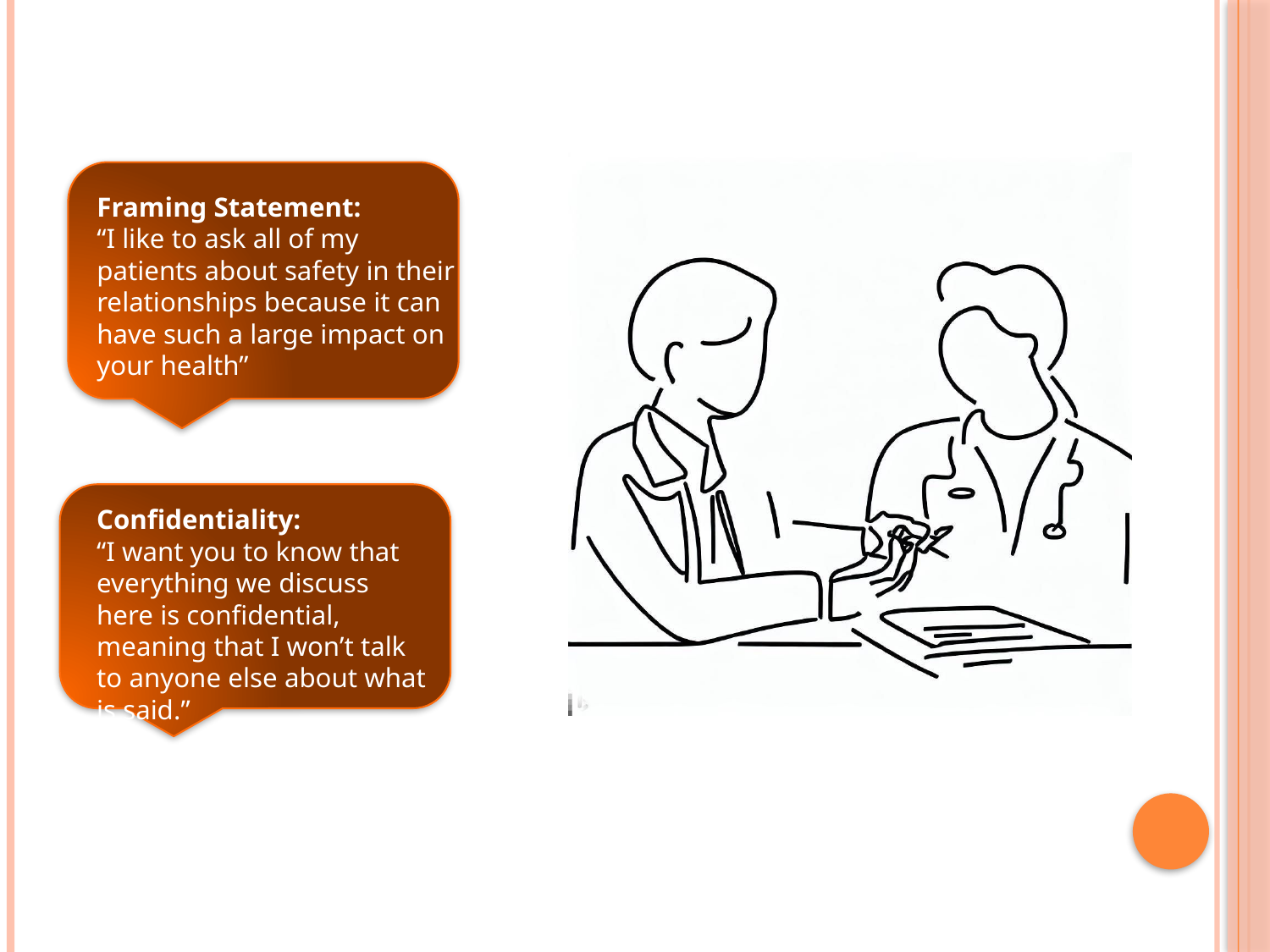

Framing Statement:
“I like to ask all of my patients about safety in their relationships because it can have such a large impact on your health”
Confidentiality:
“I want you to know that everything we discuss here is confidential, meaning that I won’t talk to anyone else about what is said.”

## Slide 37
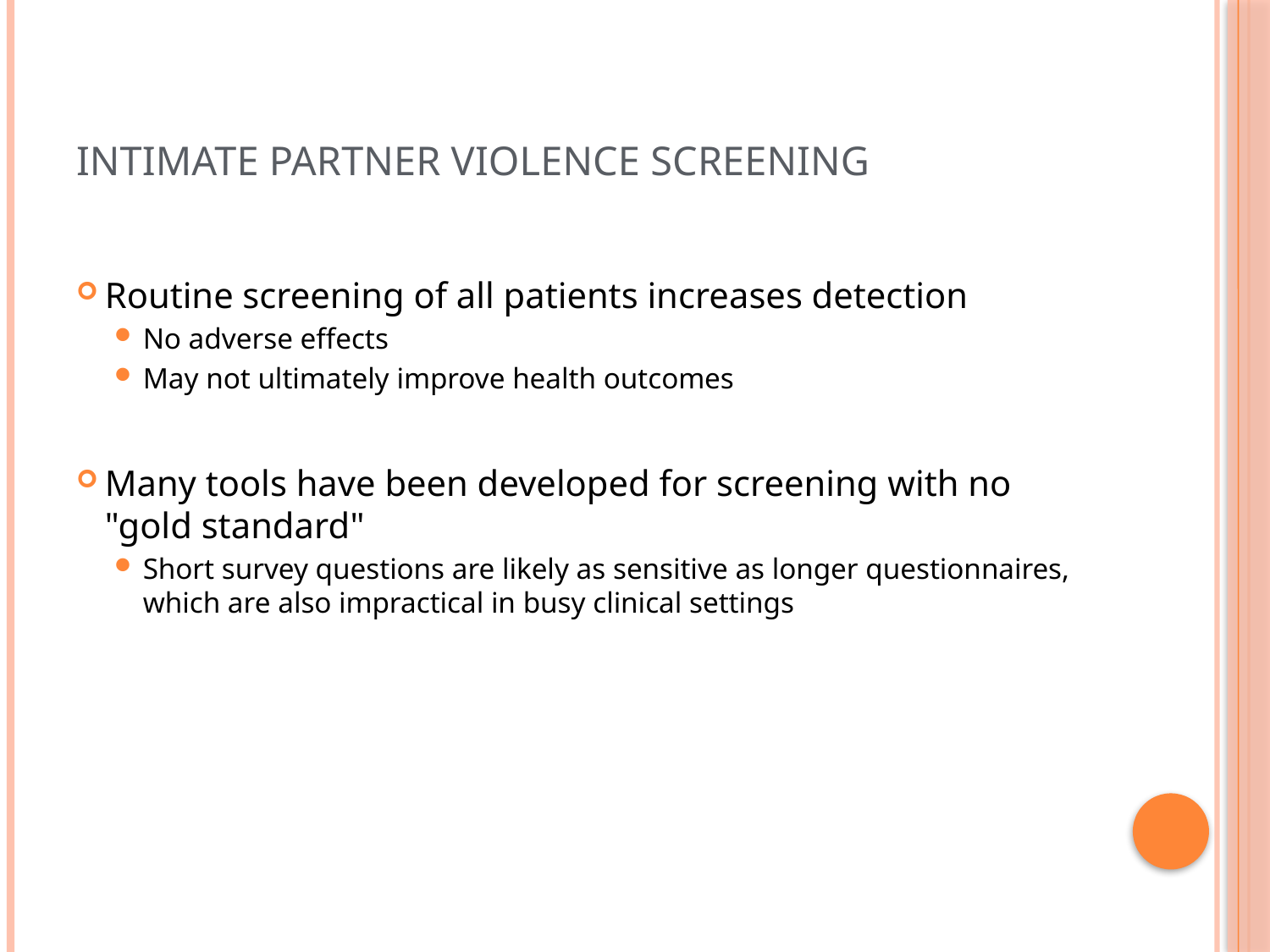

# Intimate Partner Violence Screening
Routine screening of all patients increases detection
No adverse effects
May not ultimately improve health outcomes
Many tools have been developed for screening with no "gold standard"
Short survey questions are likely as sensitive as longer questionnaires, which are also impractical in busy clinical settings

## Slide 38
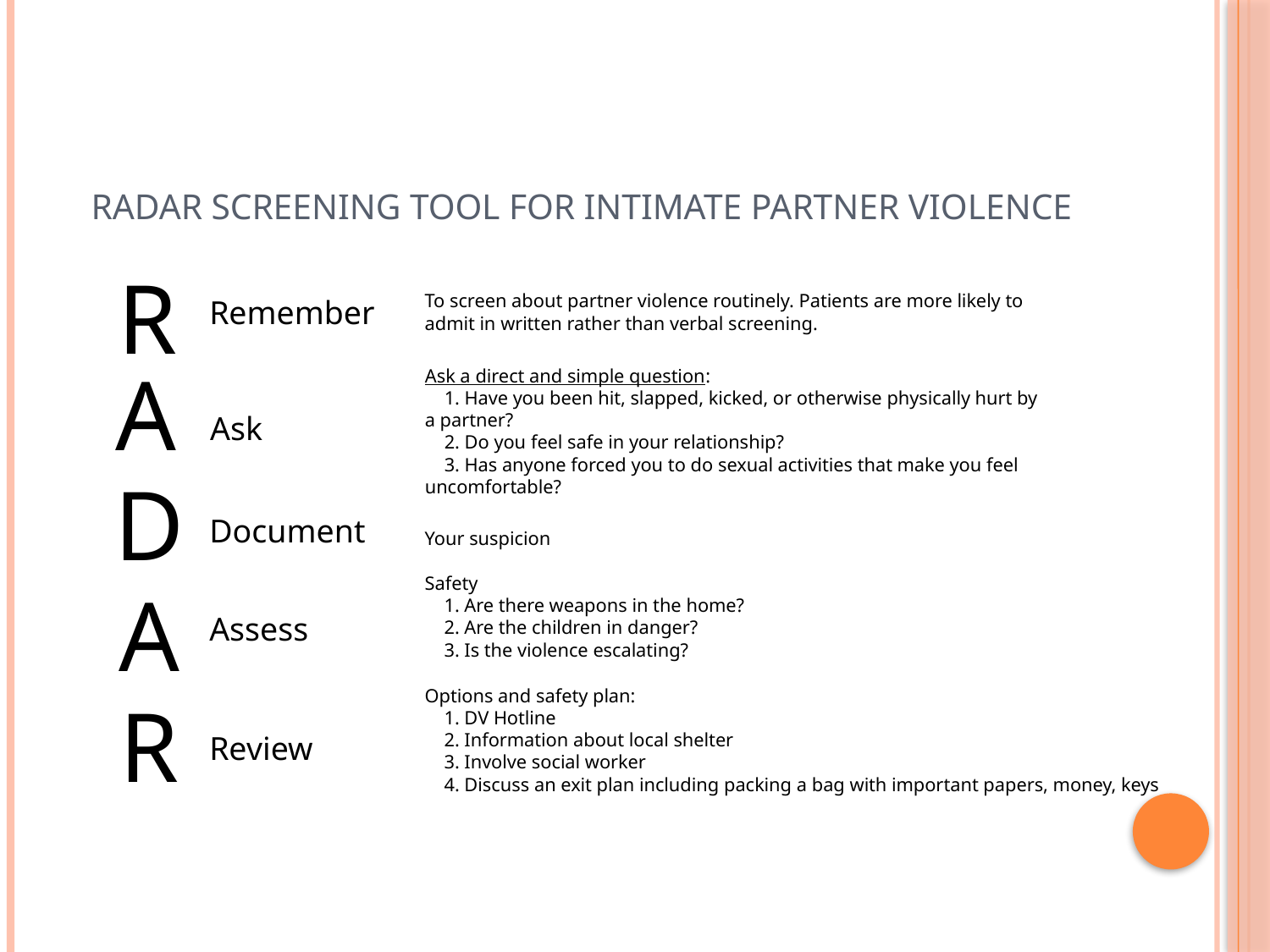

# RADAR Screening Tool for Intimate Partner Violence
R
To screen about partner violence routinely. Patients are more likely to admit in written rather than verbal screening.
Remember
A
Ask a direct and simple question:
 1. Have you been hit, slapped, kicked, or otherwise physically hurt by a partner?
 2. Do you feel safe in your relationship?
 3. Has anyone forced you to do sexual activities that make you feel uncomfortable?
Ask
D
Document
Your suspicion
Safety
 1. Are there weapons in the home?
 2. Are the children in danger?
 3. Is the violence escalating?
A
Assess
Options and safety plan:
 1. DV Hotline
 2. Information about local shelter
 3. Involve social worker
 4. Discuss an exit plan including packing a bag with important papers, money, keys
R
Review

## Slide 39
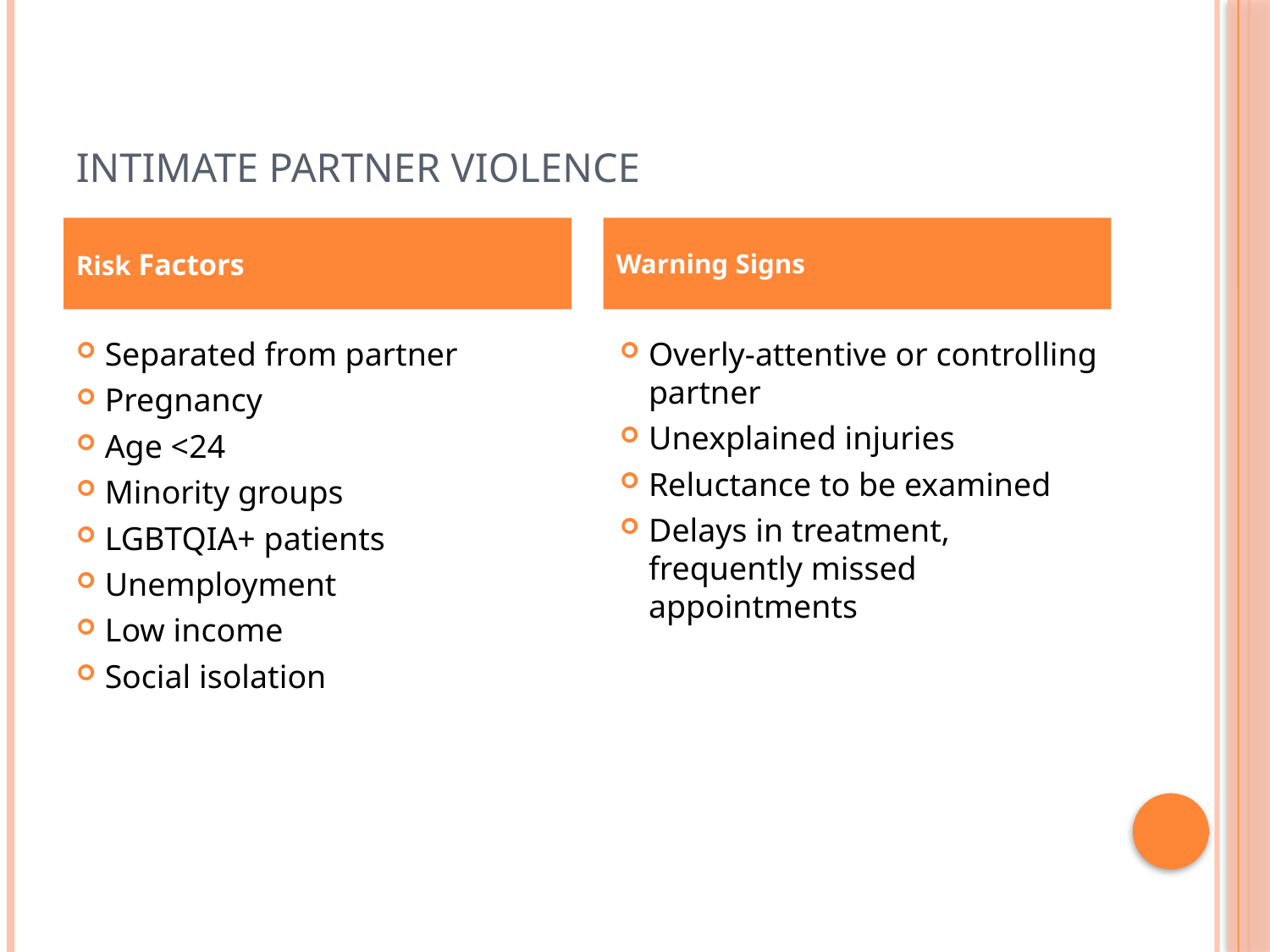

# Intimate Partner Violence
Risk Factors
Warning Signs
Separated from partner
Pregnancy
Age <24
Minority groups
LGBTQIA+ patients
Unemployment
Low income
Social isolation
Overly-attentive or controlling partner
Unexplained injuries
Reluctance to be examined
Delays in treatment, frequently missed appointments

## Slide 40
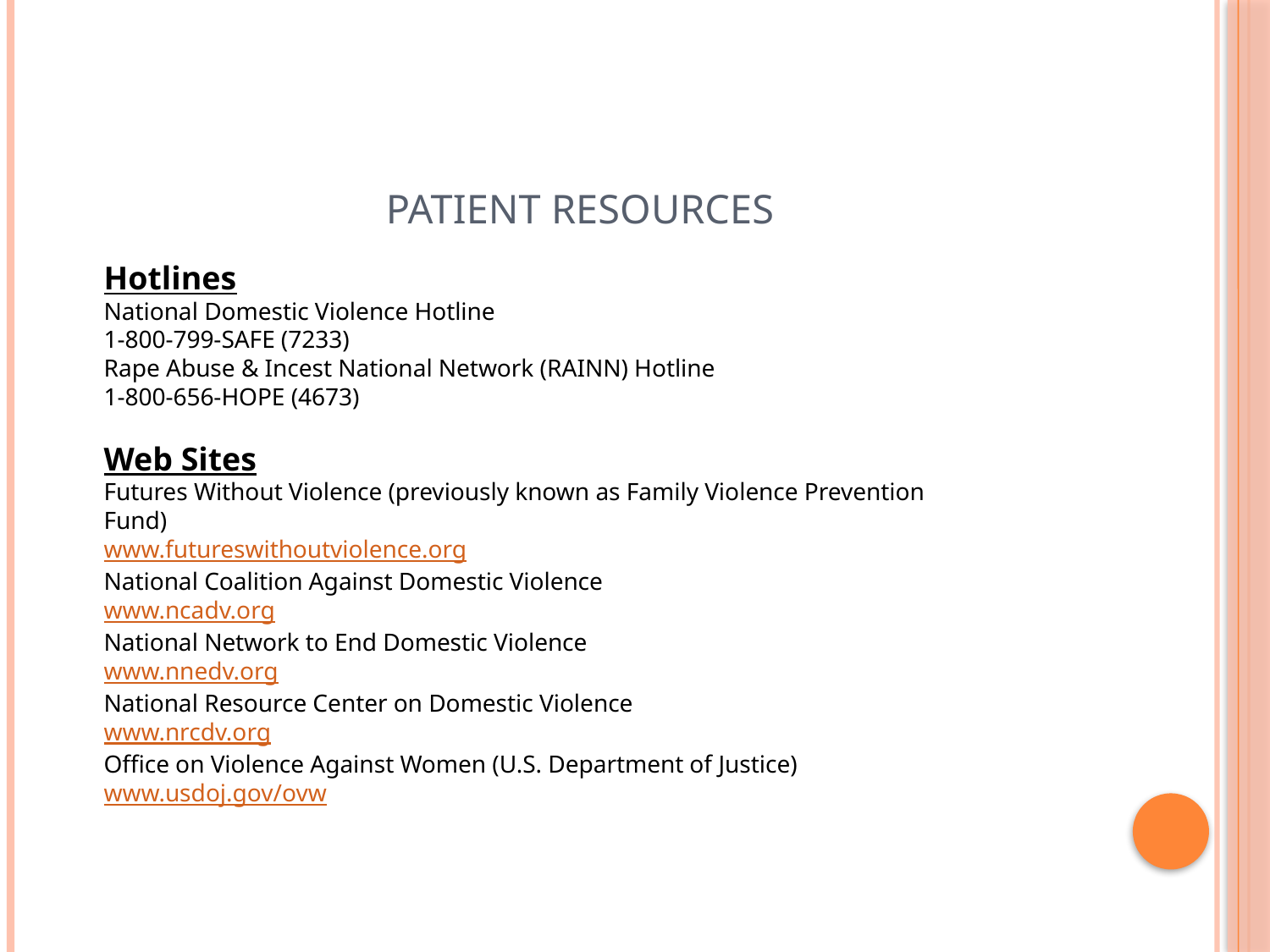

# Patient Resources
Hotlines
National Domestic Violence Hotline
1-800-799-SAFE (7233)
Rape Abuse & Incest National Network (RAINN) Hotline
1-800-656-HOPE (4673)
Web Sites
Futures Without Violence (previously known as Family Violence Prevention Fund)
www.futureswithoutviolence.org
National Coalition Against Domestic Violence
www.ncadv.org
National Network to End Domestic Violence
www.nnedv.org
National Resource Center on Domestic Violence
www.nrcdv.org
Office on Violence Against Women (U.S. Department of Justice)
www.usdoj.gov/ovw

## Slide 41
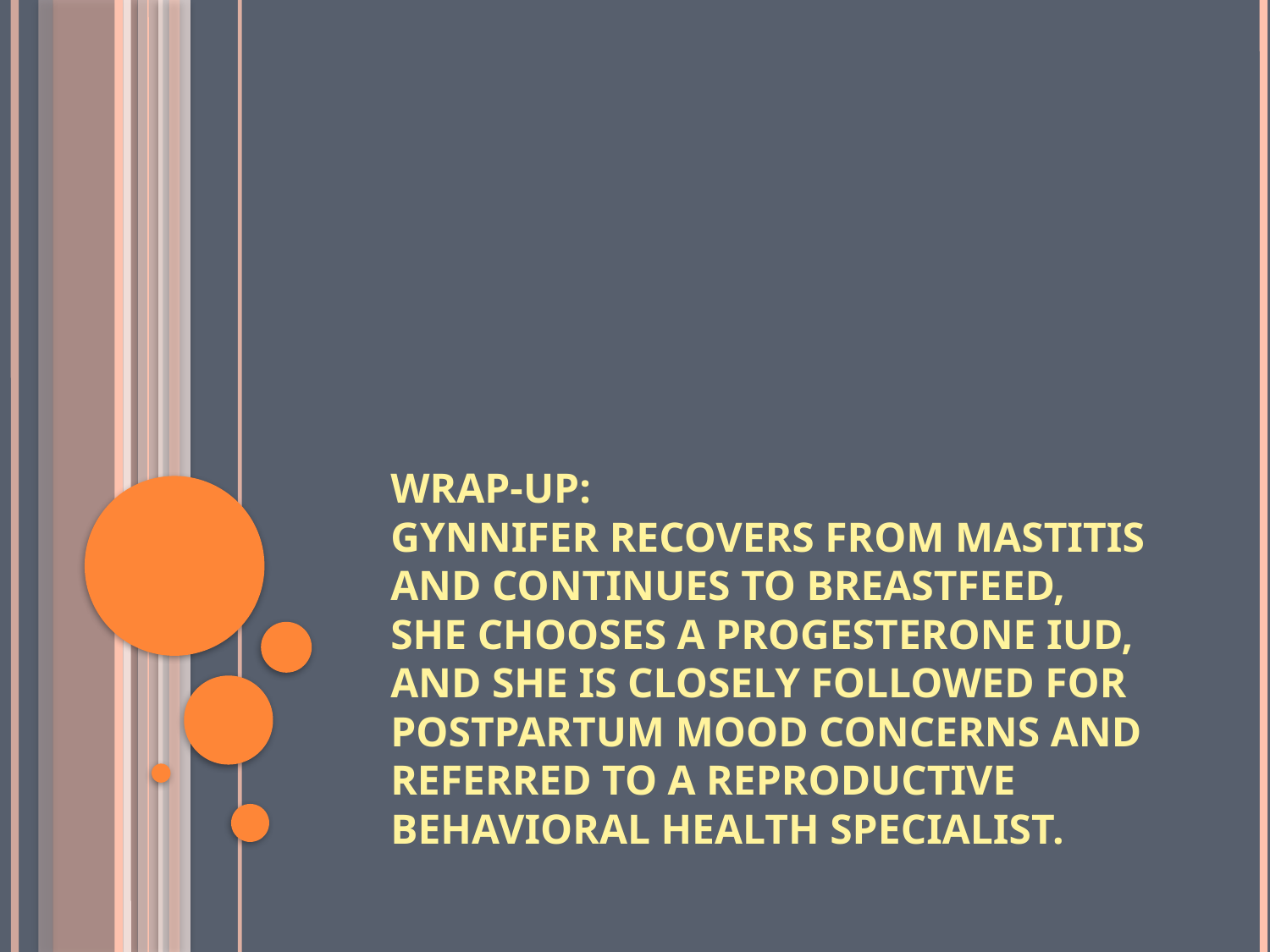

Wrap-Up:
Gynnifer recovers from mastitis and continues to breastfeed, she chooses a progesterone IUD, and she is closely followed for postpartum mood concerns and referred to a reproductive behavioral health specialist.

## Slide 42
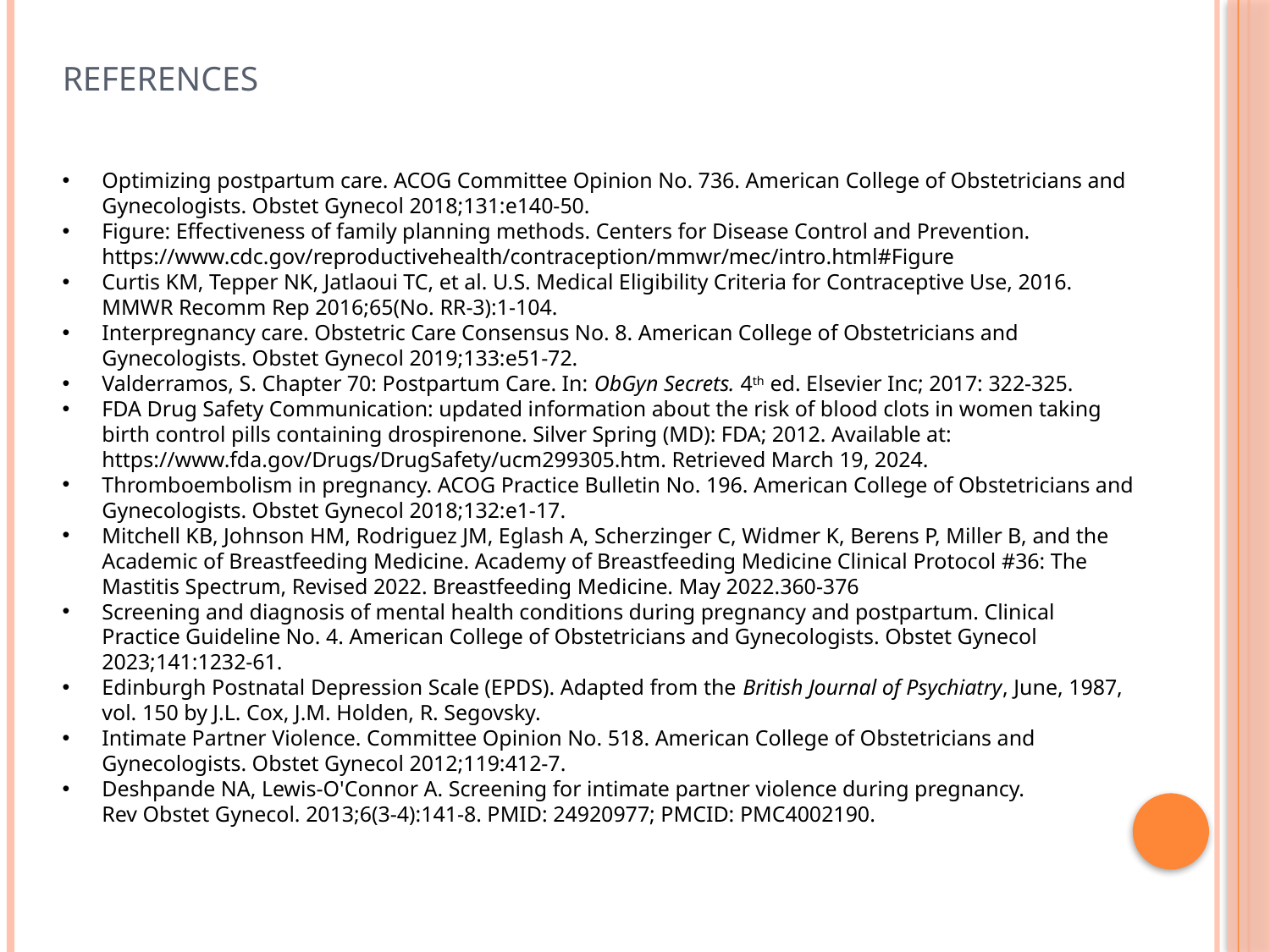

# References
Optimizing postpartum care. ACOG Committee Opinion No. 736. American College of Obstetricians and Gynecologists. Obstet Gynecol 2018;131:e140-50.
Figure: Effectiveness of family planning methods. Centers for Disease Control and Prevention. https://www.cdc.gov/reproductivehealth/contraception/mmwr/mec/intro.html#Figure
Curtis KM, Tepper NK, Jatlaoui TC, et al. U.S. Medical Eligibility Criteria for Contraceptive Use, 2016. MMWR Recomm Rep 2016;65(No. RR-3):1-104.
Interpregnancy care. Obstetric Care Consensus No. 8. American College of Obstetricians and Gynecologists. Obstet Gynecol 2019;133:e51-72.
Valderramos, S. Chapter 70: Postpartum Care. In: ObGyn Secrets. 4th ed. Elsevier Inc; 2017: 322-325.
FDA Drug Safety Communication: updated information about the risk of blood clots in women taking birth control pills containing drospirenone. Silver Spring (MD): FDA; 2012. Available at: https://www.fda.gov/Drugs/DrugSafety/ucm299305.htm. Retrieved March 19, 2024.
Thromboembolism in pregnancy. ACOG Practice Bulletin No. 196. American College of Obstetricians and Gynecologists. Obstet Gynecol 2018;132:e1-17.
Mitchell KB, Johnson HM, Rodriguez JM, Eglash A, Scherzinger C, Widmer K, Berens P, Miller B, and the Academic of Breastfeeding Medicine. Academy of Breastfeeding Medicine Clinical Protocol #36: The Mastitis Spectrum, Revised 2022. Breastfeeding Medicine. May 2022.360-376
Screening and diagnosis of mental health conditions during pregnancy and postpartum. Clinical Practice Guideline No. 4. American College of Obstetricians and Gynecologists. Obstet Gynecol 2023;141:1232-61.
Edinburgh Postnatal Depression Scale (EPDS). Adapted from the British Journal of Psychiatry, June, 1987, vol. 150 by J.L. Cox, J.M. Holden, R. Segovsky.
Intimate Partner Violence. Committee Opinion No. 518. American College of Obstetricians and Gynecologists. Obstet Gynecol 2012;119:412-7.
Deshpande NA, Lewis-O'Connor A. Screening for intimate partner violence during pregnancy. Rev Obstet Gynecol. 2013;6(3-4):141-8. PMID: 24920977; PMCID: PMC4002190.
