# Supplementary material for: A Flipped Classroom Case to Introduce OB/GYN Clerkship Students to Contraception, Postpartum Care, and Intimate Partner Violence Screening
Source: MedEdPORTAL. 2025 Apr 9;21:11505. doi: 10.15766/mep_2374-8265.11505 (PMC11978902; doi:10.15766/mep_2374-8265.11505)
Supplement: Supplementary file 1 — Student Prework.docxContraception Cards.pptxPostpartum Slides.pptxFacilitator Guide.docxFacilitator Survey.docxStudent Survey.docx [file mep_2374-8265.11505-s001.zip › _Educational Summary Report_11505.pdf]

MedEdPORTAL® The AAMC Journal of Teaching and Learning Resources

Original Publication · Open Access

A Flipped Classroom Case to Introduce OB/GYN Clerkship Students to Contraception, Postpartum Care, and Intimate Partner Violence Screening

Krista Wagoner, MD*, Angela Dempsey, MD, MPH, Faith Dunn, MD, Meredith Chardukian, MD

*Corresponding author: wagoner@musc.edu

Abstract

Introduction: The Association of Professors of Gynecology and Obstetrics core learning objectives for medical students include postpartum care, peripartum mood disorders, family planning, and intimate partner violence. **Methods:** This module uses student prework, session slides, and a facilitator guide for a 2-hour flipped classroom case that addresses these core learning areas for medical students on their obstetrics and gynecology clerkship. We assessed the module in a range of domains with five rotations of students who participated during their clerkship and facilitators who led the session. Data were collected from students and facilitators via voluntary participation in a survey made available after the session. Data are reported as proportion of respondents who agreed or strongly agreed. **Results:** Of 97 student participants, 57 completed the survey (59%). More than 85% agreed or strongly agreed that the session improved understanding or comfort in each of the learning objectives, and the majority agreed that the prework offered useful knowledge for the session (89%), that the session was interactive (91%), and that the session format was helpful in applying new knowledge (95%). Fifty-seven percent of facilitators responded; all agreed that the facilitator guide increased their confidence while teaching and that they spent less time preparing for the session than traditional didactics. Most (89%) agreed that the facilitator guide helped them promote active learning. **Discussion:** With favorable student and facilitator perceptions, this module provides a tool to address several core learning objectives for medical students in the context of an interactive flipped classroom module.

Keywords
Contraception, Postpartum, Women's Health, Clinical Reasoning/Diagnostic Reasoning, Flipped Classroom, Intimate Partner Violence, OB/GYN, Pregnancy, Childbirth, & the Puerperium

Educational Objectives

By the end of this activity, learners will be able to:

1. Outline the components of normal postpartum care.
2. Describe the mechanism of action and effectiveness of contraceptive methods.
3. Utilize national guidelines to determine the safety of contraceptive methods in key postpartum conditions.
4. Define basic evaluation and treatment of common postpartum abnormalities of the breast.
5. Identify wound complications and signs and symptoms of peripartum cardiomyopathy.
6. Compare the differences between postpartum blues, depression, and psychosis.
7. Summarize the approach to screening for intimate partner violence.

Citation:
Wagoner K, Dempsey A, Dunn F, Chardukian M. A flipped classroom case to introduce OB/GYN clerkship students to contraception, postpartum care, and intimate partner violence screening. *MedEdPORTAL*. 2025;21:11505.
https://doi.org/10.15766/mep_2374-8265.11505

Introduction

The Association of Professors of Gynecology and Obstetrics (APGO) has outlined core educational objectives for medical students completing obstetrics and gynecology clerkships.[1] These objectives specify that all medical students gain knowledge in postpartum care (objective 13), anxiety and depression in pregnancy and the postpartum period (objective 29), family planning (objective 33), and intimate partner violence (objective 58) as part of their medical education.

We developed a module that provides an interactive flipped classroom case featuring a patient presenting for a postpartum visit to address each of the educational objectives outlined above. Generally, flipped classroom and active learning

Copyright © 2025 Wagoner et al. This is an open-access publication distributed under the terms of the Creative Commons Attribution license.

techniques promote engagement, improve satisfaction, and facilitate application of new knowledge.[2-4] In the obstetrics and gynecology clerkship setting, a flipped classroom model was associated with high learner satisfaction, though it did not increase subject exam scores.[3] Clinical exposure to these essential concepts may be limited during the clerkship. Thus, our case-based learning module provides a valuable complement for students.

A search of *MedEdPORTAL* with the terms *contraception, contracep*, postpartum, postpar*,* and *intimate partner violence* identified resources with marginally overlapping content, including contraceptive pharmacology,[5] contraception counseling,[6-8] IUD management,[9] postpartum contraception options immediately after hospital discharge,[10] effect of obesity and bariatric surgery on contraception,[11] common breastfeeding problems,[12] screening for and treating postpartum depression,[13] and screening for and addressing intimate partner violence.[14,15] Compared to our module, most of these resources provide greater topical depth in a single area. Our session is distinct from this content because of its breadth, its design for obstetrics and gynecology clerkship students, the flipped classroom context utilizing a postpartum patient encounter, and the specific combination of our learning objectives. The content most closely related to this module is a team-based learning module aiming to teach students about normal physiology during pregnancy and the postpartum period and to help them differentiate between postpartum blues, depression, and psychosis.[16] Our session is novel because it also reviews contraception and intimate partner violence.

Methods

Educational Context

Our module was designed to teach basic contraception care; basic components of postpartum care, including an overview of wound complications, breast conditions, and postpartum cardiomyopathy; and screening principles for intimate partner violence. This module was designed to stand alone. However, in our clerkship, this module was integrated as the third session in a 5-week curriculum following a patient through prepregnancy, pregnancy, postpartum, and gynecologic care episodes. Two of the sessions in this curriculum have been previously published in *MedEdPORTAL*.[17,18]

The small-group sessions in our clerkship were facilitated by faculty representing both obstetrics and gynecology specialists and subspecialists. Each faculty facilitator received a brief orientation with clerkship leaders. The facilitators were granted access to materials at least 1 week prior to the session and were specifically instructed to review the facilitator guide. Our faculty spent 1-2 hours preparing the first time they facilitated the session and little to no time preparing once they were familiar with the content. Although this module could be used in a variety of educational settings, our clerkship conducted small-group sessions with six to eight students per facilitator. Each group met for approximately 2 hours in a setting equipped to project PowerPoint slides where the group openly discussed the case and conducted active learning activities. The session was modified for several years prior to evaluation to ensure the feasibility of delivering the material in 2 hours.

Prior to each small-group session, students were provided with specified prework designed to create a foundation of new knowledge that they could then expand and apply during the small-group session. The prework for this module (Appendix A) included several readings about contraception, postpartum care, mental health during pregnancy, and intimate partner violence. Students were instructed that prework for the session would likely take 3-4 hours to complete, though there was variability among students due to various learning and reading speeds.

This module began with a case presentation of a patient presenting for a postpartum visit 6 weeks following spontaneous vaginal delivery complicated by pre-eclampsia. The learning objectives and activities in this session were embedded in the context of a postpartum visit in the ambulatory setting. In addition to providing the facilitator with information and/or learning activities to include with each slide, the facilitator guide also directed facilitators to resources for additional in-depth learning on a given topic. For example, while displaying slide 5, the facilitator guide encouraged facilitators to task the group of students with organizing the contraception card set (Appendix B) in order of efficacy. The guide also prompted facilitators to ask follow-up critical thinking questions related to whether the students utilized typical-use or perfect-use failure rates to complete the task.

Implementing the module involved the following steps:

1. The facilitator instructed students assigned to the session to complete the provided prework materials (Appendix A).
2. The facilitator ensured the following materials were available for the live education session:
    - Contraception card set (Appendix B), which was designed to be printed, laminated, and used

Copyright © 2025 Wagoner et al. This is an open-access publication distributed under the terms of the Creative Commons Attribution license.

physically during the live education session. Laminated cards were then appropriate for reuse in future sessions.

- Equipment to display and project PowerPoint slides (Appendix C).
- The facilitator guide (Appendix D).

3. During the live education session, the facilitator displayed and advanced the PowerPoint slides. The in-depth facilitator guide accompanied the session slides. This guide highlighted teaching points to cover with each slide, suggested additional didactic information that facilitators could incorporate beyond the content on the slide, and prompted specific classroom activities designed to enhance student engagement, interaction, and active learning.
4. While not required for implementation of the education module, the optional facilitator survey (Appendix E) and the optional student survey (Appendix F) could be used to collect feedback.

Assessment

The module was evaluated through surveys measuring student and facilitator experiences. At the conclusion of each small-group session, student participants could scan a QR code projected on a slide to voluntarily and anonymously complete a survey. This survey measured student agreement with statements regarding the value of prework, the degree of session interactivity, the achievement of learning objectives, and the module's effectiveness in helping the student apply new knowledge. After their first scheduled session, facilitators received a direct email from the clerkship administrative coordinator asking them to complete a survey. The facilitator survey assessed facilitators' perceptions of interactivity, preparation time for teaching, and confidence in teaching the included topics with the aid of the in-depth facilitator guide. Student and facilitator outcomes were calculated as the proportion who agreed or strongly agreed with each variable.

Results

Students

Over five clerkship rotations, 57 students provided data out of the 97 who experienced the session as part of their curriculum, for a response rate of 59%. Table 1 reports student responses. More than 90% of students agreed or strongly agreed that the session improved understanding or comfort in each of the learning objectives except intimate partner violence screening. Eighty-six percent of students agreed that they were more comfortable screening for intimate partner violence following this session. The majority of students agreed that the prework provided useful knowledge for the session, that the session was interactive, and that the session format was helpful in applying new knowledge. One student disagreed with all questions.

Facilitators

Eight out of 14 facilitators (57%) provided data during the evaluation period. Table 2 outlines facilitator responses. One hundred percent of facilitators agreed that the facilitator guide increased their confidence teaching concepts in the session, including those outside of their specialty area. Likewise, 100% of facilitators agreed that they spent less time preparing for this education session compared to the 1-hour, traditional, passive, topical didactic lecture that our clerkship included in the past. All but one facilitator (89%) agreed that the facilitator guide helped them promote active learning during the session.

Discussion

The majority of students participating in this flipped classroom module agreed that it increased their understanding or comfort level with multiple core educational objectives specified by APGO for medical students. The small group of facilitators who provided data on the module also reported positive experiences.

The nature of obstetrics and gynecology clerkship education provides a broad range of patient-facing clinical encounters

Table 1. Students' Assessment of Sessions ($N = 57$)

Prompt[a]	No. (%) Agreeing or Strongly Agreeing
The assigned prework gave me the background knowledge needed to participate in activities during this session.	51 (89)
The session was interactive.	52 (91)
The format of this session will help me apply what I learned to patient care.	54 (95)
This session helped me understand the mechanism of action, effectiveness, and safety of contraceptive methods.	56 (98)
This session helped me understand the components of normal postpartum care.	54 (95)
After this session, I feel more comfortable diagnosing and treating common postpartum abnormalities of the breast.	54 (95)
After this session, I feel more comfortable evaluating a suspected postpartum complication.	53 (92)
This session helped me understand the difference between postpartum blues, depression, and psychosis.	54 (95)
After this session, I feel more comfortable screening for domestic violence.	49 (86)

[a]Rated on a 5-point scale (1 = *strongly disagree*, 5 = *strongly agree*).

Copyright © 2025 Wagoner et al. This is an open-access publication distributed under the terms of the Creative Commons Attribution license.

Table 2. Clinical Instructors' Assessment of Sessions ($N = 8$)

Prompt[a]	No. (%) Agreeing or Strongly Agreeing
The faculty guide for this session helped me facilitate active learning.	7 (88)
I spent less time preparing for this session than preparing for a traditional didactic lecture.	8 (100)
The information included in the faculty guide helped increase my confidence in teaching subjects outside of my area of expertise.	8 (100)

[a]Rated on a 5-point scale (1 = *strongly disagree,* 5 = *strongly agree*).

for all students, but the clinical experiences that each student encounters may vary substantially. For example, one student on a 6-week rotation may experience a positive intimate partner violence screen but not care for a patient with postpartum wound infection, while another may have the opposite exposure. Inclusion of modules such as this one with standardized prework, session slides, and a facilitator guide within a clerkship curriculum ensures that each student is exposed to standard core didactic information while acknowledging that comprehensively addressing each of these postpartum concerns at a single visit with a patient is unrealistic. The flipped classroom approach and built-in interactive activities enhance student engagement and satisfaction. Our data suggest that facilitators with a range of professional specialty experience can confidently facilitate the education session with the provided facilitator guide.

The module was evaluated in a specific context. It was embedded within a curriculum that included five sessions following the same patient across her reproductive life and was delivered in small groups of six to eight medical students who remained together across their obstetrics and gynecology clerkship. However, the module can stand alone, can be used with larger groups, and potentially can address learning needs of students in other health professions or at other stages of their medical training as it covers a broad array of topics rather than examining any one topic in depth. The student prework encourages a deeper dive into specific topics, yet we acknowledge that clerkship students are not expected to be clinical experts in any of the covered topics. We anecdotally note that the information covered in this session has provided groundwork and prompted more detailed discussions in clinical settings. For example, students have referenced the tiers of contraceptive efficacy covered in the session when seeing a patient with unplanned pregnancy.

Updates to clinical practice have necessitated a periodic review and update of the session. Oftentimes, this has required modifications to the presentation slides, facilitator guide, and student prework.We have found it helpful to highlight and convey to our facilitators any changes we make via email or at faculty meetings.

Our assessment of this module has limitations, including a relatively small convenience sample of students and facilitators and a modest response rate among both groups of stakeholders. Another limitation of our assessment of comfort levels and achievement of learning objectives is the use of students' self-reported agreement rather than objective outcome measures, such as a knowledge exam or faculty assessment of clinical skill. Finally, we acknowledge the limitation that this session is designed to provide students with exposure to broad knowledge of multiple high-yield clinical topics but requires supplementation with reading outside the small-group setting, intentional clinical practice facilitated by our clerkship's procedure and diagnosis log of required learning experiences, and additional patient-facing or simulated learning opportunities to achieve sufficient depth in some topics. We studied this session for 1 academic year; however, we have been teaching and updating it for 10 years at our institution due to high regard by both students and faculty.

In conclusion, this module provides a tool for medical educators to address several core learning objectives for medical students while maintaining student satisfaction and reducing teaching workload for facilitators. Next steps to consider for enhancing the module include adding more objective components to the assessment, including clinical skills assessment in an objective structured clinical examination or a knowledge-based exam. In addition, preceptor evaluations of students at the end of their clerkships could assess application of clinical skill and knowledge gained during the module. Such evaluation methods would facilitate continued quality improvement of module content over time to achieve the stated learning objectives. Because the module is designed to provide a breadth of didactic information on multiple core topics, educators implementing it should consider supplementing the module with other intentional learning activities, such as additional reading or video content, patient encounters, and simulated learning opportunities, to provide additional depth.

Appendices

A. Student Prework.docx

B. Contraception Cards.pptx

Copyright © 2025 Wagoner et al. This is an open-access publication distributed under the terms of the Creative Commons Attribution license.

C. Postpartum Slides.pptx

D. Facilitator Guide.docx

E. Facilitator Survey.docx

F. Student Survey.docx

All appendices are peer reviewed as integral parts of the Original Publication.

Krista Wagoner, MD: Associate Professor, Department of Obstetrics and Gynecology, Medical University of South Carolina College of Medicine; ORCID: https://orcid.org/0000-0002-2626-7470

Angela Dempsey, MD, MPH: Professor, Department of Obstetrics and Gynecology, Medical University of South Carolina College of Medicine

Faith Dunn, MD: Third-Year Resident, Department of Obstetrics and Gynecology, Medical University of South Carolina College of Medicine

Meredith Chardukian, MD: Obstetrician Gynecologist, Independent Practice

Disclosures
None to report.

Funding/Support
None to report.

Ethical Approval
Reported as not applicable.

References

1. APGO Medical Student Educational Objectives for students. Association of Professors of Gynecology and Obstetrics. Accessed February 28, 2025. https://apgo.org/page/msostudent
2. Chen F, Lui AM, Martinelli SM. A systematic review of the effectiveness of flipped classrooms in medical education. *Med Educ*. 2017;51(6):585-597. https://doi.org/10.1111/medu.13272
3. Morgan H, McLean K, Chapman C, Fitzgerald J, Yousuf A, Hammoud M. The flipped classroom for medical students. *Clin Teach*. 2015;12(3):155-160. https://doi.org/10.1111/tct.12328
4. Redmond CE, Healy GM, Fleming H, McCann JW, Moran DE, Heffernan EJ. The integration of active learning teaching strategies into a radiology rotation for medical students improves radiological interpretation skills and attitudes toward radiology. *Curr Probl Diagn Radiol*. 2020;49(6):386-391. https://doi.org/10.1067/j.cpradiol.2019.07.007
5. Ottolenghi J, Athauda G, Stumbar SE, Kashan SB, Lupi C. Contraceptive pharmacology and risk communication: a case-based flipped classroom exercise. *MedEdPORTAL*. 2019;15:10790. https://doi.org/10.15766/mep_2374-8265.10790
6. Lupi CS, Mechaber AJ. Objective structured clinical examination: contraceptive counseling for long acting reversible methods. *MedEdPORTAL*. 2011;7:9021. https://doi.org/10.15766/mep_2374-8265.9021
7. Tang I, Rupley D. An interactive curriculum to teach person-centered contraceptive counseling. *MedEdPORTAL*. 2023;19:11368. https://doi.org/10.15766/mep_2374-8265.11368
8. Ortiz Worthington R, Oyler J, Pincavage A, Abou Baker N, Saathoff M, Rusiecki J. A novel contraception counseling and shared decision-making curriculum for internal medicine residents. *MedEdPORTAL*. 2020;16:11046. https://doi.org/10.15766/mep_2374-8265.11046
9. Field C, Benson LS, Stephenson-Famy A, Prager S. Intrauterine device training workshop for preclinical medical students. *MedEdPORTAL*. 2019;15:10841. https://doi.org/10.15766/mep_2374-8265.10841
10. Abraham C. Successful implementation of a medical student postpartum follow-up phone call project. *MedEdPORTAL*. 2021;17:11109. https://doi.org/10.15766/mep_2374-8265.11109
11. Cook J, Puckett HL, Steinauer JE. Management of obesity during pregnancy and periconception: case-based learning for OB/GYN clerkships. *MedEdPORTAL*. 2021;17:11129. https://doi.org/10.15766/mep_2374-8265.11129
12. McLeod K, Waller J, Wyatt TR. Using videos to teach medical learners how to address common breastfeeding problems. *MedEdPORTAL*. 2021;17:11136. https://doi.org/10.15766/mep_2374-8265.11136
13. Freerksen S, Brooks W, Siddiqui S. Post-partum depression: diagnosis, screening, and treatment. *MedEdPORTAL*. 2015;11:10070. https://doi.org/10.15766/mep_2374-8265.10070
14. Schrier MW, Rougas SC, Schrier EW, Elisseou S, Warrie S. Intimate partner violence screening and counseling: an introductory session for health care professionals. *MedEdPORTAL*. 2017;13:10622. https://doi.org/10.15766/mep_2374-8265.10622
15. Insetta ER, Christmas C. A novel intimate partner violence curriculum for internal medicine residents: development, implementation, and evaluation. *MedEdPORTAL*. 2020;16:10905. https://doi.org/10.15766/mep_2374-8265.10905

Copyright © 2025 Wagoner et al. This is an open-access publication distributed under the terms of the Creative Commons Attribution license.

16. Sward LB, Tariq SG. Maternal-fetal physiology, intrapartum care, postpartum care: a team-based learning module for normal obstetrics. *MedEdPORTAL*. 2019;15:10856. https://doi.org/10.15766/mep_2374-8265.10856

17. Wagoner K, Dempsey A, Wade J, Dunn F. Evaluation and management of early pregnancy: a flipped classroom case for OB/GYN clerkship students. *MedEdPORTAL*. 2023; 19:11297. https://doi.org/10.15766/mep_2374-8265.11297

18. Wagoner K, Dempsey A, Dunn F, Hemphill A. Normal pregnancy care and physiology and select pregnancy complications: a flipped classroom case for the OB/GYN clerkship. *MedEdPORTAL*. 2024;20:11413. https://doi.org/10.15766/mep_2374-8265.11413

Received: June 27, 2024
Accepted: January 8, 2025
Published: April 9, 2025

Copyright © 2025 Wagoner et al. This is an open-access publication distributed under the terms of the Creative Commons Attribution license.
